# Historical rise of waterpower initiated the collapse of salmon stocks

**DOI:** 10.1038/srep29269

**Published:** 2016-07-20

**Authors:** H. J. R. Lenders, T. P. M. Chamuleau, A. J. Hendriks, R. C. G. M. Lauwerier, R. S. E. W. Leuven, W. C. E. P. Verberk

**Affiliations:** 1 Institute for Water and Wetland Research, Department of Environmental Science, Radboud University, P.O. Box 9010, 6500 GL Nijmegen, the Netherlands; 2 Rijkswaterstaat, Centre for Water, Traffic and Environment, P.O. Box 17, 8200 AA Lelystad, the Netherlands; 3 Department of Archaeology, Cultural Heritage Agency, Ministry of Education, Culture and Science, P.O. Box 1600, 3800 BP Amersfoort, the Netherlands; 4 Institute for Water and Wetland Research, Department of Animal Ecology and Physiology, Radboud University, P.O. Box 9010, 6500 GL Nijmegen, the Netherlands

## Abstract

The collapse of Atlantic salmon (*Salmo salar*) stocks throughout North-Western Europe is generally ascribed to large-scale river regulation, water pollution and over-fishing in the 19^th^ and 20^th^ century. However, other causes have rarely been quantified, especially those acting before the 19^th^ century. By analysing historical fishery, market and tax statistics, independently confirmed by archaeozoological records, we demonstrate that populations declined by up to 90% during the transitional period between the Early Middle Ages (c. 450–900 AD) and Early Modern Times (c. 1600 AD). These dramatic declines coincided with improvements in watermill technology and their geographical expansion across Europe. Our extrapolations suggest that historical Atlantic salmon runs must have once been very abundant indeed. The historical perspective presented here contributes to a better understanding of the primary factors that led to major declines in salmon populations. Such understanding provides an essential basis for the effective ecological rehabilitation of freshwater ecosystems.

Marine fishing has resulted in severe and wide ranging ecological consequences dating back to prehistoric times^1^,^2^,^3^. To date, comparable early anthropogenic impacts on fish stocks of freshwater ecosystems have not been established in quantitative terms. The main contributor to the decline in ecological integrity of the larger river systems in North-Western Europe, *viz.* the rivers Rhine, Meuse, Thames and Seine, is assumed to be 19^th^ and 20^th^ century pollution, over-fishing and river regulation^4^. During the previous two centuries, migratory species like common sturgeon (*Acipenser sturio*), houting (*Coregonus oxyrinchus)*, allis shad (*Alosa alosa*), twaite shad (*Alosa fallax*) and Atlantic salmon (*Salmo salar*) have become extinct or endangered^5^,^6^. This decline has resulted in the designation of the Atlantic salmon as a flagship species and ambassador for ecological restoration in the River Rhine and its tributaries^4^. However, despite the application of several rehabilitation measures aimed at mitigating and compensating for the adverse effects of pollution, fishing and river regulation, restocking of Atlantic salmon appears far from effective throughout Europe. A previously ignored and potentially limiting factor may be the fact that declines in salmon populations began much earlier in history than is currently recognized, i.e. well before the start of large-scale river regulation^7^,^8^. Essential components may thus be overlooked in rehabilitation projects accounting for the limited success of restocking programs. Indeed, many pre-19^th^-century sources appear to perceive salmon stocks to have been “much larger in preceding times”. However, these anecdotal observations have not been substantiated with quantitative evidence.

Although it is accepted that watermills had a tremendous impact on the geomorphology of river and stream habitat^9^, the potential negative impacts of watermills has not been thoroughly analysed in quantitative terms in ecological research. Generally, the few scientific articles describing the historical effects of watermills on aquatic ecosystems do this in a qualitative or anecdotal manner, at local geographical scales^7^,^10^ or examine relatively short periods in history^11^. In this article we examine potential watermill related salmon declines in North-Western Europe at a temporal scale of many hundreds of years, and demonstrate the existence of a relationship between historical economic activities and the degradation of stream ecosystems. Quantitative proxies for Atlantic salmon abundance (prices, taxes and landings), independently confirmed by archaeological data, unambiguously tie the collapse of salmon stocks to the rise and operation of waterpower from the Middle Ages onwards.

## Salmon declines through the ages

**Figure 1:**
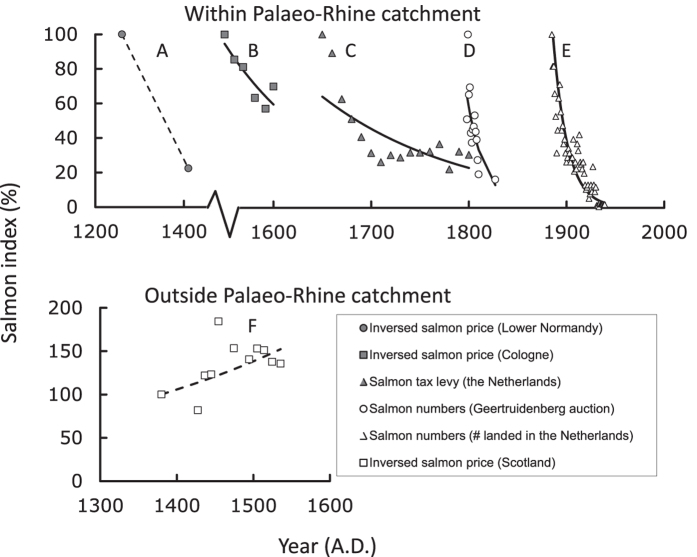
Indexed declines of salmon stocks through the centuries. (A) trends in the inverse of salmon prices in Lower Normandy, France (corrected using the trend in mean price of mutton, pork and partridge); (B) trends in the inverse of salmon prices in Cologne, Germany (corrected using changes in the price of other fish species); (C) trend in the tax levied on salmon fishery in the Netherlands; (D) salmon brought to the Geertruidenberg fish auction, the Netherlands; (E) salmon landed in the Netherlands; (F) trends in the inverse of salmon prices exported from Scotland. For each data source with more than two data points, the fitted exponential regression y = a·e^(b·x)^ yielded significant models (min F_1,4_ = 11.29; *P* = 0.028) with exponential declines (−0.007 ≤ b ≤ −0.068). The only exception was the data series from Scotland where the slope of the fitted regression was non-significant (F_1,9_ = 4.58; *P* = 0.061, b = +0.003).

Our research area encompasses the former Palaeo-Rhine catchment, once the single largest river system in NW-Europe, comprising present-day rivers like the River Rhine itself, as well as the Rivers Meuse, Scheldt, Thames and Seine^12^,^13^. Extinct and extant populations of Atlantic salmon in many of NW-Europe’s rivers once thus shared the same river system as their habitat. Long-term temporal trends in historical proxies for Atlantic salmon abundance in this area (prices, taxes and landings, Fig. 1), show a steady decline since medieval times and possibly earlier. The decline in salmon stocks indicated by these historical proxies is confirmed by steep decline in catches recorded at the medieval salmon fishery at Montchaton, France. Here catches of 300–350 per year in the 14^th^ century steeply declined to only three or four fish by the end of the 15^th^ century^10^. Deterioration of salmon stocks appears to have been in process already in Late Medieval France (13^th^–15^th^ centuries; Fig. 1A) and continued (Fig. 1B–E) until evidence of salmon presence disappears from most tributaries of the (Palaeo-)Rhine in the 20^th^ century. On rare occasion, populations may have recovered to a certain extent or briefly stabilized for relatively short periods (e.g., in the 18^th^ century; Fig. 1C). An alternative explanation could be that improved fishery techniques may have resulted in periods of consistent catches despite declining stocks^14^. However, the general picture emerging is one of declining salmon stocks during all time periods with the strongest decline occurring well before the 19^th^ century. In other European regions, however, salmon stocks remained stable. Prices paid for salmon in the 14^th^–16^th^ centuries in Scotland^15^, for instance, reveal a stable supply of salmon from local fisheries for 150 years (Fig. 1F). These Scottish data also demonstrate that Atlantic salmon stocks were sustained despite intensive fishing, as also seems to be the case for Alaskan sockeye salmon^16^. Collectively, this means that neither recent management of river habitat nor the presence of fisheries can be held solely responsible for the salmon’s extirpation in the Palaeo-Rhine catchment, although these factors probably eliminated the remaining stock in the 19^th^ and 20^th^ centuries^17^. Similar large declines in salmonid abundance (>99%) have been reported for Swedish land-locked populations over 200 years (1809–1970) following the construction of dams and the destruction of freshwater spawning and rearing habitats^18^.

Archaeozoological records available for the Netherlands, Belgium and France provide an independent line of evidence suggesting that salmon populations strongly declined in the Middle Ages corroborating the above described trend obtained from written historical records (see Supplementary information Table S1). The N_salmon_/N_pike_-ratio increases from the early farming period (Late Middle Neolithic to Early Bronze Age) to the Early Middle Ages due to a relative increase in the number of preserved salmon remains (Table 1, see methods section regarding our assumption of pike as a reference metric), as expected for stable stocks. However, during the transition from the Early to the Late Middle Ages, a sharp decrease in the number of sites containing salmon remains relative to sites with pike remains indicates a pronounced decrease in salmon stocks. There is a wealth of qualitative evidence in historical sources from the High and Late Middle Ages that this decline cannot be explained by a decreasing prevalence for salmon as food, nor can intensive fisheries be held solely responsible for this decline as relatively stable fishery yields are recorded from other regions of Europe, especially Scotland^15^ (Fig. 1). After the Late Middle Ages, the ratio suggests that stocks increased again. This, however, does not indicate a recovery, but is instead consistent with a rising import of salmon to NW-Europe from the 14^th^ century onwards, notably from Scotland^15^,^19^. The archaeozoological records presented here confirm our quantitative historical data, providing multiple lines of evidence that indicate that salmon stocks of the Palaeo-Rhine catchment suffered their most dramatic decline in the Middle Ages.

### Medieval rise of waterpower

**Figure 2:**
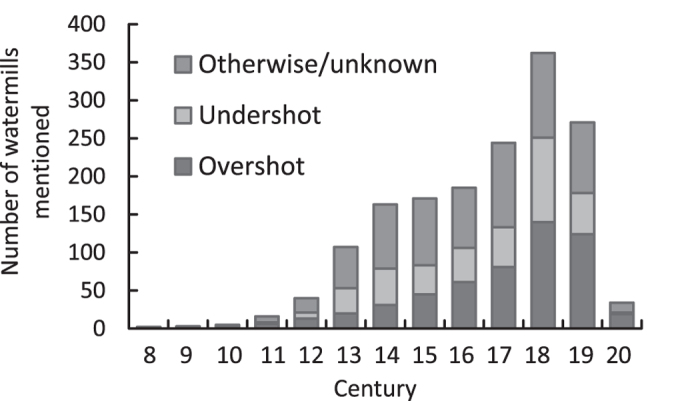
Numbers of newly constructed watermills in the 8^th^ to the 20^th^ century in the Rhine and Meuse catchment areas (watermills first mentioned in written sources taken as proxies for development per century).

In the Palaeo-Rhine catchment many watermills with vertical water wheels were in operation in the Middle Ages, in contrast to the much smaller horizontal water wheel mills, mostly operated in peripheral regions in Europe such as Ireland, Scotland and Scandinavia^20^,^21^,^22^,^23^,^24^,^25^. Vertical water wheel mills are associated with hydropower dams and reservoirs that create conditions for sufficient fall for water to drive the water wheels. These dams allowed watermills to be constructed in streams with relatively low water flow (0.03–0.3 m^3^s^−1^). Improvements in construction techniques^20^ enabled mills to be constructed in increasingly larger, higher order rivers. The use of watermills spread rapidly in the Palaeo-Rhine catchment from the 11^th^ century onwards^20^,^26^ (see Fig. 2 for data from the Rhine and Meuse catchments in Germany, Belgium and the Netherlands). In the Meuse catchment, mill presence peaks in the 18^th^ century. In the Rhine catchment, a greater proportion of mills was constructed in earlier centuries. In both catchments, the number of newly constructed watermills declined sharply in the 20^th^ century when other modes of power generation arose. While several watermills present in historical records no longer exist, they have had a lasting impact on streams, either in the form of extant dams or through geomorphologic alterations of the streambed and the stream valley^9^,^27^.

Concurrent with their fast geographical spread, a rapid increase in the density of watermills also occurred. The Domesday Book^25^,^28^, which contains the result of a census completed in 1086 and commissioned by William the Conqueror, gives details of the number of watermills existing in 11^th^ century England. By this time, watermills were already numerous with estimated numbers ranging from 5,600 to more than 6,000^29^,^30^. In the first half of the 18^th^ century, some regions featured streams that approached or even reached watermill saturation^20^, meaning that stream stretches between two subsequent watermills did not provide enough fall or momentum to allow for the construction of additional mills. Water mill numbers amounted to as much as 12 per linear kilometre along the River Mersey downstream of Manchester (60 watermills on a stretch of 5 km) to even 20 per linear kilometre at Vienne in eastern France (100 watermills on a stretch of 5 km).

### Ecological impacts of watermills

**Figure 3:**
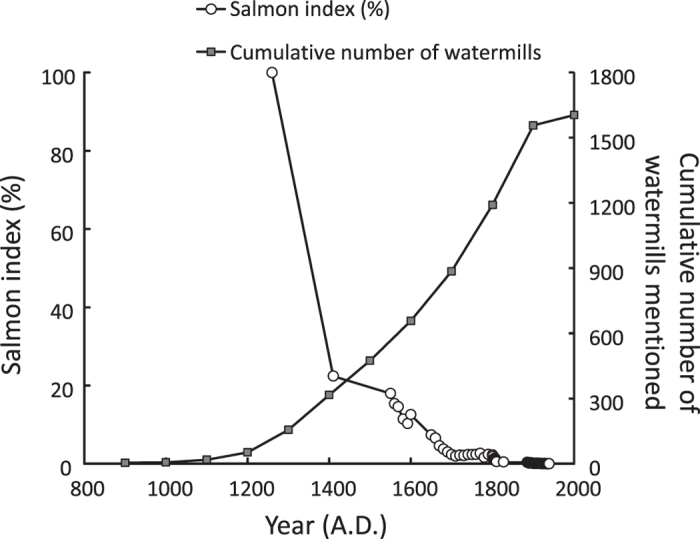
Aggregated indexed salmon decline (combined information from Fig. 1) in the Palaeo-Rhine catchment (circles) mirrored by increasing cumulative numbers of watermills in the Dutch, Belgian and German part of the catchment (squares). The trends are significantly negatively correlated (r = −0.813; *P* < 0.0001).

Increasing numbers of watermills are strongly negatively correlated with decreasing stocks of Atlantic salmon in the Palaeo-Rhine catchment (Fig. 3). By the end of the Middle Ages, salmon stocks in the Palaeo-Rhine catchment appear to have decreased by about 75%. At the end of the 18^th^ century, when the number of mills was at its peak, stocks had dwindled to less than 5%. Extrapolations spanning all time periods from the early Middle Ages to 1900 AD indicate cumulative stock declines exceeding 99.9% (Fig. 3).

**Figure 4:**
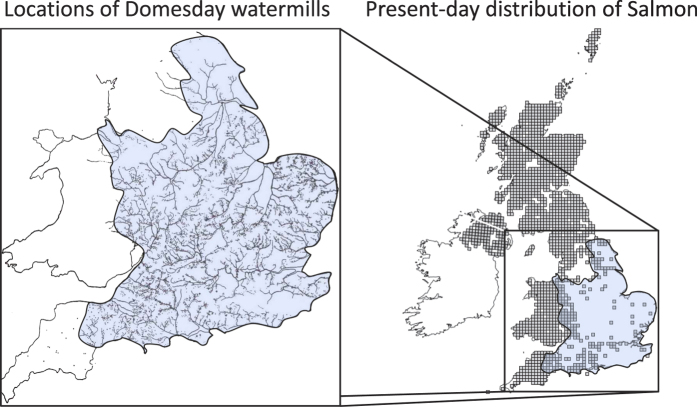
Spatial correspondence between watermill locations and the present day distribution of Atlantic salmon in the United Kingdom. The left panel^18^ shows an inset with each dot representing a water mill. The right panel shows a distribution map (© Crown copyright and database rights 2011 Ordnance Survey [100017955]) of Atlantic salmon (filled squares). Data courtesy of the NBN Gateway with thanks to all the data contributors. https://data.nbn.org.uk/Taxa/NBNSYS0000188606/Grid_Map (Accessed May 2014). The NBN and its data contributors bear no responsibility for the further analysis or interpretation of this material, data and/or information.

We also demonstrate a strong spatial concordance between salmon abundance and watermills in England, a region where such data are available. Where watermills with vertical waterwheels were present, salmon were absent and vice versa (Fig. 4). Qualitative evidence also shows a spatial correspondence between salmon abundance and watermills. In the counties of Domesday England, where densities of watermills were considerably lower, or where only horizontal water wheels were present, dues for fisheries were sometimes paid in salmon (notably in Devonshire, Gloucestershire, Herefordshire and Cheshire). In contrast, no mention of rents paid in salmon is made for counties featuring high densities of mills^25^,^28^.

The environmental changes that resulted from the proliferation of watermills and the unique life-history strategy of salmon provide a deeper insight into the mechanism that led to salmon decline in Europe, implied by the temporal and spatial patterns documented above. Atlantic salmon produce relatively few, but large (5–7 mm) eggs, that require intensive brood care that involves digging redds and covering eggs with gravel. Since life-history traits of species are all interlinked^31^,^32^, salmon also show a series of exacting requirements with regards to stream oxygenation, flow velocity, water depth and substrate particle size for spawning and rearing habitat^33^,^34^. In order to access lower order streams that fulfil these requirements and provide suitable spawning grounds, Atlantic salmon, like Pacific salmon, migrate over great distances. The journey is made only once by most adult fish, driven by imprinted scent-based homing behaviour and rheotaxis. Migrating salmon converge at spawning grounds and take part in a single massive reproductive event after which most individuals die^31^. As a result, Atlantic salmon populations in different rivers and tributaries are adapted to local conditions, and show distinct genetic features^35^,^36^, differing in spawning period, age at spawning, age at out-migration, and body size in relation to the geographical position of natal streams. These characteristics are the same for salmonid populations the world over and are considered a keystone of salmonid conservation^37^,^38^,^39^,^40^. The demanding life-history strategy of Atlantic salmon makes them extremely vulnerable to environmental changes in their freshwater spawning and rearing habitats, and may partly explain the large inter-population variations observed in phenology and migration distance which may act as bet-hedging mechanisms^41^. The Atlantic salmon’s life-history strategy can thus explain why, when compared to other anadromous fish species, it is relatively vulnerable to both changes in the quality of spawning and rearing sites, and alterations to hydrological connectivity along migratory pathways.

Watermills have severely impacted the geomorphology of lower order streams^9^,^27^ resulting in equally severe ecological impacts on Atlantic salmon. Siltation of gravel beds occurs at locations upstream of mill dams impacting the quality of spawning grounds. Erosion from deforestation and agricultural practices also negatively impacts spawning habitat through siltation; both are activities for which water mills can be considered proxies. Fast flowing, well-oxygenated streams with coarse substrates were replaced by standing water millponds that are much weedier, warmer and have lower dissolved oxygen minima, significantly reducing their suitability for parr and smolts. In view of the fact that many streams and rivers from approximately 1500 AD onwards were saturated or virtually saturated with watermills^20^, it may be expected that suitable spawning and rearing habitats were lost over vast stretches of streams, undoubtedly strongly reducing the production of smolts^34^. In addition, upstream spawning sites, for example those located in the Vosges and Eiffel for the Rhine catchment and in Wallonia and eastern France for the Meuse catchment, became virtually unreachable for upstream migrating adult fish. Moreover, the operation of mills is likely to have reduced the survival of out-migrating smolt^18^. Furthermore, much historical evidence indicates that watermills facilitated the salmon fishery, further reducing the survival of upstream migrating adults^25^,^28^. Finally, increased stream pollution resulting from the discharge of the (semi-industrial) by-products of the watermills (e.g., waste products discharged to the water from flax and hemp retting, and paper, paint and lead processing watermills)^20^ will have reduced smolt production, further impacting salmon populations.

It is obvious that watermills constructed early in the history of our study at more down-stream locations had proportionally larger impacts on salmon populations than those that were built later at more up-stream locations. Those earlier constructed mills presented obstacles that prevented migration to a larger proportion of upstream spawning grounds by cutting off a larger proportion of the river basin to migrating salmon. The strongest decline in salmon stocks therefore coincides with medieval watermill construction. Watermills constructed later contribute relatively little to worsened accessibility to and loss of salmon spawning grounds and nursery habitat.

### Conclusions and management implications

We conclude that watermills initiated the collapse of Atlantic salmon stocks in the Palaeo-Rhine catchment as a result of their impact on salmon reproduction and migration. In view of the steep declines of salmon stocks in each of the time periods considered (Fig. 1), Atlantic salmon must have originally been highly abundant. Cumulative declines in stock have likely exceeded 99.9%, indicating that historic Atlantic salmon runs in the Palaeo-Rhine catchment must have once been very impressive. The collapse of salmon stocks must have resulted in multiple negative effects that reverberated throughout the food web, exemplified by the deprivation of river ecosystems from an important nutrient subsidy. Depending upon the length of the river or stream traversed, between 65% and 100% of the adults commonly die before or after spawning due to predation or physical exhaustion. A straightforward and conservative calculation indicates that this level of mortality must have resulted in an annual biomass input of at least 230,000 metric tons of biomass to the upper reaches of the present-day defined Rhine catchment around the year 1200 AD. This roughly corresponds to 6,500 metric tons of nitrogen and 845 metric tons of phosphorus [Mean weight 3.5 kg *100,000 (conservative estimate of adult salmon present yearly in the Rhine at the end of the 19^th^ century)^42^ *1,000 (relative abundance in 1200 AD compared to the end of the 19^th^ century, based on our calculations, see Fig. 3) *65% (assuming a high mean percentage of 35% of salmon returning to the sea after reproduction) = 230,000,000 kg salmon biomass added to the Rhine ecosystem annually, which corresponds to roughly 6,500 metric tons of nitrogen and 845 metric tons of phosphorus (assuming comparable contents of N and P to Pacific salmon, *Oncorhynchus* spp.)^43^. Similar calculations for the year 1600 AD still yield 29,000,000 kg of salmon biomass added annually, corresponding with 845 metric tons of nitrogen and 110 metric tons of phosphorus.]. Thus, Atlantic salmon once contributed extremely large quantities of marine-derived nitrogen and phosphorus to the upper reaches of the Palaeo-Rhine catchment. This marine subsidy constituted a vital resource for large predators and scavengers such as brown bear (*Ursus arctos*) and white-tailed eagle (*Haliaeetus albicilla*)^44^, similar to the contribution by anadromous fish world-wide^45^,^46^. The loss of this constant supply of nutrients must have critically altered the functioning of these riparian ecosystems.

The Atlantic salmon’s dependence on the whole river system, from its headwater streams to its estuary, makes it the perfect flagship species for the ecological rehabilitation of rivers. If the relationship between habitat and salmon populations, as described in our work, is considered, it becomes clear that the restoration of the numerous tributaries of the stream/river systems, that were once used by salmon for reproduction, is a critical element in river and stream rehabilitation, in addition to the restoration of migration possibilities in the main channels. Rehabilitation of river tributaries is a vital element without which salmon recovery becomes impossible. The historical perspective presented here suggests that encouragement of fish migration through modifications to weirs and dams in the main channel and the creation of gravel beds will not, on its own, be sufficient to ensure the recovery of viable stocks of Atlantic salmon. The historical perspective also highlights that, for salmon to thrive, restoration efforts need to take into account the entire freshwater system within river catchments, including the capillaries, smaller and larger streams, the main channel and its distributaries, and connections to the sea. The Atlantic salmon’s history helps us to broaden our scope, both in space and time.

## Methods

### Data collection

Data from written historical sources were required to meet the following quality criteria for inclusion in the analyses:

(1) Data relate to local salmon stocks, i.e. only “fresh” salmon, are considered, and barrelled and dried salmon or salmon for which freshness cannot be confirmed are excluded.

(2) Data refers to periods of time in which no significant changes occurred with regard to relevant methodologies (e.g., no large changes in catch techniques or tax levy regimes). If such changes occur then corrections to the data with regard to such changes are applied.

(3) Times series contain sufficient data points and are sufficiently extensive to prevent stochastic effects from overwhelming clear trends.

(4) If prices are used as proxies for salmon abundance these should be corrected for deflation/inflation. This implies that prices for other protein-rich food (preferably other fish) should be available for the same period and location.

Five data series were considered to be suitable. A: the price of salmon in Normandy, France in the periods 1260/70-1410/20 AD^10^,^47^; B: the price of salmon in Cologne, Germany in the period 1550–1600 AD^48^; C: records of taxes levied on salmon fisheries in the Netherlands in the period 1650–1800 AD^49^; D: the number of salmon brought to the Geertruidenberg fish market, the Netherlands 1798–1827 AD^50^; E: salmon fishery statistics, the Netherlands 1885–1939 AD^51^. Additional data for the late 19^th^ and 20^th^ century were available and used in the analysis. Of this data, only catch statistics from the downstream parts of the Rhine in the Netherlands were used, which we considered representative of the situation in other parts of the Palaeo-Rhine catchment at the time. For purposes of comparison, we included a data series originating from outside our area of interest: F: prices paid for salmon meant for export from Scotland 1311–1541 AD^15^. These data allowed us to compare the Palaeo-Rhine catchment data with data originating from an area without widespread watermill usage before the era of extensive riverine management, but featuring comparably intensive fisheries. To calculate the development of salmon stocks in the first period, we used the prices as given by Halard^10^, which represent averages of 10-year ranges of prices paid for salmon and other protein rich food in the periods 1260–1270 AD and 1410–1420 AD. The time series for the price of salmon in Normandy thus consisted of two data points each representing 10 year averages, compensating for potential stochastic effects. The resulting price-based quantitative trend indicates that salmon stocks have declined strongly (>75%) in Normandy as a whole, which was corroborated by a similarly large decline in salmon catches at Montchaton (a 99% decline in numbers caught in the 14th–15th century)^10^. Therefore, we considered the price-based data from this earliest time period to be reliable and representative, and have therefore included them in our analysis.

Archaeological data describing sites with salmon and pike remains were retrieved from several archaeozoological databases [the Dutch archaeozoological database BoneInfo (last accessed on March 4 2014: www.archeologieinnederland.nl, the Belgian Bioarchaeological Inventory (2014) (last accessed on March 4 2014): species.be/archeo/nl/, and the French database: Inventaire National du Patrimoine Naturel (last accessed on March 4 2014): inpn.mnhn.fr/espece/cd_nom/recherche)]; see Supplementary information Table S1 for the combined dataset. Pike was chosen as a references species because of its comparable size and recorded food preference, but different habitat preference which is not dependent on running waters. In addition, watermill construction leads to a shift in habitat suitability from salmon to limnophilic species, making the pike an especially appropriate comparison species. The linkage of socio-economic life styles (early farmers and late farmers) to archaeological periods is based on the recently revised Dutch Cultural Heritage Agency system^52^. Spatial data for present-day salmon distribution in England were obtained from the National Biodiversity Network’s Gateway [UK distribution of Atlantic salmon (last accessed 14 May 2014): data.nbn.org.uk]. Data for watermill construction for the Netherlands, Belgium and Germany were extracted from the mills database (see extended data Tables S1 and S2 for a summary of this data; see Supplementary information Table S2 for the combined dataset). Extensive sections of the Rhine and Meuse catchment located in these countries are addressed in this database. In all, we were able to identify the century in which 1603 out of a total of 2708 watermills present in the database were constructed, covering a period of approximately 13 centuries. Of these, 954 and 649 mills were located in the catchments of the rivers Rhine and Meuse, respectively. If the century of construction could not be determined with 100% certainty, we applied a conservative dating approach which involved choosing the most recent century as the period of construction.

### Data processing

Data for changes in the price of salmon (Fig. 1A,B) were corrected for deflation/inflation by calculating average price increases for other protein-rich food from the same period and area (for Normandy: mutton, pork and partridge were used; for Cologne: several species of fish were used). Subsequently changes in the price of salmon were corrected for by applying this proxy factor. In general, before the 16^th^ century monetary depreciation was hardly an issue in Europe^53^. A comparison of the price of salmon to the relatively stable price for mutton, reveals that salmon prices would have increased by a factor 9.6, which corresponds to a decrease in salmon stocks by nearly 90%. This percentage decline is supported by the changes in salmon catches that occurred at the medieval fishery at Montchaton, France, where annual catches of 300–350 per year in the 14^th^ century declined dramatically to 3 or 4 fish annually by the end of the 15^th^ century^10^. We corrected the price increase for salmon using a measure for deflation derived from mutton, pork and partridge. This yielded a corrected relative price for salmon that increased by a factor of 4.5, which – conservatively – indicates a decrease of more than 75% abundance over 150 years (Table S1). We similarly calculated the price increase of salmon in Cologne by taking only fresh salmon (“Krimpsalm”) into consideration and not dried, smoked or salted salmon. Salmon prices in Scotland were already corrected by Gemmill and Mayhew^15^. The amount of taxes levied in the Netherlands on salmon fisheries during the period 1650–1800 AD was split into two periods. The first ranging from 1650–1747, was characterized by a time when the levying of taxes was leased out, the second extending from 1750 onwards was characterized by a time when taxes were levied directly by the central authorities, viz. without the interposition of leaseholders as middlemen. Two separate periods were applied in the analysis because the changes to the tax system coincided with a major increase in tax revenue that occurred after 1750^50^. A possible explanation for this increase is that salmon fishermen and fish mongers committed large scale fraud in the period 1650–1747, selling salmon illegally in large quantities on the black market and not via official fish auctions. This was certainly the case in the 16^th^ and later centuries^54^ which suggests that a certain amount of high value fish catches was not traded via official pathways in the second half of the 16^th^ century. Apparently, salmon had become such a valuable commodity that it even provoked illegal trade. After taking over responsibility for tax levies in 1750, the central authorities succeeded in eliminating this fraudulent activity, where the leaseholders previously failed. Consequently, more salmon was traded via the official pathways, resulting in increased tax revenue. By indexing the first decade of direct tax levy (1750–1759) to the same level as the last decade of the indirect tax levy (1740–1749), the rate of taxation as an index of salmon abundance becomes consistent. This consistent reduction in tax incomes reflects a 70% reduction in salmon catches across the whole time period.

All time series were indexed by setting the data from the first year at 100 and recalculating subsequent years relative to this adjusted figure. For abundances calculated from salmon prices data (Fig. 1A,B,F), reverse indices were calculated that assumed a linear relationship between increasing prices and declining stocks. The separate time series were linked using extrapolation. A hyperbolic function was fitted to each time series to prevent negative salmon indices, ensuring that this is a conservative extrapolation. The linked time series for salmon decline and watermill construction were then approximated with a polynomial function which explains 99.0% of the variation in watermill construction and >97.5% of the variation in salmon decline. For the period 1260–1940 AD, we used the aforementioned polynomials to generate 69 data points containing information on both salmon decline and watermill construction employing time steps of 10 years. This procedure circumvents the mismatch in resolution that occurs between time series in the original data (e.g., watermill presence data was per 100 years, but salmon data resolution varied from years to decades to centuries), thus allowing the direct calculation of relationship between salmon decline and watermill construction. In addition to the aforementioned 69 data points, we generated similar time series where we either conservatively assumed that salmon stocks were constant for periods with no data, or omitted the first time series from lower Normandy (1260–1410 AD). This methodology led to 40 data points for the period 1550–1940 AD. Regression analyses were performed for each of these three sets of generated data points, where salmon decline was modelled as a function of watermill presence. In each of the three analyses, there was a negative, highly significant correlation between salmon stock development and watermill development (Period 1260–1940: declining stocks for periods without data: r = −0.813 and P < 0.0001; Period 1260–1940: constant stocks for periods without data: r = −0.828 and P < 0.0001; Period 1550–1940: declining stocks for periods without data: r = −0.903 and P < 0.0001).

The number of sites with salmon remains was compared to the number of sites with pike remains for six distinct socio-economic/historical periods: I. Early farmers (Late Middle Neolithic-Early Middle Bronze Age [3400 BC–1500 BC]); II. Late farmers (Late Middle Bronze Age-Roman Period [1500 BC–450 AD]); III. Early Middle Ages [450–900 AD]; IV. High Middle Ages [900–1250 AD]; V. Late Middle Ages [1250–1500 AD]; VI. Modern Period [1500 AD-present]. The demarcation of periods is based on distinctive socio-economic changes^52^. A comparison of sites with salmon remains to sites with pike remains was carried out in order to correct for unevenly distributed numbers of excavations over the periods distinguished. Assuming stable stocks of both species, one would expect an increase in the salmon-pike ratio since salmon bones are more susceptible to decay (especially in well-aerated conditions) than pike bones^55^.

## Additional Information

**How to cite this article**: Lenders, H. J. R. *et al*. Historical rise of waterpower initiated the collapse of salmon stocks. *Sci. Rep.* **6**, 29269; doi: 10.1038/srep29269 (2016).

## Supplementary Material

Supplementary Information

## Figures and Tables

**Table 1:** Changes in the ratio of the number of archaeological sites in the Netherlands, Belgium and Northern France with salmon and pike remains (N_salmon_/N_pike_) in six time periods (Early farmers: Late Middle Neolithic-Early Middle Bronze Age; Late farmers: Late Middle Bronze Age-Roman Period; Early Middle Ages (450–900 AD); High Middle Ages (900–1250 AD); Late Middle Ages (1250–1500 AD); Modern Times (1500 AD- Present).

Time period	Number of sites		N_salmon_/N_pike_	∆ N_salmon_/N_pike_	Interpretation
	*Salmo salar*	*Esox lucius*			
Early farmers (Late Middle Neolithic-Early Middle Bronze Age)	3	29	0.10	–	–
Late farmers (Late Middle Bronze Age-Roman Period)	16	73	0.22	+0.12	Salmon stocks constant; the increasing ratio results from salmon remains decaying more rapidly than pike remains.
Early Middle Ages (450–900 AD)	5	12	0.42	+0.20	
High Middle Ages (900–1250 AD)	3	13	0.23	−0.19	Salmon stocks decline strongly due to the impact of watermills
Late Middle Ages (1250–1500 AD)	7	44	0.16	−0.07	
Modern Times (1500 AD- Present)	21	77	0.27	+0.11	Salmon import results in an increased ratio

Note that constant stocks are expected to result in an increasing ratio as salmon remains decay more rapidly than pike remains. Therefore, the sharp fall in the salmon-pike ratio indicates a strong decline of Atlantic salmon starting from the transition of the Early to the High Middle Ages. The last column gives an interpretation of the observed ratio change.
