# Supplementary material for: Historical rise of waterpower initiated the collapse of salmon stocks
Source: Sci Rep. 2016 Jul 20;6:29269. doi: 10.1038/srep29269 (PMC4951639; doi:10.1038/srep29269)
Supplement: Supplementary Information [file srep29269-s1.doc]

**Supplementary information**

Historical rise of waterpower initiated the collapse of salmon stocks

H.J.R. Lenders^a^, T.P.M. Chamuleau^a,b^, A.J. Hendriks^a^, R.C.G.M. Lauwerier^c^, R.S.E.W. Leuven^a^ & W.C.E.P.Verberk^d^.

^a^ Institute for Water and Wetland Research, Department of Environmental Science, Radboud University, P.O. Box 9010, 6500 GL Nijmegen, The Netherlands.

^b^ Rijkswaterstaat, Centre for Water, Traffic and Environment, P.O. Box 17, 8200 AA Lelystad, The Netherlands.

^c^ Department of Archaeology, Cultural Heritage Agency, Ministry of Education, Culture and Science, P.O. Box 1600, 3800 BP Amersfoort, The Netherlands.

^d^ Institute for Water and Wetland Research, Department of Animal Ecology and Physiology, Radboud University, P.O. Box 9010, 6500 GL Nijmegen, The Netherlands.

**Extended data Table 1. The** number of watermills first mentioned per century and cumulative number of watermills over the centuries for the Dutch, Belgian and German parts of the Rhine and Meuse catchments.

|  | Number of mills | | | | | | | |
| --- | --- | --- | --- | --- | --- | --- | --- | --- |
|  | Overshot | | Undershot | | Other/ unknown | | Total | |
| Century | First mentioned | Cumulative | First mentioned | Cumulative | First mentioned | Cumulative | First mentioned | Cumulative |
| 8 | 0 | 0 | 1 | 1 | 1 | 1 | 2 | 2 |
| 9 | 1 | 1 | 1 | 2 | 1 | 2 | 3 | 5 |
| 10 | 1 | 2 | 2 | 4 | 2 | 4 | 5 | 10 |
| 11 | 6 | 8 | 2 | 6 | 8 | 12 | 16 | 26 |
| 12 | 13 | 21 | 8 | 14 | 19 | 31 | 40 | 66 |
| 13 | 20 | 41 | 33 | 47 | 54 | 85 | 107 | 173 |
| 14 | 31 | 72 | 48 | 95 | 84 | 169 | 163 | 336 |
| 15 | 45 | 117 | 38 | 133 | 88 | 257 | 171 | 507 |
| 16 | 61 | 178 | 45 | 178 | 79 | 336 | 185 | 692 |
| 17 | 81 | 259 | 52 | 230 | 111 | 447 | 244 | 936 |
| 18 | 140 | 399 | 111 | 341 | 111 | 558 | 362 | 1298 |
| 19 | 124 | 523 | 54 | 395 | 93 | 651 | 271 | 1569 |
| 20 | 19 | 542 | 2 | 397 | 13 | 664 | 34 | 1603 |

Extended data Table 2. **The total number of watermills recorded per catchment and type and per catchment/country and type.**

| Total | Catchment | No | Type | No | % | Country | No | Type | No | % |
| --- | --- | --- | --- | --- | --- | --- | --- | --- | --- | --- |
|  |  |  |  |  |  |  |  |  |  |  |
| 2707 | Rhine | 1995 | Overshot | 494 | 25 | Netherlands | 144 | Overshot | 35 | 24 |
|  |  |  | Breastshot | 71 | 4 |  |  | Breastshot | 0 | 0 |
|  |  |  | Undershot | 292 | 15 |  |  | Undershot | 28 | 19 |
|  |  |  | Other/ unknown | 1138 | 57 |  |  | Other/ unknown | 81 | 56 |
|  |  |  |  |  |  |  |  |  |  |  |
|  |  |  |  |  |  | Germany | 1851 | Overshot | 459 | 25 |
|  |  |  |  |  |  |  |  | Breastshot | 71 | 4 |
|  |  |  |  |  |  |  |  | Undershot | 264 | 14 |
|  |  |  |  |  |  |  |  | Other/ unknown | 1057 | 57 |
|  |  |  |  |  |  |  |  |  |  |  |
|  | Meuse | 712 | Overshot | 278 | 39 | Netherlands | 174 | Overshot | 24 | 14 |
|  |  |  | Breastshot | 51 | 7 |  |  | Breastshot | 31 | 18 |
|  |  |  | Undershot | 245 | 34 |  |  | Undershot | 62 | 36 |
|  |  |  | Other/ unknown | 138 | 19 |  |  | Other/ unknown | 57 | 33 |
|  |  |  |  |  |  |  |  |  |  |  |
|  |  |  |  |  |  | Belgium | 538 | Overshot | 254 | 47 |
|  |  |  |  |  |  |  |  | Breastshot | 20 | 4 |
|  |  |  |  |  |  |  |  | Undershot | 183 | 34 |
|  |  |  |  |  |  |  |  | Other/ unknown | 81 | 15 |

Table S1. **Archaeological sites with remains of Atlantic salmon (*S. salar*) and/or Northern pike (*E. lucius*) in the Netherlands (Nl), Belgium (B) and France (Fr) with the socio-economic period designated.**

| Species | Country | Location | Latitude | Longitude | Socio-economic period |
| --- | --- | --- | --- | --- | --- |
| *S. salar* | Nl | Schokland | 52.6488 | 5.7800 | Early farmers |
| *S. salar* | Nl | Aartswoud | 52.7460 | 4.9666 | Early farmers |
| *S. salar* | Nl | Schokland | 52.6488 | 5.7800 | Early farmers |
| *S. salar* | B | Wange | 50.7856 | 5.0322 | Later farmers |
| *S. salar* | Fr | Boulogne-sur-Mer | 50.7264 | 1.6147 | Later farmers |
| *S. salar* | Fr | Fontenay-en-Parisis | 49.4122 | 2.4277 | Later farmers |
| *S. salar* | Nl | Vlaardingen | 51.9077 | 4.3263 | Later farmers |
| *S. salar* | Nl | Nijmegen | 51.8306 | 5.8805 | Later farmers |
| *S. salar* | Nl | Valkenburg | 52.1794 | 4.4331 | Later farmers |
| *S. salar* | Nl | Valkenburg | 52.1794 | 4.4331 | Later farmers |
| *S. salar* | Nl | Nijmegen | 51.8306 | 5.8805 | Later farmers |
| *S. salar* | Nl | Valkenburg | 52.1794 | 4.4331 | Later farmers |
| *S. salar* | Nl | Voorburg | 52.0679 | 4.3643 | Later farmers |
| *S. salar* | Nl | Den Haag | 52.0986 | 4.2824 | Later farmers |
| *S. salar* | Nl | Valkenburg | 52.1794 | 4.4331 | Later farmers |
| *S. salar* | Nl | Velsen | 52.4512 | 4.6732 | Later farmers |
| *S. salar* | Nl | Velsen | 52.4512 | 4.6732 | Later farmers |
| *S. salar* | Nl | Valkenburg | 52.1794 | 4.4331 | Later farmers |
| *S. salar* | Nl | Leiden | 52.1497 | 4.5178 | Later farmers |
| *S. salar* | B | Huy | 50.5219 | 5.2350 | Early Middle Ages |
| *S. salar* | B | Huy | 50.5219 | 5.2350 | Early Middle Ages |
| *S. salar* | Nl | Den Haag | 52.0987 | 4.2793 | Early Middle Ages |
| *S. salar* | Nl | Wijk bij Duurstede | 51.9772 | 5.3470 | Early Middle Ages |
| *S. salar* | Nl | Deventer | 52.2508 | 6.1581 | Early Middle Ages |
| *S. salar* | Nl | Zutphen | 52.1246 | 6.2225 | High Middle Ages |
| *S. salar* | Nl | Zutphen | 52.1398 | 6.1943 | High Middle Ages |
| *S. salar* | Nl | Deventer | 52.2508 | 6.1581 | High Middle Ages |
| *S. salar* | B | Antwerp | 51.2213 | 4.3972 | Late Middle Ages |
| *S. salar* | B | Ename | 50.8550 | 3.6328 | Late Middle Ages |
| *S. salar* | B | Mechelen | 51.0278 | 4.4811 | Late Middle Ages |
| *S. salar* | B | Mechelen | 51.0278 | 4.4811 | Late Middle Ages |
| *S. salar* | Nl | Leiden | 52.1592 | 4.4908 | Late Middle Ages |
| *S. salar* | Nl | Rotterdam | 51.9180 | 4.4860 | Late Middle Ages |
| *S. salar* | Nl | Veere | 51.5493 | 3.6683 | Late Middle Ages |
| *S. salar* | B | Antwerp | 51.2213 | 4.3972 | Modern Times |
| *S. salar* | B | Antwerp | 51.2213 | 4.3972 | Modern Times |
| *S. salar* | B | Bruges | 51.2094 | 3.2252 | Modern Times |
| *S. salar* | B | Dendermonde | 51.0300 | 4.0989 | Modern Times |
| *S. salar* | B | Ename | 50.8550 | 3.6328 | Modern Times |
| *S. salar* | B | Kortrijk | 50.8281 | 3.2650 | Modern Times |
| *S. salar* | B | Mechelen | 51.0278 | 4.4811 | Modern Times |
| *S. salar* | B | Mons | 50.4533 | 3.9486 | Modern Times |
| *S. salar* | B | Mons | 50.4533 | 3.9486 | Modern Times |
| *S. salar* | Fr | Brie-Comte-Robert | 48.6917 | 2.6083 | Modern Times |
| *S. salar* | Fr | Paris | 48.8566 | 2.3518 | Modern Times |
| *S. salar* | Fr | Compiègne | 49.4149 | 2.8231 | Modern Times |
| *S. salar* | Fr | Amiens | 49.8920 | 2.2990 | Modern Times |
| *S. salar* | Nl | Gouda | 52.0104 | 4.7032 | Modern Times |
| *S. salar* | Nl | Rotterdam | 51.9180 | 4.4860 | Modern Times |
| *S. salar* | Nl | Breda | 51.5783 | 4.7669 | Modern Times |
| *S. salar* | Nl | Dordrecht | 51.8122 | 4.6647 | Modern Times |
| *S. salar* | Nl | Venlo | 51.3616 | 6.1625 | Modern Times |
| *S. salar* | Nl | Alkmaar | 52.6297 | 4.7373 | Modern Times |
| *S. salar* | Nl | Zutphen | 52.1397 | 6.1941 | Modern Times |
| *S. salar* | Nl | Amsterdam | 52.3851 | 4.8834 | Modern Times |
| *E. lucius* | Nl | Lienden | 51.9205 | 5.5569 | Early farmers |
| *E. lucius* | Nl | Meteren | 51.8593 | 5.2588 | Early farmers |
| *E. lucius* | Nl | Schagen | 52.7910 | 4.8065 | Early farmers |
| *E. lucius* | Nl | Barendrecht | 51.8554 | 4.5308 | Early farmers |
| *E. lucius* | Nl | Kesteren | 51.9201 | 5.5599 | Early farmers |
| *E. lucius* | Nl | Schokland | 52.6488 | 5.7800 | Early farmers |
| *E. lucius* | Nl | Emmeloord | 52.6660 | 5.7405 | Early farmers |
| *E. lucius* | Nl | Meteren | 51.8593 | 5.2611 | Early farmers |
| *E. lucius* | Nl | Voorschoten | 52.1325 | 4.4393 | Early farmers |
| *E. lucius* | Nl | Albrandswaard | 51.8371 | 4.4876 | Early farmers |
| *E. lucius* | Nl | Albrandswaard | 51.8454 | 4.4004 | Early farmers |
| *E. lucius* | Nl | Barendrecht | 51.8554 | 4.5308 | Early farmers |
| *E. lucius* | Nl | Barendrecht | 51.8554 | 4.5308 | Early farmers |
| *E. lucius* | Nl | Aartswoud | 52.7460 | 4.9666 | Early farmers |
| *E. lucius* | Nl | Barendrecht | 51.8554 | 4.5308 | Early farmers |
| *E. lucius* | Nl | Hoogwoud | 52.7345 | 4.9362 | Early farmers |
| *E. lucius* | Nl | Aartswoud | 52.7462 | 4.9764 | Early farmers |
| *E. lucius* | Nl | Aartswoud | 52.7463 | 4.9823 | Early farmers |
| *E. lucius* | Nl | Aartswoud | 52.7492 | 4.9647 | Early farmers |
| *E. lucius* | Nl | Sijbekarspel | 52.7127 | 4.9822 | Early farmers |
| *E. lucius* | Nl | Hekelingen | 51.8246 | 4.3313 | Early farmers |
| *E. lucius* | Nl | Winkel | 52.7639 | 4.9376 | Early farmers |
| *E. lucius* | Nl | Barendrecht | 51.8554 | 4.5308 | Early farmers |
| *E. lucius* | Nl | Barendrecht | 51.8554 | 4.5308 | Early farmers |
| *E. lucius* | Nl | Schokland | 52.6488 | 5.7800 | Early farmers |
| *E. lucius* | Nl | Emmeloord | 52.6660 | 5.7405 | Early farmers |
| *E. lucius* | Nl | Hekelingen | 51.8246 | 4.3313 | Early farmers |
| *E. lucius* | Nl | Vlaardingen | 51.9045 | 4.3175 | Early farmers |
| *E. lucius* | Nl | Spijkenisse | 51.8333 | 4.3167 | Early farmers |
| *E. lucius* | B | Tournai | 50.6058 | 3.3883 | Later farmers |
| *E. lucius* | B | Kortrijk | 50.8281 | 3.2650 | Later farmers |
| *E. lucius* | B | Namur | 50.4642 | 4.8608 | Later farmers |
| *E. lucius* | B | Oudenburg | 51.1844 | 3.0047 | Later farmers |
| *E. lucius* | B | Tongeren | 50.7808 | 5.4647 | Later farmers |
| *E. lucius* | B | Liberchies | 50.5142 | 4.4217 | Later farmers |
| *E. lucius* | B | Tournai | 50.6058 | 3.3883 | Later farmers |
| *E. lucius* | B | Tournai | 50.6058 | 3.3883 | Later farmers |
| *E. lucius* | Fr | Grisy-sur-Seine | 48.4392 | 3.3175 | Later farmers |
| *E. lucius* | Fr | Lagny-sur-Marne | 48.8788 | 2.7075 | Later farmers |
| *E. lucius* | Fr | Bobigny | 48.9106 | 2.4397 | Later farmers |
| *E. lucius* | Fr | Acy-Romance | 49.5019 | 4.3422 | Later farmers |
| *E. lucius* | Fr | Biesheim | 48.0417 | 7.5439 | Later farmers |
| *E. lucius* | Fr | Châteaubleau | 48.5886 | 3.1083 | Later farmers |
| *E. lucius* | Fr | Meaux | 48.9603 | 2.8883 | Later farmers |
| *E. lucius* | Fr | Meaux | 48.9603 | 2.8883 | Later farmers |
| *E. lucius* | Fr | Paris | 48.8566 | 2.3518 | Later farmers |
| *E. lucius* | Fr | Paris | 48.8566 | 2.3518 | Later farmers |
| *E. lucius* | Fr | Paris | 48.8566 | 2.3518 | Later farmers |
| *E. lucius* | Fr | Bennecourt | 49.0408 | 1.5631 | Later farmers |
| *E. lucius* | Fr | Paris | 48.8566 | 2.3518 | Later farmers |
| *E. lucius* | Nl | Dodewaard | 51.9215 | 5.6477 | Later farmers |
| *E. lucius* | Nl | Houten | 52.0270 | 5.1560 | Later farmers |
| *E. lucius* | Nl | Tiel | 51.8675 | 5.4039 | Later farmers |
| *E. lucius* | Nl | Meteren | 51.8594 | 5.2892 | Later farmers |
| *E. lucius* | Nl | Leiden | 52.1505 | 4.4492 | Later farmers |
| *E. lucius* | Nl | Vlaardingen | 51.9443 | 4.3434 | Later farmers |
| *E. lucius* | Nl | Midden Delfland | 51.9360 | 4.3020 | Later farmers |
| *E. lucius* | Nl | Kesteren | 51.9200 | 5.5653 | Later farmers |
| *E. lucius* | Nl | Westmaas | 51.7912 | 4.4971 | Later farmers |
| *E. lucius* | Nl | Breukelerwaard | 52.1725 | 4.9858 | Later farmers |
| *E. lucius* | Nl | Schiedam | 51.9380 | 4.4027 | Later farmers |
| *E. lucius* | Nl | Houten | 52.0270 | 5.1560 | Later farmers |
| *E. lucius* | Nl | Vleuten de Meern | 52.0852 | 5.0761 | Later farmers |
| *E. lucius* | Nl | Wijk bij Duurstede | 51.9772 | 5.3470 | Later farmers |
| *E. lucius* | Nl | Tiel | 51.8675 | 5.4039 | Later farmers |
| *E. lucius* | Nl | Nijmegen | 51.8468 | 5.8697 | Later farmers |
| *E. lucius* | Nl | Vlaardingen | 51.9077 | 4.3263 | Later farmers |
| *E. lucius* | Nl | Wijk bij Duurstede | 51.9736 | 5.3304 | Later farmers |
| *E. lucius* | Nl | Vleuten | 52.0839 | 5.0091 | Later farmers |
| *E. lucius* | Nl | Nijmegen | 51.8306 | 5.8805 | Later farmers |
| *E. lucius* | Nl | Valkenburg | 52.1794 | 4.4331 | Later farmers |
| *E. lucius* | Nl | Nijmegen | 51.8306 | 5.8805 | Later farmers |
| *E. lucius* | Nl | Kesteren | 51.9200 | 5.5653 | Later farmers |
| *E. lucius* | Nl | Utrecht | 52.0827 | 5.0079 | Later farmers |
| *E. lucius* | Nl | Woerden | 52.0852 | 4.8827 | Later farmers |
| *E. lucius* | Nl | Kesteren | 51.9200 | 5.5653 | Later farmers |
| *E. lucius* | Nl | Burgersdijk | 51.9665 | 4.2726 | Later farmers |
| *E. lucius* | Nl | Valkenburg | 52.1794 | 4.4331 | Later farmers |
| *E. lucius* | Nl | Den Haag | 52.0828 | 4.2091 | Later farmers |
| *E. lucius* | Nl | Voorburg | 52.0679 | 4.3643 | Later farmers |
| *E. lucius* | Nl | De Meern | 52.0809 | 5.0204 | Later farmers |
| *E. lucius* | Nl | Valkenburg | 52.1794 | 4.4331 | Later farmers |
| *E. lucius* | Nl | Castricum | 52.5427 | 4.6764 | Later farmers |
| *E. lucius* | Nl | Kesteren | 51.9200 | 5.5653 | Later farmers |
| *E. lucius* | Nl | Utrecht | 52.0827 | 5.0079 | Later farmers |
| *E. lucius* | Nl | Leidsche Rijn | 52.0960 | 5.0662 | Later farmers |
| *E. lucius* | Nl | Nijmegen | 51.8381 | 5.8943 | Later farmers |
| *E. lucius* | Nl | Velsen | 52.4512 | 4.6732 | Later farmers |
| *E. lucius* | Nl | Assendelft | 52.4937 | 4.7367 | Later farmers |
| *E. lucius* | Nl | Elden | 51.9717 | 5.8733 | Later farmers |
| *E. lucius* | Nl | Velsen | 52.4539 | 4.6731 | Later farmers |
| *E. lucius* | Nl | Nijmegen | 51.8450 | 5.8752 | Later farmers |
| *E. lucius* | Nl | Velsen | 52.4563 | 4.6594 | Later farmers |
| *E. lucius* | Nl | Valkenburg | 52.1794 | 4.4331 | Later farmers |
| *E. lucius* | Nl | Woerden | 52.0858 | 4.8824 | Later farmers |
| *E. lucius* | Nl | Kesteren | 51.9200 | 5.5653 | Later farmers |
| *E. lucius* | Nl | Bodegraven | 52.0814 | 4.7307 | Later farmers |
| *E. lucius* | Nl | Zaltbommel | 51.8167 | 5.2500 | Later farmers |
| *E. lucius* | Nl | Leiden | 52.1497 | 4.5178 | Later farmers |
| *E. lucius* | Nl | Woerden | 52.0853 | 4.8838 | Later farmers |
| *E. lucius* | Nl | Utrecht | 52.0827 | 5.0079 | Later farmers |
| *E. lucius* | Nl | Oostbuurt | 51.9762 | 4.2786 | Later farmers |
| *E. lucius* | B | Namur | 50.4642 | 4.8608 | Early Middle Ages |
| *E. lucius* | Nl | Den Haag | 52.0987 | 4.2793 | Early Middle Ages |
| *E. lucius* | Nl | Odijk | 52.0538 | 5.2276 | Early Middle Ages |
| *E. lucius* | Nl | Vleuten-De Meern | 52.0852 | 5.0761 | Early Middle Ages |
| *E. lucius* | Nl | Vleuten-De Meern | 52.0852 | 5.0761 | Early Middle Ages |
| *E. lucius* | Nl | Leiderdorp | 52.1534 | 4.5315 | Early Middle Ages |
| *E. lucius* | Nl | Leiden | 52.1497 | 4.5178 | Early Middle Ages |
| *E. lucius* | Nl | Castricum | 52.5427 | 4.6764 | Early Middle Ages |
| *E. lucius* | Nl | Vleuten-De Meern | 52.0852 | 5.0761 | Early Middle Ages |
| *E. lucius* | Nl | Wijk bij Duurstede | 51.8873 | 5.3471 | Early Middle Ages |
| *E. lucius* | Nl | Buren | 51.8983 | 5.3984 | Early Middle Ages |
| *E. lucius* | Nl | Deventer | 52.2508 | 6.1581 | Early Middle Ages |
| *E. lucius* | B | Dendermonde | 51.0300 | 4.0989 | High Middle Ages |
| *E. lucius* | B | Aalst | 50.9375 | 4.0411 | High Middle Ages |
| *E. lucius* | Fr | Boves | 49.8461 | 2.3917 | High Middle Ages |
| *E. lucius* | Fr | Roissy-en-France | 49.0050 | 2.5189 | High Middle Ages |
| *E. lucius* | Nl | Houten | 52.0204 | 5.1856 | High Middle Ages |
| *E. lucius* | Nl | Kerk-Avezaath | 51.8936 | 5.3854 | High Middle Ages |
| *E. lucius* | Nl | Rotterdam | 51.9180 | 4.4860 | High Middle Ages |
| *E. lucius* | Nl | Buren | 51.8983 | 5.3984 | High Middle Ages |
| *E. lucius* | Nl | Zutphen | 52.1246 | 6.2225 | High Middle Ages |
| *E. lucius* | Nl | Kerk-Avezaath | 51.8936 | 5.3854 | High Middle Ages |
| *E. lucius* | Nl | Buren | 51.8983 | 5.3984 | High Middle Ages |
| *E. lucius* | Nl | Zutphen | 52.1398 | 6.1943 | High Middle Ages |
| *E. lucius* | Nl | Deventer | 52.2508 | 6.1581 | High Middle Ages |
| *E. lucius* | B | Antwerp | 51.2213 | 4.3972 | Late Middle Ages |
| *E. lucius* | B | Antwerp | 51.2213 | 4.3972 | Late Middle Ages |
| *E. lucius* | B | Bruges | 51.2094 | 3.2252 | Late Middle Ages |
| *E. lucius* | B | Bruxelles | 50.8467 | 4.3547 | Late Middle Ages |
| *E. lucius* | B | Bruxelles | 50.8467 | 4.3547 | Late Middle Ages |
| *E. lucius* | B | Bruxelles | 50.8467 | 4.3547 | Late Middle Ages |
| *E. lucius* | B | Ename | 50.8550 | 3.6328 | Late Middle Ages |
| *E. lucius* | B | Gent | 51.0539 | 3.7050 | Late Middle Ages |
| *E. lucius* | B | Kortrijk | 50.8281 | 3.2650 | Late Middle Ages |
| *E. lucius* | B | Laarne | 51.0294 | 3.8500 | Late Middle Ages |
| *E. lucius* | B | Londerzeel | 51.0017 | 4.3022 | Late Middle Ages |
| *E. lucius* | B | Mechelen | 51.0278 | 4.4811 | Late Middle Ages |
| *E. lucius* | B | Mechelen | 51.0278 | 4.4811 | Late Middle Ages |
| *E. lucius* | B | Raversijde | 51.2061 | 2.8639 | Late Middle Ages |
| *E. lucius* | B | Sinaai | 51.1572 | 4.0428 | Late Middle Ages |
| *E. lucius* | Fr | Lille | 50.6372 | 3.0633 | Late Middle Ages |
| *E. lucius* | Fr | L´Étoile | 50.0244 | 2.0328 | Late Middle Ages |
| *E. lucius* | Fr | Amiens | 49.8920 | 2.2990 | Late Middle Ages |
| *E. lucius* | Fr | Bourg-la-Reine | 48.7796 | 2.3151 | Late Middle Ages |
| *E. lucius* | Fr | Paris | 48.8566 | 2.3518 | Late Middle Ages |
| *E. lucius* | Nl | Zweins | 53.1885 | 5.5967 | Late Middle Ages |
| *E. lucius* | Nl | Kerk-Avezaath | 51.8936 | 5.3854 | Late Middle Ages |
| *E. lucius* | Nl | Roermond | 51.1936 | 5.9983 | Late Middle Ages |
| *E. lucius* | Nl | Middelburg | 51.5003 | 3.6147 | Late Middle Ages |
| *E. lucius* | Nl | Amsterdam | 52.3746 | 4.8945 | Late Middle Ages |
| *E. lucius* | Nl | Hoorn | 52.6396 | 5.0589 | Late Middle Ages |
| *E. lucius* | Nl | Leiden | 52.1592 | 4.4908 | Late Middle Ages |
| *E. lucius* | Nl | Dordrecht | 51.8122 | 4.6647 | Late Middle Ages |
| *E. lucius* | Nl | Gorinchem | 51.8294 | 4.9720 | Late Middle Ages |
| *E. lucius* | Nl | Gouda | 52.0104 | 4.7032 | Late Middle Ages |
| *E. lucius* | Nl | Bunschoten | 52.2425 | 5.3742 | Late Middle Ages |
| *E. lucius* | Nl | Eindhoven | 51.4388 | 5.4803 | Late Middle Ages |
| *E. lucius* | Nl | Amersfoort | 52.1552 | 5.3872 | Late Middle Ages |
| *E. lucius* | Nl | Alkmaar | 52.6297 | 4.7373 | Late Middle Ages |
| *E. lucius* | Nl | Amsterdam | 52.3710 | 4.8935 | Late Middle Ages |
| *E. lucius* | Nl | Gouda | 52.0104 | 4.7032 | Late Middle Ages |
| *E. lucius* | Nl | Delft | 51.9977 | 4.3388 | Late Middle Ages |
| *E. lucius* | Nl | Haarlem | 52.3775 | 4.6382 | Late Middle Ages |
| *E. lucius* | Nl | Alkmaar | 52.6297 | 4.7373 | Late Middle Ages |
| *E. lucius* | Nl | Amsterdam | 52.3731 | 4.8951 | Late Middle Ages |
| *E. lucius* | Nl | Breda | 51.5873 | 4.7667 | Late Middle Ages |
| *E. lucius* | Nl | Dordrecht | 51.8122 | 4.6647 | Late Middle Ages |
| *E. lucius* | Nl | Deventer | 52.2508 | 6.1581 | Late Middle Ages |
| *E. lucius* | Nl | Culemborg | 51.9483 | 5.2345 | Late Middle Ages |
| *E. lucius* | B | Antwerp | 51.2213 | 4.3972 | Modern Times |
| *E. lucius* | B | Antwerp | 51.2213 | 4.3972 | Modern Times |
| *E. lucius* | B | Bruxelles | 50.8467 | 4.3547 | Modern Times |
| *E. lucius* | B | Dendermonde | 51.0300 | 4.0989 | Modern Times |
| *E. lucius* | B | Ename | 50.8550 | 3.6328 | Modern Times |
| *E. lucius* | B | Kortrijk | 50.8281 | 3.2650 | Modern Times |
| *E. lucius* | B | Kortrijk | 50.8281 | 3.2650 | Modern Times |
| *E. lucius* | B | Leuven | 50.8775 | 4.7044 | Modern Times |
| *E. lucius* | B | Londerzeel | 51.0017 | 4.3022 | Modern Times |
| *E. lucius* | B | Maaseik | 51.0947 | 5.8106 | Modern Times |
| *E. lucius* | B | Mechelen | 51.0278 | 4.4811 | Modern Times |
| *E. lucius* | B | Mons | 50.4533 | 3.9486 | Modern Times |
| *E. lucius* | B | Bruges | 51.2094 | 3.2252 | Modern Times |
| *E. lucius* | B | Rochefort | 50.1617 | 5.2222 | Modern Times |
| *E. lucius* | B | Antwerp | 51.2213 | 4.3972 | Modern Times |
| *E. lucius* | B | Antwerp | 51.2213 | 4.3972 | Modern Times |
| *E. lucius* | B | Bruxelles | 50.8467 | 4.3547 | Modern Times |
| *E. lucius* | B | Ename | 50.8550 | 3.6328 | Modern Times |
| *E. lucius* | B | Gent | 51.0539 | 3.7050 | Modern Times |
| *E. lucius* | B | Maaseik | 51.0947 | 5.8106 | Modern Times |
| *E. lucius* | B | Namur | 50.4642 | 4.8608 | Modern Times |
| *E. lucius* | B | Tongeren | 50.7808 | 5.4647 | Modern Times |
| *E. lucius* | B | Bruxelles | 50.8467 | 4.3547 | Modern Times |
| *E. lucius* | B | Mons | 50.4533 | 3.9486 | Modern Times |
| *E. lucius* | Fr | Amiens | 49.8920 | 2.2990 | Modern Times |
| *E. lucius* | Fr | Beauvais | 49.4303 | 2.0952 | Modern Times |
| *E. lucius* | Fr | Compiègne | 49.4149 | 2.8231 | Modern Times |
| *E. lucius* | Fr | Nanteuil-le-Haudouin | 49.1425 | 2.8114 | Modern Times |
| *E. lucius* | Fr | Reims | 49.2628 | 4.0347 | Modern Times |
| *E. lucius* | Fr | Roissy-en-France | 49.0050 | 2.5189 | Modern Times |
| *E. lucius* | Nl | Gouda | 52.0104 | 4.7032 | Modern Times |
| *E. lucius* | Nl | Kerk-Avezaath | 51.8936 | 5.3854 | Modern Times |
| *E. lucius* | Nl | Venlo | 51.3616 | 6.1625 | Modern Times |
| *E. lucius* | Nl | Rijswijk | 52.0425 | 4.3231 | Modern Times |
| *E. lucius* | Nl | Sassenheim | 52.2239 | 4.5237 | Modern Times |
| *E. lucius* | Nl | Delft | 52.0068 | 4.3531 | Modern Times |
| *E. lucius* | Nl | Den Bosch | 51.6877 | 5.3006 | Modern Times |
| *E. lucius* | Nl | Zwolle | 52.5214 | 6.1239 | Modern Times |
| *E. lucius* | Nl | Rotterdam | 51.9180 | 4.4860 | Modern Times |
| *E. lucius* | Nl | Gorinchem | 51.8294 | 4.9720 | Modern Times |
| *E. lucius* | Nl | Rotterdam | 51.9180 | 4.4860 | Modern Times |
| *E. lucius* | Nl | Haarlem | 52.3811 | 4.6367 | Modern Times |
| *E. lucius* | Nl | Zwolle | 52.5126 | 6.0943 | Modern Times |
| *E. lucius* | Nl | Breda | 51.5783 | 4.7669 | Modern Times |
| *E. lucius* | Nl | Dordrecht | 51.8122 | 4.6647 | Modern Times |
| *E. lucius* | Nl | Groningen | 53.2188 | 6.5699 | Modern Times |
| *E. lucius* | Nl | Haarlem | 52.3775 | 4.6382 | Modern Times |
| *E. lucius* | Nl | Alkmaar | 52.6297 | 4.7373 | Modern Times |
| *E. lucius* | Nl | Utrecht | 52.0827 | 5.0079 | Modern Times |
| *E. lucius* | Nl | Arnhem | 51.9476 | 5.8381 | Modern Times |
| *E. lucius* | Nl | Zeewolde | 52.3258 | 5.5339 | Modern Times |
| *E. lucius* | Nl | Dordrecht | 51.8122 | 4.6647 | Modern Times |
| *E. lucius* | Nl | Gorinchem | 51.8219 | 4.9666 | Modern Times |
| *E. lucius* | Nl | Venlo | 51.3616 | 6.1625 | Modern Times |
| *E. lucius* | Nl | Alkmaar | 52.6297 | 4.7373 | Modern Times |
| *E. lucius* | Nl | Kampen | 52.5494 | 5.9180 | Modern Times |
| *E. lucius* | Nl | Zwolle | 52.5137 | 6.0899 | Modern Times |
| *E. lucius* | Nl | Groningen | 53.2189 | 6.5550 | Modern Times |
| *E. lucius* | Nl | Gorinchem | 51.8294 | 4.9720 | Modern Times |
| *E. lucius* | Nl | Hoorn | 52.6396 | 5.0589 | Modern Times |
| *E. lucius* | Nl | Deventer | 52.2508 | 6.1581 | Modern Times |
| *E. lucius* | Nl | Zutphen | 52.1397 | 6.1941 | Modern Times |
| *E. lucius* | Nl | Dwingeloo | 52.7534 | 6.3500 | Modern Times |
| *E. lucius* | Nl | Venlo | 51.3616 | 6.1625 | Modern Times |
| *E. lucius* | Nl | Harlingen | 53.1746 | 5.4186 | Modern Times |
| *E. lucius* | Nl | Den Haag | 52.0783 | 4.3077 | Modern Times |
| *E. lucius* | Nl | Amsterdam | 52.3851 | 4.8834 | Modern Times |
| *E. lucius* | Nl | Tilburg | 51.5600 | 5.1150 | Modern Times |
| *E. lucius* | Nl | Haarlem | 52.3820 | 4.6380 | Modern Times |
| *E. lucius* | Nl | Buren | 51.8983 | 5.3984 | Modern Times |
| *E. lucius* | Nl | Bourtange | 53.0070 | 7.1913 | Modern Times |
| *E. lucius* | Nl | Vleuten | 52.1016 | 5.0209 | Modern Times |
| *E. lucius* | Nl | Deventer | 52.2508 | 6.1581 | Modern Times |
| *E. lucius* | Nl | Vlaardingen | 51.9078 | 4.3409 | Modern Times |
| *E. lucius* | Nl | Den Haag | 52.0783 | 4.3077 | Modern Times |
| *E. lucius* | Nl | Tiel | 51.8864 | 5.4360 | Modern Times |
| *E. lucius* | Nl | Zeelst | 51.4181 | 5.4160 | Modern Times |

Table S2. **Century in which** **watermills are first recorded in the Dutch, Belgian and German parts of the Rhine and Meuse catchments.**

| Century | Mill type | Mill name | River / brook | River catchment | Country |
| --- | --- | --- | --- | --- | --- |
| 8 | Undershot | Meiersmuhle/Avenstrotsmuhle |  | Rhine | Germany |
| 8 | Unknown | Kolb'sche Mühle |  | Rhine | Germany |
| 9 | Undershot | De watermolen, Reppelmolen, Cuppensmolen |  | Meuse | Belgium |
| 9 | Overshot | Broicher Mühle | Villiper Bach | Rhine | Germany |
| 9 | Unknown | Wassermuhle GroB Schonebeck |  | Rhine | Germany |
| 10 | Overshot | Geyener Muhle | Pulheimer Bach | Rhine | Germany |
| 10 | Undershot | Plagemanns Muhle | Vechte | Rhine | Germany |
| 10 | Undershot | Wartenberger Mühle |  | Rhine | Germany |
| 10 | Unknown | Würmmühle | Würm | Rhine | Germany |
| 10 | Unknown |  | Voorste Diep | Rhine | Netherlands |
| 11 | Overshot | Moulin Dutilleux | Waya | Meuse | Belgium |
| 11 | Overshot | Moulin du parc |  | Meuse | Belgium |
| 11 | Overshot | Moulin de Ferrieres | Heredia | Meuse | Belgium |
| 11 | Overshot | Moulin de Lomprez | Ry d-Ave | Meuse | Belgium |
| 11 | Undershot | Pollismolen | Itterbeek | Meuse | Belgium |
| 11 | Breastshot | Wassermuhle Overmeyer | Halverder Aa | Rhine | Germany |
| 11 | Overshot | Untere Bibermühle |  | Rhine | Germany |
| 11 | Overshot | Herrenmühle | Herrenbach | Rhine | Germany |
| 11 | Undershot | Tannenmühle |  | Rhine | Germany |
| 11 | Unknown | Alt-Enginger Muhle/Altenginger Muhle |  | Rhine | Germany |
| 11 | Unknown | Bienwald Mühle |  | Rhine | Germany |
| 11 | Unknown | Eckl Mühle |  | Rhine | Germany |
| 11 | Unknown | Grafmühle |  | Rhine | Germany |
| 11 | Unknown | Reubelsmühle |  | Rhine | Germany |
| 11 | Unknown | Untere Mühle |  | Rhine | Germany |
| 11 | Unknown |  | Uffelterstroom | Rhine | Netherlands |
| 12 | Overshot | Molen van Halmaal | Molenbeek | Meuse | Belgium |
| 12 | Overshot | Moulin de Chevlipont | Dijle | Meuse | Belgium |
| 12 | Overshot | Moulin de Saint-Pont | Hain | Meuse | Belgium |
| 12 | Overshot | Moulin Meix | Chevralle | Meuse | Belgium |
| 12 | Overshot | Moulin a Baudet |  | Meuse | Belgium |
| 12 | Overshot | Moulin de beausart, Moulin de la Chapelle | Pietrebais | Meuse | Belgium |
| 12 | Overshot | Moulin (du Fayai) | Warchenne | Meuse | Belgium |
| 12 | Overshot | Moulin de la Ferme de la Paix Dieu |  | Meuse | Belgium |
| 12 | Overshot | Moulin de l-Abbaye |  | Meuse | Belgium |
| 12 | Turbine | Moulin de Bilhee | Dender | Meuse | Belgium |
| 12 | Turbine | De Keyart, Keyaertmolen |  | Meuse | Belgium |
| 12 | Turbine | Moulin de Petit-Rosiere | Grote Gete | Meuse | Belgium |
| 12 | Turbine | Moulin de maffle |  | Meuse | Belgium |
| 12 | Turbine | Moulin d-ogy | salpot | Meuse | Belgium |
| 12 | Undershot | Kluismolen / Molen van Mariendaal / Joostenmolen | Aabeek | Meuse | Belgium |
| 12 | Undershot | Moulin brasserie de I |  | Meuse | Belgium |
| 12 | Breastshot | SchloBmuhle | Steinfurter Aa | Rhine | Germany |
| 12 | Overshot | Heesfelder Kornmuhle |  | Rhine | Germany |
| 12 | Overshot | Wassermuhle Nenkersdorf |  | Rhine | Germany |
| 12 | Overshot | Mühle vor dem Obertor |  | Rhine | Germany |
| 12 | Overshot | Wassermühle Birgel |  | Rhine | Germany |
| 12 | Undershot | Oebelsmuhle | Rothbach | Rhine | Germany |
| 12 | Undershot | Ophovener Muhle | Baalbach | Rhine | Germany |
| 12 | Undershot | Obermühle |  | Rhine | Germany |
| 12 | Unknown | GroBe Teichsmuhle | Heubach | Rhine | Germany |
| 12 | Unknown | Wilde(n)rather Muhle | Niers | Rhine | Germany |
| 12 | Unknown | Stadtmühle |  | Rhine | Germany |
| 12 | Unknown | Ebersbergmühle |  | Rhine | Germany |
| 12 | Unknown | Stadtmühle |  | Rhine | Germany |
| 12 | Unknown | Mittelmühle |  | Rhine | Germany |
| 12 | Unknown |  | Dinkel | Rhine | Germany |
| 12 | Unknown | Steinsmuhle | Niers | Rhine | Germany |
| 12 | Unknown | Aldenhover Muhle/Liecker Muhle | Liecker Bach (Junge Wurm) | Rhine | Germany |
| 12 | Unknown | Schloß Mühle | Fränkische Saale | Rhine | Germany |
| 12 | Unknown | Wolfsfurter Muhlen | Wurm | Rhine | Germany |
| 12 | Unknown | Burgmühle |  | Rhine | Germany |
| 12 | Unknown | Ginghartinger Mühle |  | Rhine | Germany |
| 12 | Undershot | De Watermolen | Molenbeek | Meuse | Netherlands |
| 12 | Undershot | Kasterense Watermolen |  | Meuse | Netherlands |
| 12 | Undershot | Den Haller | Molenbeek | Rhine | Netherlands |
| 13 | Breastshot | Vieux Moulin, Moulin du Chateau, Moulin banal | le Hain | Meuse | Belgium |
| 13 | Breastshot | Aldeneikermolen, Audenijmolen | Bosbeek | Meuse | Belgium |
| 13 | Breastshot | Metsterenmolen | Melsterbeek | Meuse | Belgium |
| 13 | Overshot | Moulin du Rieu | Rieu | Meuse | Belgium |
| 13 | Overshot | Moulin Banal/Vieux Moulin | Ohain | Meuse | Belgium |
| 13 | Overshot | Genaderse Molen / Molen van Tenhaagdoorn | Laambeek | Meuse | Belgium |
| 13 | Overshot | Moulin de Flavion |  | Meuse | Belgium |
| 13 | Overshot | Moulin d-Aulnoit, Moulin Frison |  | Meuse | Belgium |
| 13 | Overshot | Moulin Bouckaert |  | Meuse | Belgium |
| 13 | Overshot | Moulin de Maret, Moulin Verbiest | Kleine Gete | Meuse | Belgium |
| 13 | Overshot | Moulin de Genville | Genville, Gobertange | Meuse | Belgium |
| 13 | Overshot | Moulin de jauche-le-male | Kliene Gete | Meuse | Belgium |
| 13 | Overshot | Moulin de Henry Fontaine, moulin Choisy, Neuf Moulin | Henri Fontaine | Meuse | Belgium |
| 13 | Overshot | Moulin de l'Arton | Harton | Meuse | Belgium |
| 13 | Overshot | Moulin de Dourbes, Grand Moulin | Viroin | Meuse | Belgium |
| 13 | Overshot | Moulin de l'Abbaye, Moulin stordoir | Samson | Meuse | Belgium |
| 13 | Overshot | Moulin d-Hollers | Thyle | Meuse | Belgium |
| 13 | Overshot | Moulin de l-Abbaye d-Aulne |  | Meuse | Belgium |
| 13 | Turbine | Moulin de Ripain | Zenne | Meuse | Belgium |
| 13 | Turbine | Moulin des Pres |  | Meuse | Belgium |
| 13 | Turbine | Grevenmolen | Molenbeek | Meuse | Belgium |
| 13 | Turbine | Moulin de la rue des Trois Ruelles | Dender | Meuse | Belgium |
| 13 | Turbine | Moulin du Marais des Soeurs, Moulin Vindevoghel |  | Meuse | Belgium |
| 13 | Turbine | Moulin de Orp-le-Grand, Moulin Bernar | Kleine Gete | Meuse | Belgium |
| 13 | Turbine | Moulin de la Biesmelle |  | Meuse | Belgium |
| 13 | Turbine | Van Veldemolen, Velkermolen | Demer | Meuse | Belgium |
| 13 | Turbine | Moulin Jorissen | Geer (Jeker) | Meuse | Belgium |
| 13 | Turbine | Grand Moulin, Moulin des Rendages | Grote Gete | Meuse | Belgium |
| 13 | Turbine | Moulin de Geest | Grote Gete | Meuse | Belgium |
| 13 | Undershot | Hornemolen, Guvelingenmolen, Molen van Meesbroek | Melsterbeek | Meuse | Belgium |
| 13 | Undershot | Tuiltermolen, Tuldermolen | Tuilterdemmer | Meuse | Belgium |
| 13 | Undershot | Slagmolen | Dommel | Meuse | Belgium |
| 13 | Undershot | Kleine Molen, Volmolen | Dommel | Meuse | Belgium |
| 13 | Undershot | Herkenrodemolen | Demer | Meuse | Belgium |
| 13 | Undershot | Blaarmolen | Jeker | Meuse | Belgium |
| 13 | Undershot | Graatmolen | Herk | Meuse | Belgium |
| 13 | Undershot | Elsartmolen, Elshoutmolen | Herk | Meuse | Belgium |
| 13 | Undershot | Luimertingenmolen | Mombeek / Winterbeek | Meuse | Belgium |
| 13 | Undershot | Daalmolen | Jeker | Meuse | Belgium |
| 13 | Undershot | Wedelse Molen |  | Meuse | Belgium |
| 13 | Undershot | Bemvoortse molen | Dommel | Meuse | Belgium |
| 13 | Undershot | Moulin du Tordoir | Tordoir | Meuse | Belgium |
| 13 | Undershot | Moulin du Grognard, moulin Flamand | Grote Gete | Meuse | Belgium |
| 13 | Undershot | Ghen-Aa-molen | Abeek | Meuse | Belgium |
| 13 | Undershot | Rullingenmolen, Nieuwe Molen | Herk | Meuse | Belgium |
| 13 | Undershot | Althoeseltse molen, Oude Molen | Demer | Meuse | Belgium |
| 13 | Undershot | Moulin de l-Abbaye de La Ramee | Gete | Meuse | Belgium |
| 13 | Undershot | Moulin Thully, Moulin Petite Dendre | Petite Dendre | Meuse | Belgium |
| 13 | Breastshot | Dalheimer Muhle | Rothenbach | Rhine | Germany |
| 13 | Breastshot | Strauss Mühle |  | Rhine | Germany |
| 13 | Overshot | Bischofsmuhle | Honigbach | Rhine | Germany |
| 13 | Overshot | Alte muhle von Loe |  | Rhine | Germany |
| 13 | Overshot | Gehringsmühle |  | Rhine | Germany |
| 13 | Overshot | Reibeisen Mühle |  | Rhine | Germany |
| 13 | Undershot | Burgmuhle |  | Rhine | Germany |
| 13 | Undershot | havixbeck-Hohenholte | Munsterischen Aa | Rhine | Germany |
| 13 | Undershot | Obermühle |  | Rhine | Germany |
| 13 | Undershot | Zievericher Muhle | Erft | Rhine | Germany |
| 13 | Undershot | Bongartz Muhle | Hammerbach | Rhine | Germany |
| 13 | Undershot | Bettinger Mühle |  | Rhine | Germany |
| 13 | Undershot | Klostermuhle Gravenhorst |  | Rhine | Germany |
| 13 | Undershot | Bliesheimer Muhle | Liblarer muhlengraben | Rhine | Germany |
| 13 | Undershot | Kalkumer Muhle | Schwarzbach | Rhine | Germany |
| 13 | Undershot | Theisenmühle |  | Rhine | Germany |
| 13 | Undershot | Benninghofmuhle | Rehrbach | Rhine | Germany |
| 13 | Unknown | Trappen Muhle | Geseker bach | Rhine | Germany |
| 13 | Unknown | Klostermühle | Kiedrichbach | Rhine | Germany |
| 13 | Unknown | Eppinghovener Muhle | Erft | Rhine | Germany |
| 13 | Unknown | Muhle des Hofes Probsting |  | Rhine | Germany |
| 13 | Unknown | Muhle Christian Weitz | Linnicher Muhlenteich | Rhine | Germany |
| 13 | Unknown | Stadtmuhle |  | Rhine | Germany |
| 13 | Unknown | Reg Mühle |  | Rhine | Germany |
| 13 | Unknown | Seemühle |  | Rhine | Germany |
| 13 | Unknown | erprather Muhle | Erft | Rhine | Germany |
| 13 | Unknown | Rimburger Muhle | Wurm | Rhine | Germany |
| 13 | Unknown | Horster Muhle | Ruhr | Rhine | Germany |
| 13 | Unknown | Muhle Kottmann | erft | Rhine | Germany |
| 13 | Unknown | Sudmuhle | Werse | Rhine | Germany |
| 13 | Unknown | SchloBmuhle | Niers | Rhine | Germany |
| 13 | Unknown | Berwicker Muhle |  | Rhine | Germany |
| 13 | Unknown | hanschen Muhle | Geseker bach | Rhine | Germany |
| 13 | Unknown | Pepermuhle |  | Rhine | Germany |
| 13 | Unknown | Ölmühle Niederdorfelden | Nidder | Rhine | Germany |
| 13 | Unknown | Leitzachmühle |  | Rhine | Germany |
| 13 | Unknown | Mühle beim Hinterbauernhof |  | Rhine | Germany |
| 13 | Unknown | Erftmuhle | Erft | Rhine | Germany |
| 13 | Unknown | Oedter Muhle | Niers | Rhine | Germany |
| 13 | Unknown | Getreidemühle |  | Rhine | Germany |
| 13 | Unknown | Ailinger Mühle |  | Rhine | Germany |
| 13 | Unknown | Ziegelsmühle |  | Rhine | Germany |
| 13 | Unknown | Pfaffenmuhle Einbrungen | Schwarzbach | Rhine | Germany |
| 13 | Unknown | Herrnmühle |  | Rhine | Germany |
| 13 | Unknown | Die Öle |  | Rhine | Germany |
| 13 | Unknown | Viller Muhle | Niers | Rhine | Germany |
| 13 | Unknown | Klippertzmuhle | Niers | Rhine | Germany |
| 13 | Unknown | Engelsmuhle | Rodebach | Rhine | Germany |
| 13 | Unknown | Schlechtsmühle | Reichenbach | Rhine | Germany |
| 13 | Unknown | Maier Mühle |  | Rhine | Germany |
| 13 | Breastshot | Sint-Elizabethsmolen | Leubeek | Meuse | Netherlands |
| 13 | Undershot | Venbergse Watermolen | Dommel | Meuse | Netherlands |
| 13 | Unknown | Onderste Molen / Commandeursmolen | Geul | Meuse | Netherlands |
| 13 | Unknown | Molen van Cardus Houden | Geleenbeek | Meuse | Netherlands |
| 13 | Overshot | Gasthuismolen | Beekbergense- of Oude Beek | Rhine | Netherlands |
| 13 | Undershot | Geesterensche Watermolen / Nordmeule / Nadermolen / Naerremolen | Molenbeek | Rhine | Netherlands |
| 13 | Undershot |  | Berkel | Rhine | Netherlands |
| 13 | Unknown |  | Deurzerdiep | Rhine | Netherlands |
| 13 | Unknown |  | Deurzerdiep | Rhine | Netherlands |
| 14 | Overshot | Moulin Saint-Catherin |  | Meuse | Belgium |
| 14 | Overshot | Moulin Hick, Moulin du Valdieau | Berwinne | Meuse | Belgium |
| 14 | Overshot | Moulin de Froyennes | Ruisseau Saint Eloi | Meuse | Belgium |
| 14 | Overshot | Moulin de Hubinne | Bocq | Meuse | Belgium |
| 14 | Overshot | Moulin Del Val | Ri Ternel | Meuse | Belgium |
| 14 | Overshot | Grand Moulin, Moulin Lallemand | Ry d-Oneux | Meuse | Belgium |
| 14 | Overshot | Vieux Moulin, Moulin du Fief | Senette | Meuse | Belgium |
| 14 | Overshot | Moulin de la Haze |  | Meuse | Belgium |
| 14 | Overshot | Moulin de Vellereille |  | Meuse | Belgium |
| 14 | Overshot | Moulin de Scoville | Bocq | Meuse | Belgium |
| 14 | Overshot | Moulin de Cortil | Orne | Meuse | Belgium |
| 14 | Turbine | Neermolen | Bosbeek | Meuse | Belgium |
| 14 | Turbine | Moulin du Piroir, Moulin de Biez | Train | Meuse | Belgium |
| 14 | Turbine | Moulin Brule |  | Meuse | Belgium |
| 14 | Turbine | Moulin du Village |  | Meuse | Belgium |
| 14 | Turbine | Grand Moulin, Moulin Banal | Dijle | Meuse | Belgium |
| 14 | Turbine | Moulin de Lumay | Grote Gete | Meuse | Belgium |
| 14 | Undershot | Bosmolen | Bosbeek | Meuse | Belgium |
| 14 | Undershot | Bombroekmolen | Mombeek | Meuse | Belgium |
| 14 | Undershot | Grevenbroekmolen | Warmbeek | Meuse | Belgium |
| 14 | Undershot | Zelkermolen | Velpe | Meuse | Belgium |
| 14 | Undershot | Herkermolen |  | Meuse | Belgium |
| 14 | Undershot | Ancien moulin communale | Ourthe | Meuse | Belgium |
| 14 | Undershot | Moulin Garenne, Moulin de Marq, Moulin de Coppens | Marcq | Meuse | Belgium |
| 14 | Undershot | Dorpsmolen, Gemeentemolen, Banmolen |  | Meuse | Belgium |
| 14 | Undershot | Stalse Molen, Lemmensmolen | Zwarte Beek en Maalbeek | Meuse | Belgium |
| 14 | Undershot | Moulin de Fallais, ferme Haine | Mehaigne | Meuse | Belgium |
| 14 | Undershot | Kapittel- of Sapitelmolen | Demer | Meuse | Belgium |
| 14 | Undershot | Ruttermolen | Jeker | Meuse | Belgium |
| 14 | Undershot | Oude Molen | Zwarte Beek | Meuse | Belgium |
| 14 | Undershot | Wijkmolen, Nijstenmolen | Jeker | Meuse | Belgium |
| 14 | Undershot | moulin d-Oeil | Sure | Meuse | Belgium |
| 14 | Undershot | Dubbelmolen |  | Meuse | Belgium |
| 14 | Undershot | Elsbroekmolen, Molen van Binderveld | Melsterbeek | Meuse | Belgium |
| 14 | Undershot | Moulin de Bellevaux | Ambleve | Meuse | Belgium |
| 14 | Undershot | Oude Molen | Herk | Meuse | Belgium |
| 14 | Undershot | Rentfortmolen | Demer | Meuse | Belgium |
| 14 | Undershot | Moulin de Thy |  | Meuse | Belgium |
| 14 | Breastshot | Tetzer Mühle | Malefinkbach | Rhine | Germany |
| 14 | Breastshot | Wallhalber Mühle | Arnbach | Rhine | Germany |
| 14 | Breastshot | Niedermuhle | Steinfurter Aa | Rhine | Germany |
| 14 | Breastshot | Wassermühle Hellermann |  | Rhine | Germany |
| 14 | Breastshot | Stadtmühle | Wörnitz | Rhine | Germany |
| 14 | Breastshot | Fessler Mühle | Metter | Rhine | Germany |
| 14 | Overshot | Mittlere Muhle |  | Rhine | Germany |
| 14 | Overshot | Kainsbacher Mühle | Kainsbach | Rhine | Germany |
| 14 | Overshot | Gemündener Mühle | Pützborner Bach | Rhine | Germany |
| 14 | Overshot | Schneide Mühle/ Mühle Meisburg |  | Rhine | Germany |
| 14 | Overshot | Obermuhle | Amstelbach | Rhine | Germany |
| 14 | Overshot | Fretter Mühle |  | Rhine | Germany |
| 14 | Overshot | BerkelMuhle | Berkel | Rhine | Germany |
| 14 | Overshot | Nieborger Wassermuhle | Dinkel | Rhine | Germany |
| 14 | Overshot | Oedmühle |  | Rhine | Germany |
| 14 | Overshot | Kuhle Muhle |  | Rhine | Germany |
| 14 | Overshot | Lehnsmuhle/Gottschalks Muhle | Itterbach/Muhlengraben | Rhine | Germany |
| 14 | Overshot | Völmeder Müller | Völmeder Bach | Rhine | Germany |
| 14 | Overshot | Holzmühle |  | Rhine | Germany |
| 14 | Overshot | Baurnmühle/ Bohrbachsmühle |  | Rhine | Germany |
| 14 | Overshot | Eselmühle |  | Rhine | Germany |
| 14 | Overshot | Gumpertsmühle |  | Rhine | Germany |
| 14 | Undershot | Kornmuhle | Junge Wurm | Rhine | Germany |
| 14 | Undershot | Gymnicher Muhle | Erft | Rhine | Germany |
| 14 | Undershot | Brommler Muhle | Rodebach | Rhine | Germany |
| 14 | Undershot | Radermuhle | Schwalm | Rhine | Germany |
| 14 | Undershot | Dammer Muhle | Dussel | Rhine | Germany |
| 14 | Undershot | Schepers Muhle |  | Rhine | Germany |
| 14 | Undershot | Klostermühle Vinnenberg | Bever | Rhine | Germany |
| 14 | Undershot | Holtmühle | Mühlenbach (Schwalm) | Rhine | Germany |
| 14 | Undershot | Unterste Mühle Holten | Holtener Mühlenbach | Rhine | Germany |
| 14 | Undershot | Wassermühle Haus Caen | Niers | Rhine | Germany |
| 14 | Undershot | Gustorfer Mühle | Erft | Rhine | Germany |
| 14 | Undershot | Dirmerzheimer Mühle | Rothbach | Rhine | Germany |
| 14 | Undershot | Kentener Muhle | Kleine Erft | Rhine | Germany |
| 14 | Undershot | Heerser Muhle |  | Rhine | Germany |
| 14 | Undershot | Dilborner Mühle | Schwalm | Rhine | Germany |
| 14 | Undershot | Kellereimuhle | Angerbach | Rhine | Germany |
| 14 | Undershot | Sindorfer Mühle | Große Erft | Rhine | Germany |
| 14 | Undershot | Leomühle | Schwalm | Rhine | Germany |
| 14 | Undershot | Untermühle |  | Rhine | Germany |
| 14 | Undershot | Fuchsmühle | Tauber | Rhine | Germany |
| 14 | Unknown | Asper Mühle | Niers | Rhine | Germany |
| 14 | Unknown | Fuchtelner Muhle | Stever | Rhine | Germany |
| 14 | Unknown | Untere Mühle | Erft | Rhine | Germany |
| 14 | Unknown | Untere Mühle |  | Rhine | Germany |
| 14 | Unknown | Broicher Mühle | Niers | Rhine | Germany |
| 14 | Unknown | Buscher Mühle | nördliche Düssel | Rhine | Germany |
| 14 | Unknown | Winklermühle |  | Rhine | Germany |
| 14 | Unknown | Havichhorster Mühle | Werse | Rhine | Germany |
| 14 | Unknown | SchloBmuhle | Niers | Rhine | Germany |
| 14 | Unknown | Dammer Mühle | Düssel Aue | Rhine | Germany |
| 14 | Unknown | Schravelener Muhle | Niers | Rhine | Germany |
| 14 | Unknown | Neumühle | Niers | Rhine | Germany |
| 14 | Unknown | Nonnenmuhle | Niers | Rhine | Germany |
| 14 | Unknown | Hoster Muhle | Niers | Rhine | Germany |
| 14 | Unknown | Koßlesmühle/ Vogelsmühle/ Ziegelmühle/ Stoffelsmühle | Holzbach | Rhine | Germany |
| 14 | Unknown | Schleimer Mühle | Ruhr | Rhine | Germany |
| 14 | Unknown | Obere Kapfenhardter Mühle |  | Rhine | Germany |
| 14 | Unknown | Raußmühle |  | Rhine | Germany |
| 14 | Unknown | Paffendorfer Mühle | Erft | Rhine | Germany |
| 14 | Unknown | Stiftsmühle |  | Rhine | Germany |
| 14 | Unknown | Wassermühle Haus Kakesbeck | Stever | Rhine | Germany |
| 14 | Unknown | Holzmühle |  | Rhine | Germany |
| 14 | Unknown | Oberste Muhle | Linnicher Muhlenteich | Rhine | Germany |
| 14 | Unknown | Unterste Muhle | Linnicher Muhlenteich | Rhine | Germany |
| 14 | Unknown | Klostermuhle Mariental | Issel | Rhine | Germany |
| 14 | Unknown | Neersdommer Muhle | Niers | Rhine | Germany |
| 14 | Unknown | Bauermühle |  | Rhine | Germany |
| 14 | Unknown | Kovermühle | Nette | Rhine | Germany |
| 14 | Unknown | Pfaffenhofener Mühle |  | Rhine | Germany |
| 14 | Unknown | Olmuhle Haus Engelrading |  | Rhine | Germany |
| 14 | Unknown | Pliesmuhle | kleine Erft | Rhine | Germany |
| 14 | Unknown | Honselaersmühle | Issumer Fleuth | Rhine | Germany |
| 14 | Unknown | Lamberrtsmuhle | Wiembach | Rhine | Germany |
| 14 | Unknown |  | Rodebach | Rhine | Germany |
| 14 | Unknown | Isenbrucher Mühle | Rodebach | Rhine | Germany |
| 14 | Unknown | Neumühle | Berkel | Rhine | Germany |
| 14 | Unknown | Weuthenmühle/ Boisheimer Mühle | Nette | Rhine | Germany |
| 14 | Unknown | Wichheimer Mühle | Faulbach | Rhine | Germany |
| 14 | Unknown | Wolfhager Mühle | Mühlenbach/ Junge Wurm | Rhine | Germany |
| 14 | Unknown | Eberbacher Klostermühle | Kiedrichbach | Rhine | Germany |
| 14 | Unknown | Neue Muhle/Moriansmuhle | Alte Emscher | Rhine | Germany |
| 14 | Unknown | Konigsmuhle | Konigsbach | Rhine | Germany |
| 14 | Unknown | Untere SchloBmuhle | Uneter Schermbecker Muhlenbach | Rhine | Germany |
| 14 | Unknown | Maysack'sche Mühle |  | Rhine | Germany |
| 14 | Unknown | Saxler Mühle | Alf | Rhine | Germany |
| 14 | Unknown | Neumuhle | Nette | Rhine | Germany |
| 14 | Unknown | SchloBmuhle Trips | Wurm | Rhine | Germany |
| 14 | Unknown | Hünhover Wassermühle | Kleiner Wurm | Rhine | Germany |
| 14 | Unknown | Bremstalmühle |  | Rhine | Germany |
| 14 | Unknown | Coenen Muhle |  | Rhine | Germany |
| 14 | Unknown | Mäulesmühle | Reichenbach | Rhine | Germany |
| 14 | Unknown | Obere Mühle | Reichenbach | Rhine | Germany |
| 14 | Unknown | Kasterer Mühle | Kasterer Mühlenerft | Rhine | Germany |
| 14 | Unknown | Clorather Muhle | Niers | Rhine | Germany |
| 14 | Unknown | Hammermühle | Otterbach | Rhine | Germany |
| 14 | Unknown | Bibersfelder Mühle | Bibers | Rhine | Germany |
| 14 | Unknown | Gräbelesmühle | Leubas | Rhine | Germany |
| 14 | Unknown | Hammermuhle |  | Rhine | Germany |
| 14 | Unknown | Bolensmühle/ Eschermanns Mühle/ Hamanns Mühle |  | Rhine | Germany |
| 14 | Unknown | Obere Eselsmühle |  | Rhine | Germany |
| 14 | Unknown | Hummelmühle |  | Rhine | Germany |
| 14 | Unknown | Brandhöfer Mühl |  | Rhine | Germany |
| 14 | Unknown | Effelter Mühle | Doberbach | Rhine | Germany |
| 14 | Breastshot | Slottermolen | Everlose Beek | Meuse | Netherlands |
| 14 | Overshot | Sint Jansmolen | Spaubeek | Meuse | Netherlands |
| 14 | Overshot | De Broekmolen / De Drakenmolen | Molenbeek | Meuse | Netherlands |
| 14 | Undershot | Molen van De Visscher | Leij | Meuse | Netherlands |
| 14 | Undershot | Dommelse Watermolen | Dommel | Meuse | Netherlands |
| 14 | Undershot | Stevertse Watermolen | Run | Meuse | Netherlands |
| 14 | Unknown | De Borchmolen | Dommel | Meuse | Netherlands |
| 14 | Overshot | De Wenumse Molen | Wenumse Beek | Rhine | Netherlands |
| 14 | Overshot | waterrad molen bij kasteel Vorden | de Vordense beek | Rhine | Netherlands |
| 14 | Undershot | Den Helder / Plekenpolsemolen |  | Rhine | Netherlands |
| 14 | Undershot | De Verdermolen / De Vermolen |  | Rhine | Netherlands |
| 14 | Undershot | De Noordmolen | Oelerbeek/Azelerbeek | Rhine | Netherlands |
| 14 | Undershot | Dickninger Meule | Reest | Rhine | Netherlands |
| 14 | Unknown | Chritiaan Geurtmolen / Werklust |  | Rhine | Netherlands |
| 14 | Unknown |  | Loodiep | Rhine | Netherlands |
| 14 | Unknown |  | Loodiep | Rhine | Netherlands |
| 14 | Unknown |  | Grote Diep, Oostervoortse Diep | Rhine | Netherlands |
| 14 | Unknown |  | Peizerdiep | Rhine | Netherlands |
| 14 | Unknown |  | Beilerstroom | Rhine | Netherlands |
| 14 | Unknown |  | Oudemolensche Diep | Rhine | Netherlands |
| 15 | Breastshot | Moulin d-Arenberg | Zenne | Meuse | Belgium |
| 15 | Breastshot | Moulin de Litrange, Moulin de la Forge, Moulin de Fer | Nethen | Meuse | Belgium |
| 15 | Overshot | Moulin de la Forge | Neufmoulin | Meuse | Belgium |
| 15 | Overshot | Moulin de Hollange | Strange | Meuse | Belgium |
| 15 | Overshot | Moulin a Rie, Mouin Frison | Rieu Bouillant | Meuse | Belgium |
| 15 | Overshot | Moulin Rassart | Ri du Fontaine | Meuse | Belgium |
| 15 | Overshot | Moulin Lietens |  | Meuse | Belgium |
| 15 | Overshot | Moulin Thorez, moulin du Sart, moulin Detrooz | Hoegne | Meuse | Belgium |
| 15 | Overshot | Moulin du Bloquia | Le Train | Meuse | Belgium |
| 15 | Overshot | Kikmolen |  | Meuse | Belgium |
| 15 | Overshot | Moulin Humblet | Fond de Harze | Meuse | Belgium |
| 15 | Overshot | Oude Molen / Theunissenmolen | Bosbeek | Meuse | Belgium |
| 15 | Overshot | Moulin d-en Haut, moulin de Toulintaut | Leupont | Meuse | Belgium |
| 15 | Overshot | Moulin d-Orchimont | Vresse | Meuse | Belgium |
| 15 | Overshot | Moulin de la Motte, Moulin de la Ferme Bordeaux | Cala | Meuse | Belgium |
| 15 | Overshot | Moulin de Bornival |  | Meuse | Belgium |
| 15 | Overshot | Moulin de Donstiennes | Mondiau | Meuse | Belgium |
| 15 | Turbine | Moulin de Jauche, m. du Syndicat, moulin Paheau | Kleine Gete | Meuse | Belgium |
| 15 | Turbine | Moulin Vroman, Moulin de Bierges | Dijle | Meuse | Belgium |
| 15 | Undershot | Molen van Strabroek, Mulkensmolen | Laambeek | Meuse | Belgium |
| 15 | Undershot | Moulin de Valduc | nethen | Meuse | Belgium |
| 15 | Undershot | Binkenmolen | Abeek | Meuse | Belgium |
| 15 | Undershot | Moulin Navarre |  | Meuse | Belgium |
| 15 | Undershot | Borghmolen | Itterbeek | Meuse | Belgium |
| 15 | Undershot | Neermolen | Abeek | Meuse | Belgium |
| 15 | Undershot | Moulin du Fayt | Grote Gete | Meuse | Belgium |
| 15 | Undershot | Klootsmolen, Damburgmolen | Abeek | Meuse | Belgium |
| 15 | Undershot | Hoogmolen |  | Meuse | Belgium |
| 15 | Undershot | Moulin Lannois, moulin Dessart | Mehaigne | Meuse | Belgium |
| 15 | Undershot | Moulin du Val de Mehaigne, Moulin Grosjean | Mehaigne | Meuse | Belgium |
| 15 | Undershot | Moulin de kevret |  | Meuse | Belgium |
| 15 | Undershot | Moulin Ceulemans | Thyle/Orne | Meuse | Belgium |
| 15 | Breastshot | Borgmuhle | Stever | Rhine | Germany |
| 15 | Breastshot | Obermühle Kerpen | Neffelbach | Rhine | Germany |
| 15 | Breastshot | Mühlrather Mühle | Schwalm | Rhine | Germany |
| 15 | Breastshot | Ulstermühle | Ulster | Rhine | Germany |
| 15 | Breastshot | Untere Mühle | Fulda | Rhine | Germany |
| 15 | Breastshot | Geiserschmiede |  | Rhine | Germany |
| 15 | Breastshot | Schimmelmühle |  | Rhine | Germany |
| 15 | Breastshot | Muckenmühle |  | Rhine | Germany |
| 15 | Overshot | Angermühle |  | Rhine | Germany |
| 15 | Overshot | Wassermühle Marl |  | Rhine | Germany |
| 15 | Overshot | Linkens Muhle/Rote Muhle | Broicher Bach | Rhine | Germany |
| 15 | Overshot | Untere Mühle | Steinbach | Rhine | Germany |
| 15 | Overshot | Welsche Mühle | Haarbach | Rhine | Germany |
| 15 | Overshot | Hagenmühle |  | Rhine | Germany |
| 15 | Overshot | Rothaumühle |  | Rhine | Germany |
| 15 | Overshot | SchloBmuhle | Ellbach | Rhine | Germany |
| 15 | Overshot | Mönchhof Sägmühl |  | Rhine | Germany |
| 15 | Overshot | Essenthoer Mühle |  | Rhine | Germany |
| 15 | Overshot | Herrenmühle | Urselbach | Rhine | Germany |
| 15 | Overshot | Goldberger Muhle |  | Rhine | Germany |
| 15 | Overshot | Hofmühle |  | Rhine | Germany |
| 15 | Overshot | Kinzweiler Muhle | Merzbach | Rhine | Germany |
| 15 | Overshot | Brucker Muhle/Pletschmuhle | Golkrather Bach | Rhine | Germany |
| 15 | Overshot | Heinlesmühle |  | Rhine | Germany |
| 15 | Overshot | Kohlsäge Mühle |  | Rhine | Germany |
| 15 | Overshot | Dieksmuhle/Thomasmuhle | Golkrather Bach | Rhine | Germany |
| 15 | Overshot | Jannings Muhle | Leerbach | Rhine | Germany |
| 15 | Overshot | Pletschmuhle | Pulheimer Bach | Rhine | Germany |
| 15 | Overshot | Hackenberger Mühle |  | Rhine | Germany |
| 15 | Overshot | Knopper Mühle | Stuhlbach | Rhine | Germany |
| 15 | Overshot | Dockweiler Mühle | Rusbach | Rhine | Germany |
| 15 | Overshot | Scherzenmühle | Warmen Steinach | Rhine | Germany |
| 15 | Undershot | Alte Mühle | Kirchdorfer Mühlenbach | Rhine | Germany |
| 15 | Undershot | Borner Muhle | Schwalm | Rhine | Germany |
| 15 | Undershot | Mittelmuhle/Wackersmuhle | Baalbach | Rhine | Germany |
| 15 | Undershot | Lüthen Mühle | Nette | Rhine | Germany |
| 15 | Undershot | Ruhmühle |  | Rhine | Germany |
| 15 | Undershot | Helpensteiner Muhle | Dickelsbach | Rhine | Germany |
| 15 | Undershot | WeiBe Muhle | Broicher Bach | Rhine | Germany |
| 15 | Undershot | Friesenmühle | Friesenbach | Rhine | Germany |
| 15 | Undershot | Sandmuhle | Angerbach | Rhine | Germany |
| 15 | Undershot | Korrenziger Muhle/Strycks Muhle | Malefink | Rhine | Germany |
| 15 | Undershot | Kellenberger Mühle | Kirchberger Mühlenteich | Rhine | Germany |
| 15 | Undershot | Schloßmühle | Gartroper Mühlenbach | Rhine | Germany |
| 15 | Undershot | Holebachmühle | Efze | Rhine | Germany |
| 15 | Undershot | Adamsmuhle | Wurm | Rhine | Germany |
| 15 | Undershot | Suggerather Muhle | Wurm | Rhine | Germany |
| 15 | Undershot | Lüttelforster Mühle | Schwalm | Rhine | Germany |
| 15 | Undershot | Deilbachhammer | Veener Ley/Winnenthaler Kanal | Rhine | Germany |
| 15 | Undershot | Auermuhle | Angerbach | Rhine | Germany |
| 15 | Undershot | Horremer Mühle | Kleine Erft | Rhine | Germany |
| 15 | Undershot |  |  | Rhine | Germany |
| 15 | Undershot | Olligsmuhle | Pulheimer Bach | Rhine | Germany |
| 15 | Undershot | Alte Mühle | Wied | Rhine | Germany |
| 15 | Undershot | Schloßmühle |  | Rhine | Germany |
| 15 | Undershot | Hartmanns Hammerschmiede | Ostrach | Rhine | Germany |
| 15 | Undershot | Teichmühle |  | Rhine | Germany |
| 15 | Unknown | Rischmuhle | Linnicher Muhlenteich | Rhine | Germany |
| 15 | Unknown | Brückenmühle | Main | Rhine | Germany |
| 15 | Unknown | Öl und Jägtsmühle |  | Rhine | Germany |
| 15 | Unknown | Waagmühle | Wehebach | Rhine | Germany |
| 15 | Unknown | Spitalmühle | Glems | Rhine | Germany |
| 15 | Unknown | Halbach Hammer |  | Rhine | Germany |
| 15 | Unknown | Hagmühle |  | Rhine | Germany |
| 15 | Unknown | Untere Kleinmichelsmühle | Reichenbach | Rhine | Germany |
| 15 | Unknown | Olmuhle Rahm | Rahmer bach | Rhine | Germany |
| 15 | Unknown | Vollmuhle Unterbruch | Alte bach | Rhine | Germany |
| 15 | Unknown | Götzenhainer Mühle |  | Rhine | Germany |
| 15 | Unknown | Zaubermühle | Sulzach | Rhine | Germany |
| 15 | Unknown | Off Mühle |  | Rhine | Germany |
| 15 | Unknown | Glöckle Mühle |  | Rhine | Germany |
| 15 | Unknown | Bliesmühle |  | Rhine | Germany |
| 15 | Unknown | Dampfmühle |  | Rhine | Germany |
| 15 | Unknown | Willik'sche Mühle | Niers | Rhine | Germany |
| 15 | Unknown | Gutsmuhle Mullenark | Lendersdorfer Muhleteich | Rhine | Germany |
| 15 | Unknown | Alte Schloßmühle | Modau | Rhine | Germany |
| 15 | Unknown | Haasenmühle | Nacker Bach | Rhine | Germany |
| 15 | Unknown | SchloBmuhle Wissel | Niers | Rhine | Germany |
| 15 | Unknown | Engelsmuhle | Gladbach | Rhine | Germany |
| 15 | Unknown | Kneispermühle |  | Rhine | Germany |
| 15 | Unknown | Kothmuhle | Nette | Rhine | Germany |
| 15 | Unknown | Mühllehen Mühle |  | Rhine | Germany |
| 15 | Unknown | Muhle Radefeld |  | Rhine | Germany |
| 15 | Unknown | Alte Mühle/ Wollspinnerei | Lieser | Rhine | Germany |
| 15 | Unknown | Vaihinger Mühle | Enz | Rhine | Germany |
| 15 | Unknown | Waldmühle |  | Rhine | Germany |
| 15 | Unknown | Morper Mühle | Düssel | Rhine | Germany |
| 15 | Unknown | Rohrsmühle | Eselsbach | Rhine | Germany |
| 15 | Unknown | Schloßmühle |  | Rhine | Germany |
| 15 | Unknown | Obere Muhle | Swistbach | Rhine | Germany |
| 15 | Unknown | Winkelhauser Olmuhle | Angerbach | Rhine | Germany |
| 15 | Unknown | Zweibrugger Muhle | Wurm | Rhine | Germany |
| 15 | Unknown | Tüschenbroicher Ölmühle | Schwalm | Rhine | Germany |
| 15 | Unknown | Kochenmühle | Reichenbach | Rhine | Germany |
| 15 | Unknown | Schlösslesmühle | Reichenbach | Rhine | Germany |
| 15 | Unknown | Schnapfen Mühle |  | Rhine | Germany |
| 15 | Unknown | Eller Muhle | sudliche Dussel | Rhine | Germany |
| 15 | Unknown | Pützborner Mühle | Pützbornerbach | Rhine | Germany |
| 15 | Unknown | Kemper Muhle | Muhlenbach | Rhine | Germany |
| 15 | Unknown | Pletschmuhle/Werder Muhle | Baalbach | Rhine | Germany |
| 15 | Unknown | Markus Mühle | Eifgenbach | Rhine | Germany |
| 15 | Unknown | Mühle Georghausen | Sülz | Rhine | Germany |
| 15 | Unknown | Rengnathmühle | Weißer Laab | Rhine | Germany |
| 15 | Unknown | Vollmuhle | Muhlenbach | Rhine | Germany |
| 15 | Unknown | Platzmuhle | Rodebach | Rhine | Germany |
| 15 | Unknown | Dahlmuhle | Rodebach | Rhine | Germany |
| 15 | Unknown | Berschweiler Olischmihl |  | Rhine | Germany |
| 15 | Unknown | Schirpenbrucher Mühle | Lochbach | Rhine | Germany |
| 15 | Unknown | Burgmühle |  | Rhine | Germany |
| 15 | Unknown | Reuschenberger Mühle | Wupper | Rhine | Germany |
| 15 | Unknown | Wassermühle Haus Wohnung | Rotbach | Rhine | Germany |
| 15 | Unknown | Obermühle | Pfinz | Rhine | Germany |
| 15 | Unknown | korn Muhle | Wurm | Rhine | Germany |
| 15 | Unknown | Altdorfer Mühle |  | Rhine | Germany |
| 15 | Unknown | Königsmühle | Weinberger Bach | Rhine | Germany |
| 15 | Unknown | Etzenrather Muhle | Rodebach | Rhine | Germany |
| 15 | Unknown | Horster Mühle | Junge Wurm | Rhine | Germany |
| 15 | Unknown | Nelsen Muhle | Nette | Rhine | Germany |
| 15 | Unknown | Mittelmühle |  | Rhine | Germany |
| 15 | Unknown | Vodes Wassermuhle |  | Rhine | Germany |
| 15 | Unknown | Niedermanderscheider Talmühle |  | Rhine | Germany |
| 15 | Unknown | Bärenmühle |  | Rhine | Germany |
| 15 | Unknown | Oberlinspher Mühle |  | Rhine | Germany |
| 15 | Unknown | Felsenmühle |  | Rhine | Germany |
| 15 | Breastshot | Leeuwenmolen | Jeker, Zuidtak | Meuse | Netherlands |
| 15 | Unknown | Watermolen van Geldrop | Kleine Dommel | Meuse | Netherlands |
| 15 | Unknown | Baalsbruggermolen | Worm | Meuse | Netherlands |
| 15 | Unknown | Wateroliemolen | Vlier | Meuse | Netherlands |
| 15 | Overshot | St. Agnieten / Bagijnemolen | Sonsbeek | Rhine | Netherlands |
| 15 | Overshot | De Rozendaalse Korenmolen / Wambeecker molen / Leyermolen |  | Rhine | Netherlands |
| 15 | Overshot | De Mast | Vasserbeek | Rhine | Netherlands |
| 15 | Overshot | De Witte Molen | Sonsbeek | Rhine | Netherlands |
| 15 | Overshot | Molen van Nikksels / Molen van Willems / Molen van Volkers |  | Rhine | Netherlands |
| 15 | Overshot |  |  | Rhine | Netherlands |
| 15 | Unknown |  | Ruiner Aa | Rhine | Netherlands |
| 15 | Unknown |  |  | Rhine | Netherlands |
| 15 | Unknown | De Klencke | Drostendiep | Rhine | Netherlands |
| 15 | Unknown |  | Oude of Nieuwe Runde | Rhine | Netherlands |
| 15 | Unknown |  | Drostendiep | Rhine | Netherlands |
| 16 | Overshot | Grand Moulin, Moulin Burghin |  | Meuse | Belgium |
| 16 | Overshot | Moulin Lezin, Moulin de Villance |  | Meuse | Belgium |
| 16 | Overshot | Moulin de Fayt-le-Franc |  | Meuse | Belgium |
| 16 | Overshot | Moulin de Lembree | Lembree | Meuse | Belgium |
| 16 | Overshot | Moulin d-Odeigne, Moulin Dethise | Aisne | Meuse | Belgium |
| 16 | Overshot | Moulin d-Alvaux, M. Tremouroux, M. Defrenne | Grote Gete | Meuse | Belgium |
| 16 | Overshot | Moulin de Vogenee |  | Meuse | Belgium |
| 16 | Overshot | Moulin de Godeupont, Moulin de Blanmont | Orne | Meuse | Belgium |
| 16 | Overshot | Moulin de Combreuil | Sennette | Meuse | Belgium |
| 16 | Overshot | Moulin Massard, Moulin de Montleban | Ourthe | Meuse | Belgium |
| 16 | Overshot | Moulin Baumans, Moulin Piette | Ri de Dreye | Meuse | Belgium |
| 16 | Overshot | Nerummolen | Molenbeek | Meuse | Belgium |
| 16 | Overshot | Moulin Henrot |  | Meuse | Belgium |
| 16 | Overshot | Moulin d- en Bas, Le Vieux Moulin | Hante | Meuse | Belgium |
| 16 | Overshot | Moulin des Roches | Aisne | Meuse | Belgium |
| 16 | Overshot | Moulin de Romedenne, moulin de Chaweson |  | Meuse | Belgium |
| 16 | Turbine | Moulin d-Alvaux | Orne | Meuse | Belgium |
| 16 | Turbine | Moulin de la Passe toute-Outre |  | Meuse | Belgium |
| 16 | Turbine | Moulin de Ronquieres | Sennette | Meuse | Belgium |
| 16 | Turbine | Moulin de la Platinerie | Scherbach | Meuse | Belgium |
| 16 | Turbine | Moulin d-Oborne |  | Meuse | Belgium |
| 16 | Turbine | Moulin Stockis, moulin Lesire |  | Meuse | Belgium |
| 16 | Undershot | Le Molinay | La Magree | Meuse | Belgium |
| 16 | Undershot | Slagmolen | Bosbeek | Meuse | Belgium |
| 16 | Undershot | Luisenmolen of Vooste Luysmolen | Abeek | Meuse | Belgium |
| 16 | Undershot | Slagmolen | Stiemerbeek | Meuse | Belgium |
| 16 | Undershot | Dorpermolen | Bosbeek | Meuse | Belgium |
| 16 | Undershot | Klaaskensmolen | Bosbeek | Meuse | Belgium |
| 16 | Undershot | Moulin de Jupille | Ourthe | Meuse | Belgium |
| 16 | Undershot | Langerenmolen | Bosbeek | Meuse | Belgium |
| 16 | Undershot | Le Vieux Moulin, Moulin de Lessive, Moulin de Magery | Lesse | Meuse | Belgium |
| 16 | Undershot | Herkermolen | Herk | Meuse | Belgium |
| 16 | Undershot | Wurfelder-, Klooster- of Looimolen | Bosbeek | Meuse | Belgium |
| 16 | Undershot | Bilzermolen | Demer | Meuse | Belgium |
| 16 | Undershot | Moulin Saint-Pierre, Moulin Maes | Ronne | Meuse | Belgium |
| 16 | Undershot | le Vieux Moulin |  | Meuse | Belgium |
| 16 | Undershot | Moulin de Chaupny, le Vieux Moulin |  | Meuse | Belgium |
| 16 | Undershot | Moulin de Noville, Vieux Moulin | Mehaigne | Meuse | Belgium |
| 16 | Undershot | Moulin de mredsous | Molignee | Meuse | Belgium |
| 16 | Undershot | Moulin du Bas-Marteau |  | Meuse | Belgium |
| 16 | Undershot | Moulin de la Hunelle | Hunelle | Meuse | Belgium |
| 16 | Breastshot | Stegmühle | Schwarza | Rhine | Germany |
| 16 | Breastshot | Obermühle | Obere Iller | Rhine | Germany |
| 16 | Breastshot | Gutswassermühle |  | Rhine | Germany |
| 16 | Breastshot | Alte Mühle | Möhne | Rhine | Germany |
| 16 | Breastshot | Schubmühle | Lüder | Rhine | Germany |
| 16 | Overshot | Holzlarer Mühle | Holtorfer Bach | Rhine | Germany |
| 16 | Overshot | Brüggemühle |  | Rhine | Germany |
| 16 | Overshot | Stadtmühle |  | Rhine | Germany |
| 16 | Overshot | Dammühle |  | Rhine | Germany |
| 16 | Overshot | Corves Muhle |  | Rhine | Germany |
| 16 | Overshot | Niemollers Muhle | Lutter | Rhine | Germany |
| 16 | Overshot | Steinmetzmühle | Urselbach | Rhine | Germany |
| 16 | Overshot | Rodgener Muhle | Rothenbach | Rhine | Germany |
| 16 | Overshot | Braunsfelder wassermuhle |  | Rhine | Germany |
| 16 | Overshot | Holnermühle |  | Rhine | Germany |
| 16 | Overshot | Wiesenbauernhof Mühle |  | Rhine | Germany |
| 16 | Overshot | Löhr Mühle |  | Rhine | Germany |
| 16 | Overshot | Oberste Muhle/Rorhmuhle | Merzbach | Rhine | Germany |
| 16 | Overshot | Görtemöllers Mühle |  | Rhine | Germany |
| 16 | Overshot | Millicher Muhle | Golkrather Bach | Rhine | Germany |
| 16 | Overshot | Pletsch Mühle |  | Rhine | Germany |
| 16 | Overshot |  |  | Rhine | Germany |
| 16 | Overshot | Unterste Muhle | Merzbach | Rhine | Germany |
| 16 | Overshot | Klostermühle |  | Rhine | Germany |
| 16 | Overshot | Doverhahner muhle | Muhlenbach | Rhine | Germany |
| 16 | Overshot | Steffensmühle | Golkrather Bach | Rhine | Germany |
| 16 | Overshot | Unterschlauersbacher Mühle |  | Rhine | Germany |
| 16 | Overshot | Wassermühle Irsen | Irser Bach | Rhine | Germany |
| 16 | Overshot | Schönbachhofmühle |  | Rhine | Germany |
| 16 | Overshot | Schloß Mühle |  | Rhine | Germany |
| 16 | Overshot | Brenscheider Kornmuhle | Nahmerbach | Rhine | Germany |
| 16 | Overshot | Johann Adamsmühle |  | Rhine | Germany |
| 16 | Overshot | Müllerjörgenhofmühle |  | Rhine | Germany |
| 16 | Overshot | Schmalsmühle |  | Rhine | Germany |
| 16 | Overshot | Krulsmuhle |  | Rhine | Germany |
| 16 | Overshot | Varlarer Muhle/GroBe Muhle |  | Rhine | Germany |
| 16 | Overshot | Laachmühle/ Unteremühle | Pützbornerbach | Rhine | Germany |
| 16 | Overshot | Untere Strohner Mühle | Alfbach | Rhine | Germany |
| 16 | Overshot | Schattenmühle | Wutachschlucht | Rhine | Germany |
| 16 | Overshot | Alte Mühle |  | Rhine | Germany |
| 16 | Overshot | Alte Muhle |  | Rhine | Germany |
| 16 | Overshot | Wassermuhle Rurupsmuhle |  | Rhine | Germany |
| 16 | Overshot | Pickartzsche Muhle | Kirchberger Muhlenteich | Rhine | Germany |
| 16 | Overshot | Faust Mühle |  | Rhine | Germany |
| 16 | Overshot | Hasenhofmühle |  | Rhine | Germany |
| 16 | Overshot | Dinkelmühle St. Benignus |  | Rhine | Germany |
| 16 | Overshot | Klostermühle |  | Rhine | Germany |
| 16 | Overshot | Papiermühle |  | Rhine | Germany |
| 16 | Undershot | Bruchmuhle | Rehrbach | Rhine | Germany |
| 16 | Undershot |  | Rur | Rhine | Germany |
| 16 | Undershot | Kornmühle | Ahr | Rhine | Germany |
| 16 | Undershot | Brempter Muhle | Schwalm | Rhine | Germany |
| 16 | Undershot | Ruricher Schlossmuhle | Malefink | Rhine | Germany |
| 16 | Undershot | Völmeder Müller | Mühlenbach | Rhine | Germany |
| 16 | Undershot | Lochmühle |  | Rhine | Germany |
| 16 | Undershot | Hahnensteiner Mühle | Ahr | Rhine | Germany |
| 16 | Undershot | Buschmuhle | Muhlenbach | Rhine | Germany |
| 16 | Undershot | Muhle Harff | Krauthausener Muhlenteich | Rhine | Germany |
| 16 | Undershot | Kirchmühle Pfungstadt | Modau | Rhine | Germany |
| 16 | Undershot | Hanfreibe |  | Rhine | Germany |
| 16 | Undershot | Klostermühle | Pforzener Mühlbach | Rhine | Germany |
| 16 | Undershot | Brückenmühle |  | Rhine | Germany |
| 16 | Undershot | Furlmuhle |  | Rhine | Germany |
| 16 | Undershot | Alte Muhle | Moersbach | Rhine | Germany |
| 16 | Undershot | Kellersberger Muhle | Broicher Bach | Rhine | Germany |
| 16 | Undershot | Ol und Kornmuhle des WasserschloB Anholt | Issel | Rhine | Germany |
| 16 | Unknown | Hainmühle | Ohm | Rhine | Germany |
| 16 | Unknown | Molzmühle | Schwalm | Rhine | Germany |
| 16 | Unknown | Salzeder Mühle |  | Rhine | Germany |
| 16 | Unknown | Güdderather Mühle | Niers | Rhine | Germany |
| 16 | Unknown | Taubermühle | Tauber | Rhine | Germany |
| 16 | Unknown | Schalkenmehrener Mühle/ Liesermühle/ Häringsmühle | Maarbach | Rhine | Germany |
| 16 | Unknown |  |  | Rhine | Germany |
| 16 | Unknown | Stadtmühle |  | Rhine | Germany |
| 16 | Unknown | Zinßer Mühle |  | Rhine | Germany |
| 16 | Unknown | Alte Ruthe Mühle |  | Rhine | Germany |
| 16 | Unknown | Stegmühle |  | Rhine | Germany |
| 16 | Unknown | Volkardeyer Mühle | Schwarzbach | Rhine | Germany |
| 16 | Unknown | Wintersmühle | Bullerbach | Rhine | Germany |
| 16 | Unknown | Obere Mühle |  | Rhine | Germany |
| 16 | Unknown | Höschenmühle | Friesenbach | Rhine | Germany |
| 16 | Unknown | Obermühle |  | Rhine | Germany |
| 16 | Unknown | Stadtmuhle | Alme | Rhine | Germany |
| 16 | Unknown | Knottenmühle | Beise | Rhine | Germany |
| 16 | Unknown | Horster Muhle | Itterbach | Rhine | Germany |
| 16 | Unknown | Belgenbacher Mühle |  | Rhine | Germany |
| 16 | Unknown | Vorster Muhle | Nette | Rhine | Germany |
| 16 | Unknown | Broßhauser Mühle |  | Rhine | Germany |
| 16 | Unknown | Overbacher Mühle | Kirchberger Mühlenteich | Rhine | Germany |
| 16 | Unknown | Öl und Kornmühle | Junge Wurm | Rhine | Germany |
| 16 | Unknown | Bischofsmühle | Wilden Rodach | Rhine | Germany |
| 16 | Unknown | Sägemühle Kiefel/ Aumühle |  | Rhine | Germany |
| 16 | Unknown | Schwanenmühle | Rietherbach | Rhine | Germany |
| 16 | Unknown | Karker Muhle | Muhlenbach/Junge Wurm | Rhine | Germany |
| 16 | Unknown | Winkelsmühle |  | Rhine | Germany |
| 16 | Unknown | Mohrenmuhle | Rodebach | Rhine | Germany |
| 16 | Unknown | Binsen Mühle |  | Rhine | Germany |
| 16 | Unknown | Langenbrucker Muhle |  | Rhine | Germany |
| 16 | Unknown | Hammerwerk | Schwarzen Ahe | Rhine | Germany |
| 16 | Unknown | Obere Mühle |  | Rhine | Germany |
| 16 | Unknown | Wegberger Muhle | Schwalm | Rhine | Germany |
| 16 | Unknown | Bardenberger Mühle/ Alte Mühle | Wurm | Rhine | Germany |
| 16 | Unknown | Mellert Mühle |  | Rhine | Germany |
| 16 | Unknown | Schälmühle | Inde | Rhine | Germany |
| 16 | Unknown | Außere Mühle |  | Rhine | Germany |
| 16 | Unknown | Bischofsmuhle | Schwalm | Rhine | Germany |
| 16 | Unknown | Herrnmühle | Gollach | Rhine | Germany |
| 16 | Unknown | Feckweiler Ölmühle |  | Rhine | Germany |
| 16 | Unknown | Rats-/Herrenmühle |  | Rhine | Germany |
| 16 | Unknown | Ruppelmühle | Fränkische Saale | Rhine | Germany |
| 16 | Unknown | Dreisbachmühle | Dreisbach | Rhine | Germany |
| 16 | Unknown | Obere Steinborner Mühle |  | Rhine | Germany |
| 16 | Unknown | Turnemühle/ Thulenmühle/ Thornemühle | Salm | Rhine | Germany |
| 16 | Unknown | Oberstadtfelder Mühle | Kleinen Kyll | Rhine | Germany |
| 16 | Unknown | Mühle bei Haus Steinfurt | Alte Werse | Rhine | Germany |
| 16 | Unknown | Dorfmühle |  | Rhine | Germany |
| 16 | Unknown | Jörgenmühle/ Jörgleinsmühle | Gollach | Rhine | Germany |
| 16 | Unknown | Obermuhle Blatzheim | Neffelbach | Rhine | Germany |
| 16 | Unknown | Hinkels Mühle | Kleinen Kyll | Rhine | Germany |
| 16 | Unknown | Dorselermühle/ Ahrenbergschemühle |  | Rhine | Germany |
| 16 | Unknown | Daubians Mühle | Armutsbach | Rhine | Germany |
| 16 | Unknown | Alte Mühle |  | Rhine | Germany |
| 16 | Unknown | Mineralmühle |  | Rhine | Germany |
| 16 | Unknown | Untere Mühle | Eberbach | Rhine | Germany |
| 16 | Unknown | Keller'sche Mühle |  | Rhine | Germany |
| 16 | Unknown | Herrenmühle | Möhlin | Rhine | Germany |
| 16 | Unknown | Schimmelmühle |  | Rhine | Germany |
| 16 | Unknown | Hofbräuhaus Kunstmühle |  | Rhine | Germany |
| 16 | Overshot | Oliemolen | Caumerbeek | Meuse | Netherlands |
| 16 | Overshot | Molen van Hulsen / Onderste Molen | Molenbeek | Meuse | Netherlands |
| 16 | Undershot | Borgermolen | Geleenbeek | Meuse | Netherlands |
| 16 | Undershot | De Boxtelsche Watermoolen |  | Meuse | Netherlands |
| 16 | Undershot | Heisterbruggermolen | Geleenbeek | Meuse | Netherlands |
| 16 | Undershot | Gennepermolen | Dommel | Meuse | Netherlands |
| 16 | Unknown | Stadbroekermolen | Geleenbeek | Meuse | Netherlands |
| 16 | Unknown | Grevenbichtermolen | Kingbeek | Meuse | Netherlands |
| 16 | Unknown | Stratumse watermolen | Dommel | Meuse | Netherlands |
| 16 | Undershot | Molen Herinckhave | Fleringer Molenbeek | Rhine | Netherlands |
| 16 | Undershot | Oostendorper Molen | Buurserbeek | Rhine | Netherlands |
| 16 | Undershot | Nieuwe molen | Bolksbeek | Rhine | Netherlands |
| 16 | Undershot | Korenmolen van de Heus / Tolmolen |  | Rhine | Netherlands |
| 16 | Unknown | Haarmolen / Hamolle / Veddersmolen | Buursche Beek | Rhine | Netherlands |
| 16 | Unknown | De Polijstmolen | Zwarte water | Rhine | Netherlands |
| 16 | Unknown |  | Heksenlaak | Rhine | Netherlands |
| 17 | Breastshot | Moulin Ruwet | Bollandbeek | Meuse | Belgium |
| 17 | Breastshot | Moulin Pirson | Geer (Jeker) | Meuse | Belgium |
| 17 | Breastshot | Machine Elevatrice | Hoyoux | Meuse | Belgium |
| 17 | Breastshot | Moulin de Huccorgne, Ancien Moulin | Awirs | Meuse | Belgium |
| 17 | Breastshot | Moulin d-Olenne | Houille | Meuse | Belgium |
| 17 | Overshot | Moulin de Cherian |  | Meuse | Belgium |
| 17 | Overshot | Moulin de Mabompre, Moulin de Vellereux, Moulin Wuilart | Petite Eau et Rau de Mabompre | Meuse | Belgium |
| 17 | Overshot | Moulin de Fanzel |  | Meuse | Belgium |
| 17 | Overshot | Moulin Wipeur | Burnot | Meuse | Belgium |
| 17 | Overshot | Vieux Moulin | Ruisseau | Meuse | Belgium |
| 17 | Overshot | Moulin Mignolet |  | Meuse | Belgium |
| 17 | Overshot | Moulin de Bende | Ry de Bende | Meuse | Belgium |
| 17 | Overshot | Moulin de ways | Dijle | Meuse | Belgium |
| 17 | Overshot | Moulin de Vaux | Wee | Meuse | Belgium |
| 17 | Overshot | Moulin Leval, Moulin Paggen | Bolland | Meuse | Belgium |
| 17 | Overshot | Moulin d-Annevoie, le Blute Fin, Moulin d-Henryette |  | Meuse | Belgium |
| 17 | Overshot | Vieux Moulin, Moulin de Marneffe | Burdinalle | Meuse | Belgium |
| 17 | Overshot | Moulin de soulme | Hermenot | Meuse | Belgium |
| 17 | Overshot | Moulin de Lisogne | Fonds de Leffe | Meuse | Belgium |
| 17 | Overshot | Setzermuhle | Elterbach | Meuse | Belgium |
| 17 | Overshot | Moulin de Gotalle | Ourthe | Meuse | Belgium |
| 17 | Overshot | Molen van Moelingen | Berwinne | Meuse | Belgium |
| 17 | Overshot | Moulin du Blocquay | Li Ri d'Bloke | Meuse | Belgium |
| 17 | Overshot | Neuendorfmuhle | Moderbach | Meuse | Belgium |
| 17 | Overshot | Moulin du Ry de Mosbuex | Mosbeux | Meuse | Belgium |
| 17 | Overshot | Moulin Leonard |  | Meuse | Belgium |
| 17 | Overshot | Bornermuhle | Emmelsbach | Meuse | Belgium |
| 17 | Overshot | Moulin du Toultia | Ri de Dreye | Meuse | Belgium |
| 17 | Overshot | Moulin de Neuville | Morissartbeek | Meuse | Belgium |
| 17 | Overshot | Moulin de Martigny | Wamme | Meuse | Belgium |
| 17 | Overshot | Moulin de Daverdisse | Almache | Meuse | Belgium |
| 17 | Overshot | Moulin des 3 Ponts | Ourthe | Meuse | Belgium |
| 17 | Overshot | Laminoirs de Cuivre (2) | Molignee | Meuse | Belgium |
| 17 | Overshot | abdij van Grandpre | Samson | Meuse | Belgium |
| 17 | Overshot | Moulin de Balatre | La Ligne | Meuse | Belgium |
| 17 | Overshot | Moulin de la Ferme | Rue du Meunier | Meuse | Belgium |
| 17 | Overshot | Moulin de Treignes | Viroin | Meuse | Belgium |
| 17 | Overshot | Moulin de Chateau, Moulin de Bierbaix | Houssiere | Meuse | Belgium |
| 17 | Overshot | Moulin de Thill, Moulin de thy | Thyle | Meuse | Belgium |
| 17 | Overshot | Moulin a Poudre | Orne | Meuse | Belgium |
| 17 | Overshot | Moulin de la Haie |  | Meuse | Belgium |
| 17 | Overshot | Moulin Banal |  | Meuse | Belgium |
| 17 | Overshot | Moulin a papier, Moulin Gantois |  | Meuse | Belgium |
| 17 | Turbine | Moulin Neuf | Warch | Meuse | Belgium |
| 17 | Turbine | Moulin de Fontenelle |  | Meuse | Belgium |
| 17 | Turbine | Moulin de Spiennes |  | Meuse | Belgium |
| 17 | Turbine | Moulin Lemye |  | Meuse | Belgium |
| 17 | Turbine | Dorpsmolen / Molen Lievens | Nieuwe Herk | Meuse | Belgium |
| 17 | Turbine | Moulin Fraiklin | Geer | Meuse | Belgium |
| 17 | Turbine | Van Helmontmolen, Gentemolen |  | Meuse | Belgium |
| 17 | Turbine | Moulin de Natoye | Bocq | Meuse | Belgium |
| 17 | Turbine | Moulin d-Irchonwelz |  | Meuse | Belgium |
| 17 | Turbine | Moulin de Viesville |  | Meuse | Belgium |
| 17 | Undershot | Galdermansmolen | Itterbeek | Meuse | Belgium |
| 17 | Undershot | Smokkelmolen, Kleine Molen | Jeker | Meuse | Belgium |
| 17 | Undershot | Rotemse Molen | Velpe | Meuse | Belgium |
| 17 | Undershot | Molen van Bellefontaine | Jeker | Meuse | Belgium |
| 17 | Undershot | Molen van Opkanne | Jeker | Meuse | Belgium |
| 17 | Undershot | Vieux Moulin |  | Meuse | Belgium |
| 17 | Undershot | Slagmolen, Molen Stals, Scheelenmolen | Itterbeek | Meuse | Belgium |
| 17 | Undershot | Moulin de Hosdent | Mehaigne | Meuse | Belgium |
| 17 | Undershot | Moulin du Verre d-Eau |  | Meuse | Belgium |
| 17 | Undershot | Grote Molen | Demer | Meuse | Belgium |
| 17 | Undershot | Moulin de lacuisine, Vieux Moulin |  | Meuse | Belgium |
| 17 | Undershot | Moulin de termes | Semois | Meuse | Belgium |
| 17 | Undershot | Moulin du Mouflu |  | Meuse | Belgium |
| 17 | Undershot | Moulin du Stordoir |  | Meuse | Belgium |
| 17 | Breastshot | Haarmühle | Ahauser Aa | Rhine | Germany |
| 17 | Breastshot | Paumuhle | Rotbach | Rhine | Germany |
| 17 | Breastshot | Kammerer's Mühle | Wiesent | Rhine | Germany |
| 17 | Breastshot | Bohners Muhle |  | Rhine | Germany |
| 17 | Overshot | Queck Mühle |  | Rhine | Germany |
| 17 | Overshot | Rhader Mühle/ Ellefahrt Mühle |  | Rhine | Germany |
| 17 | Overshot | Hausmahlmühle |  | Rhine | Germany |
| 17 | Overshot | Bromberger Mühle |  | Rhine | Germany |
| 17 | Overshot | Bromberger Mühle |  | Rhine | Germany |
| 17 | Overshot | Vaihinghöfer Mühle/ Hummelgautsche Mühle |  | Rhine | Germany |
| 17 | Overshot | Rindenmühle |  | Rhine | Germany |
| 17 | Overshot | Mühle Hof zur Osten |  | Rhine | Germany |
| 17 | Overshot | Herrenmühle | Nüst | Rhine | Germany |
| 17 | Overshot | Schloßmühle |  | Rhine | Germany |
| 17 | Overshot | Untere Muhle |  | Rhine | Germany |
| 17 | Overshot | Obere Muhle |  | Rhine | Germany |
| 17 | Overshot | Husen Mühle | Lusbach | Rhine | Germany |
| 17 | Overshot | Wassermühle Embken/ Nicksmühle | Muldenauer Bach | Rhine | Germany |
| 17 | Overshot | Alte Ölmühle | Klemmbach | Rhine | Germany |
| 17 | Overshot | Glockenschmiede |  | Rhine | Germany |
| 17 | Overshot | Alte Dorfmühle |  | Rhine | Germany |
| 17 | Overshot | Mestrenger Mühle |  | Rhine | Germany |
| 17 | Overshot | Wassermühle Schulzenhof Westerath | Stever | Rhine | Germany |
| 17 | Overshot | Hammerschmiede |  | Rhine | Germany |
| 17 | Overshot | Olmuhle Husen |  | Rhine | Germany |
| 17 | Overshot | Sägemühle Remblinghausen |  | Rhine | Germany |
| 17 | Overshot | Dorfsmuhle | Muhlenbach | Rhine | Germany |
| 17 | Overshot | Dorfsmühle | Mühlenbach | Rhine | Germany |
| 17 | Overshot | Stapelsmühle | Münsterischen Aa | Rhine | Germany |
| 17 | Overshot | Vetter's Mühle |  | Rhine | Germany |
| 17 | Overshot | Drahtzieherei Hamelsrolle |  | Rhine | Germany |
| 17 | Overshot | SchloBmuhle |  | Rhine | Germany |
| 17 | Overshot | Künerhofmühle |  | Rhine | Germany |
| 17 | Overshot | Jockeleshof Mühle |  | Rhine | Germany |
| 17 | Overshot | Rohrhirsch Mühle |  | Rhine | Germany |
| 17 | Overshot | Mooswald Mühle |  | Rhine | Germany |
| 17 | Overshot | Schwanenmühle |  | Rhine | Germany |
| 17 | Overshot | Katzbrui Mühle |  | Rhine | Germany |
| 17 | Overshot | Untermühle/ Tüten Mühle | Amstelbach | Rhine | Germany |
| 17 | Undershot | Kurfurstliche Muhle | Linner Muhlenbach | Rhine | Germany |
| 17 | Undershot | Vollmuhle tuddern | Rodebach | Rhine | Germany |
| 17 | Undershot | Erpenbecks Getreidemühle |  | Rhine | Germany |
| 17 | Undershot | Hilzinger Mühle |  | Rhine | Germany |
| 17 | Undershot | Wassermühle Haus Welbergen | Vechte | Rhine | Germany |
| 17 | Undershot | Ruhmühle/ Mühlhansenmühle |  | Rhine | Germany |
| 17 | Undershot | Ophover Mühle | Becker Bach (Schwalm) | Rhine | Germany |
| 17 | Undershot | Olmuhle Brake |  | Rhine | Germany |
| 17 | Undershot | Sägemühle Weisbach |  | Rhine | Germany |
| 17 | Undershot | Wassermühle Eismann/ Stiftsmühle |  | Rhine | Germany |
| 17 | Undershot | Obere Burgmühle | Oberer Schermbecker Mühlenbach | Rhine | Germany |
| 17 | Undershot | Senfmühle |  | Rhine | Germany |
| 17 | Undershot | Bockenmühle | Schwalm | Rhine | Germany |
| 17 | Undershot | Schrofmühle | Mühlenbach (Schwalm) | Rhine | Germany |
| 17 | Undershot | Kornmuhle Tuddern | Rodebach | Rhine | Germany |
| 17 | Undershot | Pannenmühle | Schwalm | Rhine | Germany |
| 17 | Undershot | Johanntgesbrucher Muhle | Weinberger bach | Rhine | Germany |
| 17 | Undershot | Ölmühle | Ahr | Rhine | Germany |
| 17 | Undershot | Gnadentaler Mühle | Erft | Rhine | Germany |
| 17 | Undershot | Ölmühle | Wied | Rhine | Germany |
| 17 | Undershot | Neubrückermühle | Erft | Rhine | Germany |
| 17 | Undershot | Alte Olmuhle/Sonsmuhle | Ahr | Rhine | Germany |
| 17 | Undershot | Neuemühle | Aa | Rhine | Germany |
| 17 | Undershot | Muhle an der SuBbrucke | Niers | Rhine | Germany |
| 17 | Undershot | Alte Wassermühle | Teufelsbach | Rhine | Germany |
| 17 | Undershot | Brodmühle |  | Rhine | Germany |
| 17 | Unknown | Ammerndorfer Mühle |  | Rhine | Germany |
| 17 | Unknown | Papiermuhle Lamersdorf | Inde | Rhine | Germany |
| 17 | Unknown | Mühle Boll |  | Rhine | Germany |
| 17 | Unknown | Huxmühle | Laubach | Rhine | Germany |
| 17 | Unknown | Tüshaus Mühle | Hammbach | Rhine | Germany |
| 17 | Unknown | Weidenbacher Mühle | Salm | Rhine | Germany |
| 17 | Unknown | Henkenmühle |  | Rhine | Germany |
| 17 | Unknown | Ölmühle Kirner |  | Rhine | Germany |
| 17 | Unknown | Obere Mühle |  | Rhine | Germany |
| 17 | Unknown | Hainmühle |  | Rhine | Germany |
| 17 | Unknown | Riesenmühle |  | Rhine | Germany |
| 17 | Unknown | Schmausemühle |  | Rhine | Germany |
| 17 | Unknown | Getreide Mühle Luz |  | Rhine | Germany |
| 17 | Unknown | papiermuhle Einbrungen | Schwarzbach | Rhine | Germany |
| 17 | Unknown | Stadtmühle |  | Rhine | Germany |
| 17 | Unknown | Wassermuhle Brauersdorf | julicher Muhlenteich | Rhine | Germany |
| 17 | Unknown | Mühlemuseum Schloß Brake |  | Rhine | Germany |
| 17 | Unknown | Kornmuhle | Niers | Rhine | Germany |
| 17 | Unknown | Huttermuhle | Kranenbach | Rhine | Germany |
| 17 | Unknown | Riedl |  | Rhine | Germany |
| 17 | Unknown | Offergeld'sche Mühle | Kirchberger Mühlenteich | Rhine | Germany |
| 17 | Unknown | Wassermühle Haus Vorhelm | Hellbach | Rhine | Germany |
| 17 | Unknown | Nette Mühle | Nette | Rhine | Germany |
| 17 | Unknown | Stadtmühle | Umflut | Rhine | Germany |
| 17 | Unknown | Ölmühle | Niers | Rhine | Germany |
| 17 | Unknown | Berkelkraftwerk v/h Stiftsmuhle |  | Rhine | Germany |
| 17 | Unknown | Belzmühle |  | Rhine | Germany |
| 17 | Unknown | Klostermühle |  | Rhine | Germany |
| 17 | Unknown | Jäckle Mühle |  | Rhine | Germany |
| 17 | Unknown | Justus Mühle |  | Rhine | Germany |
| 17 | Unknown | walk-/Olmuhle von Hs. Caen | Niers | Rhine | Germany |
| 17 | Unknown | Sonsmuhle | Dollendorfer StraBe 2 | Rhine | Germany |
| 17 | Unknown | Neumühle | Fränkische Saale | Rhine | Germany |
| 17 | Unknown | Broicher Muhle | Broicherbach | Rhine | Germany |
| 17 | Unknown | SchloBmuhle myllendonk | Niers | Rhine | Germany |
| 17 | Unknown | Löffelmühle |  | Rhine | Germany |
| 17 | Unknown | Kugelmühle | Almbach | Rhine | Germany |
| 17 | Unknown | Ölmühle | Stever | Rhine | Germany |
| 17 | Unknown |  |  | Rhine | Germany |
| 17 | Unknown |  |  | Rhine | Germany |
| 17 | Unknown | Weihermühle | Kiedrichbach | Rhine | Germany |
| 17 | Unknown | Trotzmühle | Laubach | Rhine | Germany |
| 17 | Unknown | Königsmühler Kotten | Weinberger Bach | Rhine | Germany |
| 17 | Unknown | Wassermühle Eilhausen | Mühlenteich | Rhine | Germany |
| 17 | Unknown | Kottenborner Mühle | Wirftbach | Rhine | Germany |
| 17 | Unknown | Weißmühle |  | Rhine | Germany |
| 17 | Unknown | Fellenberg Mühle | Seffersbach | Rhine | Germany |
| 17 | Unknown | Kunz`sche Mühle |  | Rhine | Germany |
| 17 | Unknown | Herrenmühle |  | Rhine | Germany |
| 17 | Breastshot | Bisschopsmolen | Jeker, Noordtak | Meuse | Netherlands |
| 17 | Breastshot | Molen Van Lombok | Jeker | Meuse | Netherlands |
| 17 | Breastshot | Vaalsbroekermolen | Zievers | Meuse | Netherlands |
| 17 | Breastshot | Poolmolen | Geleenbeek | Meuse | Netherlands |
| 17 | Breastshot | Slagmolen | Molenbeek | Meuse | Netherlands |
| 17 | Breastshot | Geulhemmermolen | Geul | Meuse | Netherlands |
| 17 | Breastshot | Weltermolen | Geleenbeek | Meuse | Netherlands |
| 17 | Breastshot | Streythagermolen | Kasteelvijver | Meuse | Netherlands |
| 17 | Overshot | De Onderste Molen | Molenbeek | Meuse | Netherlands |
| 17 | Overshot | Kasteelmolen / Slakmolen | Slakbeek | Meuse | Netherlands |
| 17 | Overshot | Meschermolen | Voer | Meuse | Netherlands |
| 17 | Overshot | Molen van Hout-Blerick | Springbeek | Meuse | Netherlands |
| 17 | Under and Overshot | Kasteelmolen / Wymarse Molen | Lingsforterbeek | Meuse | Netherlands |
| 17 | Undershot | Friedesse Molen | Neerbeek | Meuse | Netherlands |
| 17 | Undershot | Schouwmolen | Itterbeek | Meuse | Netherlands |
| 17 | Undershot |  | Vlier | Meuse | Netherlands |
| 17 | Undershot | Rosmolen | Oostrumse Beek | Meuse | Netherlands |
| 17 | Undershot | De Beekse Molen | Molenbeek | Meuse | Netherlands |
| 17 | Undershot | De Armenmolen | Itterbeek | Meuse | Netherlands |
| 17 | Undershot | Molen van Baarlo | Kwistbeek | Meuse | Netherlands |
| 17 | Unknown | Bovenste Molen | Rode Beek | Meuse | Netherlands |
| 17 | Unknown | Oude of Banmolen | Geul | Meuse | Netherlands |
| 17 | Unknown | Kaarschotse Molen / Watermolen van Kaarschot |  | Meuse | Netherlands |
| 17 | Unknown | (S)chaloensmolen |  | Meuse | Netherlands |
| 17 | Unknown | Panheeldermolen / Molen van het Bosmolenveld | Panheelderbeek | Meuse | Netherlands |
| 17 | Overshot | De Ruitersmolen | Beekbergse Beek | Rhine | Netherlands |
| 17 | Overshot | De Stinkmolens |  | Rhine | Netherlands |
| 17 | Overshot |  | Eerbeekse Beek | Rhine | Netherlands |
| 17 | Overshot | Molen de Oorsprong |  | Rhine | Netherlands |
| 17 | Undershot | De Oude Molen / Altena | Steenbeek | Rhine | Netherlands |
| 17 | Undershot | Grote Volmolen |  | Rhine | Netherlands |
| 17 | Undershot |  | Oude Diep | Rhine | Netherlands |
| 17 | Undershot | Oldemeule / Oelemeulle | Oelerbeek | Rhine | Netherlands |
| 17 | Undershot |  | Baaksebeek | Rhine | Netherlands |
| 17 | Unknown | De Pelmolen |  | Rhine | Netherlands |
| 17 | Unknown | Bazemolens | Steenbeek | Rhine | Netherlands |
| 17 | Unknown | De Rotterdamse Kopermolen |  | Rhine | Netherlands |
| 17 | Unknown | Pannekoeksmolen | Wenumsebeek | Rhine | Netherlands |
| 17 | Unknown | Middelste molen op het Loo | Loosebeek | Rhine | Netherlands |
| 17 | Unknown | Hattemmolen |  | Rhine | Netherlands |
| 17 | Unknown | Tiemensmolen |  | Rhine | Netherlands |
| 17 | Unknown | Winkewijermolen | Winkewijert | Rhine | Netherlands |
| 17 | Unknown | Eendracht |  | Rhine | Netherlands |
| 17 | Unknown | Het Liertje |  | Rhine | Netherlands |
| 17 | Unknown | Methusalem |  | Rhine | Netherlands |
| 17 | Unknown | Kayersmolen |  | Rhine | Netherlands |
| 17 | Unknown | Bovenste molen op het Loo | Loosebeek | Rhine | Netherlands |
| 17 | Unknown | Vingerhoedmolen | aan de Biltse Grift | Rhine | Netherlands |
| 17 | Unknown | Het Voorslop |  | Rhine | Netherlands |
| 17 | Unknown | Papiermolen Steenbeek | Steenbeek | Rhine | Netherlands |
| 17 | Unknown | Dijkgraafsmolen | Wenumsebeek | Rhine | Netherlands |
| 17 | Unknown | Klein Bazemolentje | Schoolbeek | Rhine | Netherlands |
| 17 | Unknown | Groevenbeekse Molen | Groevenbeek | Rhine | Netherlands |
| 17 | Unknown | Molen bij de Vanenburg | Groevenbeek | Rhine | Netherlands |
| 17 | Unknown | De Wasmolen / Staverdense Molen | Staverdensebeek | Rhine | Netherlands |
| 17 | Unknown | Huygensmolen | Wenumse beek | Rhine | Netherlands |
| 17 | Unknown | Korenmolen van Gardenbroek | Beek van het Loo/Veldhuizen | Rhine | Netherlands |
| 17 | Unknown | De Werfhorst |  | Rhine | Netherlands |
| 17 | Unknown | De Zandmolen 1 | Leuvenumebeek | Rhine | Netherlands |
| 17 | Unknown | Molen bij Schoonderbeek | Schoonderbeek | Rhine | Netherlands |
| 17 | Unknown | Papiermolen bij de Wildkamp | Wenumsebeek | Rhine | Netherlands |
| 17 | Unknown | Bovenste Brouwersmolen | Orderbeek | Rhine | Netherlands |
| 17 | Unknown | Molen bij de Schaffelaar | Papiermolenbeekje | Rhine | Netherlands |
| 18 | Breastshot | Meylandtmolen, Oude Molen |  | Meuse | Belgium |
| 18 | Breastshot | Dorpsmolen | Bosbeek | Meuse | Belgium |
| 18 | Breastshot | Moulin d-Houx | Zenne | Meuse | Belgium |
| 18 | Breastshot | Moulin Lhoist, moulin de Booze | Bolland | Meuse | Belgium |
| 18 | Breastshot | Moulin de walzin | Lesse | Meuse | Belgium |
| 18 | Breastshot | Moulin de Vencimont | Houille | Meuse | Belgium |
| 18 | Overshot | Le Moulinet |  | Meuse | Belgium |
| 18 | Overshot | Moulin de Gros-Fays |  | Meuse | Belgium |
| 18 | Overshot | Moulin Collard | Magne | Meuse | Belgium |
| 18 | Overshot | Moulin Clement | Ocquier | Meuse | Belgium |
| 18 | Overshot | Moulin de Glimes | Grand-Ry | Meuse | Belgium |
| 18 | Overshot | Moulin de Loupoigne | Dijle | Meuse | Belgium |
| 18 | Overshot | Moulin Dussart, Moulin Sebille, Moulin du Tordoir | Ry du Grignard | Meuse | Belgium |
| 18 | Overshot | Moulin de la Ferme Saint-Michel/Ferme du Moulin | Thorembais | Meuse | Belgium |
| 18 | Overshot | Moulin du Ry de Vaux | Vaux | Meuse | Belgium |
| 18 | Overshot | Moulin Bolland, Moulin Wathieu | La Lize | Meuse | Belgium |
| 18 | Overshot | Moulin de la Chapelle | La Magree | Meuse | Belgium |
| 18 | Overshot | Moulin de Labiwez | Thines | Meuse | Belgium |
| 18 | Overshot | Le Franc Moulin, Moulin Mricq, Le Nouveau | Le Train | Meuse | Belgium |
| 18 | Overshot | Moulin d-Hastiere, moulin Baeken |  | Meuse | Belgium |
| 18 | Overshot | Moulin d-Angre |  | Meuse | Belgium |
| 18 | Overshot | Ancien moulin banal | Mehaigne | Meuse | Belgium |
| 18 | Overshot | Fourneau Saint-Michel |  | Meuse | Belgium |
| 18 | Overshot | Moulin de Bistian | Ourthe | Meuse | Belgium |
| 18 | Overshot | Moulin Sauvegarde |  | Meuse | Belgium |
| 18 | Overshot | Moulin Grenade | Grand Aaz | Meuse | Belgium |
| 18 | Overshot | Moulin de Petigny | Ry de Rome | Meuse | Belgium |
| 18 | Overshot | Moulin Coppee |  | Meuse | Belgium |
| 18 | Overshot | Moulin de Lasne | Lasne | Meuse | Belgium |
| 18 | Overshot | Moulin Gilles, Moulin d-Ecdoval | Groumont | Meuse | Belgium |
| 18 | Overshot | Forge Monseu, Moulin Crowet |  | Meuse | Belgium |
| 18 | Overshot | Moulin Seeliger | La Lize | Meuse | Belgium |
| 18 | Overshot | Neufmoulin | Ri Fond d-Oxhe | Meuse | Belgium |
| 18 | Overshot | Moulin de la Folie, Moulin de Ramponneau |  | Meuse | Belgium |
| 18 | Overshot | Durlermuhle | Ri De Ulf | Meuse | Belgium |
| 18 | Overshot | Moulin de Grune | canal du moulin de Grune | Meuse | Belgium |
| 18 | Overshot | Moulin Lebrun |  | Meuse | Belgium |
| 18 | Overshot | Moulin Klepper, Moulin Roland |  | Meuse | Belgium |
| 18 | Overshot | Usines de Moulins, Laminoirs de cuivre (1) | Molignee | Meuse | Belgium |
| 18 | Overshot | Moulin Neufmarteau, Moulin Valentin | Le Rew | Meuse | Belgium |
| 18 | Overshot | Moulin Henrard | Bolland | Meuse | Belgium |
| 18 | Overshot | Moulin Henrard, Moulin Thewissen, Moulin Brakovich | Bolland | Meuse | Belgium |
| 18 | Overshot | Moulin Esser | Baelen | Meuse | Belgium |
| 18 | Overshot | Scierie de Stadt | Hoyoux | Meuse | Belgium |
| 18 | Overshot | Moulin Bolette | Vaux | Meuse | Belgium |
| 18 | Overshot | Moulin de Burnontige, Moulin du Manoir du Chat qui fume | Wesomont | Meuse | Belgium |
| 18 | Overshot | Moulin de Comblain | Ambleve | Meuse | Belgium |
| 18 | Overshot | Moulin de la Chapelle | Rau de Tavier | Meuse | Belgium |
| 18 | Overshot | Moulin de Neblon | Neblon | Meuse | Belgium |
| 18 | Overshot | Moulin de La Vau | Rau du Fond Martin | Meuse | Belgium |
| 18 | Overshot | Moulin Roiseux | Hoyoux | Meuse | Belgium |
| 18 | Overshot | Moulin de Neufmoulin, Moulin Forge | Rau du Pouhon | Meuse | Belgium |
| 18 | Overshot | Moulin de Villance, moulin Lezin |  | Meuse | Belgium |
| 18 | Overshot | Moulin de Nollevaux |  | Meuse | Belgium |
| 18 | Overshot | Moulin de Rahimont, Moulin de Gives | Rouette | Meuse | Belgium |
| 18 | Overshot | Moulin de Gribomont, Moulin Maury | Vierre | Meuse | Belgium |
| 18 | Overshot | Moulin de Martilly | Vierre | Meuse | Belgium |
| 18 | Overshot | Mouin de la Queue de Vache | Rau du Fond de Genes | Meuse | Belgium |
| 18 | Overshot | Moulin de Rechivral, moulin Cravatte | Rau de Laval | Meuse | Belgium |
| 18 | Overshot | Moulin de Mirwart | Lomme | Meuse | Belgium |
| 18 | Overshot | Moulin de Seviscourt | Rau de Freux | Meuse | Belgium |
| 18 | Overshot | Moulin de Freux-Menil | Ruisseau de Freux | Meuse | Belgium |
| 18 | Overshot | Moulin de Sept | Ruisseau de Freux | Meuse | Belgium |
| 18 | Overshot | Moulin de Lignieres | La Hedree | Meuse | Belgium |
| 18 | Overshot | Moulin Durbu |  | Meuse | Belgium |
| 18 | Overshot | Moulin Galloy | Le Crupet | Meuse | Belgium |
| 18 | Overshot | Moulin des Ramiers |  | Meuse | Belgium |
| 18 | Overshot | Moulin de Gourdinne |  | Meuse | Belgium |
| 18 | Overshot | Moulin de la Ramee |  | Meuse | Belgium |
| 18 | Overshot | Moulin de Pry |  | Meuse | Belgium |
| 18 | Overshot | Moulin de Samart |  | Meuse | Belgium |
| 18 | Overshot | Scierie Bourtembourg |  | Meuse | Belgium |
| 18 | Overshot | Moulin Biron |  | Meuse | Belgium |
| 18 | Overshot | Moulin de Dailly |  | Meuse | Belgium |
| 18 | Overshot | Roue du Comte | Genee | Meuse | Belgium |
| 18 | Overshot | Moulin d-Hanzinelle | Rau de Tiria | Meuse | Belgium |
| 18 | Overshot | Moulin de Pree | Ruisseau d-Oret | Meuse | Belgium |
| 18 | Overshot | Moulin de Leuze | Somme | Meuse | Belgium |
| 18 | Overshot | Moulin d-Alpree |  | Meuse | Belgium |
| 18 | Overshot | Moulin Mitan, moulin de Gourey | Le Crupet | Meuse | Belgium |
| 18 | Overshot | Moulin d-Avillon | Le Crupet | Meuse | Belgium |
| 18 | Overshot | Moulin de la Galette | Houille | Meuse | Belgium |
| 18 | Overshot | Moulin Simon | Hulle | Meuse | Belgium |
| 18 | Overshot | Moulin de Fraire |  | Meuse | Belgium |
| 18 | Overshot | Moulin de Villers |  | Meuse | Belgium |
| 18 | Turbine | Moulin de Tenre | Dender | Meuse | Belgium |
| 18 | Turbine | Moulin des Roquettes |  | Meuse | Belgium |
| 18 | Turbine | Moulin de Robermont | Nethen | Meuse | Belgium |
| 18 | Turbine | Moulin Vanderschrieck |  | Meuse | Belgium |
| 18 | Turbine | Moulin de la Batterie, moulin de Strale | Grote Gete | Meuse | Belgium |
| 18 | Turbine | Abroxmolen | Abeek | Meuse | Belgium |
| 18 | Turbine | ourenmuhle | Schiebach | Meuse | Belgium |
| 18 | Turbine | Moulin Stock |  | Meuse | Belgium |
| 18 | Turbine | Moulin Desart |  | Meuse | Belgium |
| 18 | Turbine | Moulin del Rouge, Les Grniers du Moulin | Zenne | Meuse | Belgium |
| 18 | Turbine | Moulin Grosjean | Warche | Meuse | Belgium |
| 18 | Turbine | Scierie de Lepage | Chevralle | Meuse | Belgium |
| 18 | Turbine | Scierie Mahy |  | Meuse | Belgium |
| 18 | Turbine | Moulin de Barbonwez | Ourthe | Meuse | Belgium |
| 18 | Turbine | Scierie de marbre |  | Meuse | Belgium |
| 18 | Turbine | Le Maka, Usines Baugniet, Moulin des Saules | Grote Gete | Meuse | Belgium |
| 18 | Turbine | Tannerie Roorijk |  | Meuse | Belgium |
| 18 | Turbine | Moulin Williame | Dender | Meuse | Belgium |
| 18 | Turbine | Moulin Tordoir |  | Meuse | Belgium |
| 18 | Undershot | Slagmolen van Ellikom |  | Meuse | Belgium |
| 18 | Undershot | Moulin a Huile | Kliene Gete | Meuse | Belgium |
| 18 | Undershot | Leverenmolen | Bosbeek | Meuse | Belgium |
| 18 | Undershot | Het Mieleke, Nieuwe Molen | Bosbeek | Meuse | Belgium |
| 18 | Undershot | Kleine Molen | Mangelbeek | Meuse | Belgium |
| 18 | Undershot | Moulin Deborre | Geer (Jeker) | Meuse | Belgium |
| 18 | Undershot | Moulin Faber, Moulin de Hotton |  | Meuse | Belgium |
| 18 | Undershot | Meuleken | Voer | Meuse | Belgium |
| 18 | Undershot | Moulin de Hamteau, Moulin de Werpin | Werpin | Meuse | Belgium |
| 18 | Undershot | Dorpermolen |  | Meuse | Belgium |
| 18 | Undershot | Berenheidemolen, Achterste Molen, Slagmolen | Abeek | Meuse | Belgium |
| 18 | Undershot | Volmolen | Bosbeek | Meuse | Belgium |
| 18 | Undershot | Vieux Moulin |  | Meuse | Belgium |
| 18 | Undershot | Mombekermolen | Melbeek | Meuse | Belgium |
| 18 | Undershot | Moulin d-Ortheuville, moulin Couturier | Ourthe | Meuse | Belgium |
| 18 | Undershot | Wellermolen | Herk | Meuse | Belgium |
| 18 | Undershot | Wintershovenmolen | Mombeek | Meuse | Belgium |
| 18 | Undershot | Moulin de Chapelle | Lasne | Meuse | Belgium |
| 18 | Undershot | Moulin de Rochelinval | La Salm | Meuse | Belgium |
| 18 | Undershot | Nieuwe Molen, Ter Koestemolen | Nieuwe Herk | Meuse | Belgium |
| 18 | Undershot | Groenmolen | Nieuwe Herk | Meuse | Belgium |
| 18 | Undershot | Commelomolen | Zwarte Beek | Meuse | Belgium |
| 18 | Undershot | Gestelsemolen | Zwarte Beek | Meuse | Belgium |
| 18 | Undershot | Rooiermolen | Stiemerbeek | Meuse | Belgium |
| 18 | Undershot | Rootmolen, Molen van Knaepen |  | Meuse | Belgium |
| 18 | Undershot | Kasteelmolen, Vinckemolen, Molen van Aken | Itterbeek | Meuse | Belgium |
| 18 | Undershot | Dubbelmolen |  | Meuse | Belgium |
| 18 | Undershot | Hoogmolen | Abeek | Meuse | Belgium |
| 18 | Undershot | Moulin de Rensiwez | Ourthe | Meuse | Belgium |
| 18 | Undershot | Moulin Prignot | Aisne | Meuse | Belgium |
| 18 | Undershot | Moulin de Barse | Hoyoux | Meuse | Belgium |
| 18 | Undershot | Moulin Champagne |  | Meuse | Belgium |
| 18 | Undershot | Moulin d-Halleux | Ambleve | Meuse | Belgium |
| 18 | Undershot | Korenmolen |  | Meuse | Belgium |
| 18 | Undershot | Nieuwe Molen, Ter Koestemolen | Herk | Meuse | Belgium |
| 18 | Undershot | Houdenmolen | Bosbeek | Meuse | Belgium |
| 18 | Undershot | Vieille Forge | Lienne | Meuse | Belgium |
| 18 | Undershot | Moulin Loverix | Geer (Jeker) | Meuse | Belgium |
| 18 | Undershot | Moulin Dupuis | Geer (Jeker) | Meuse | Belgium |
| 18 | Undershot | Moulin Vassen | Vesdre | Meuse | Belgium |
| 18 | Undershot | Moulin de State, Ateliers Communaux | Hoyoux | Meuse | Belgium |
| 18 | Undershot | Moulin du val | Ossogne | Meuse | Belgium |
| 18 | Undershot | Moulin du Rouge-Thier | Le Fond Bastin | Meuse | Belgium |
| 18 | Undershot | Moulin de Sur-Villers | Ossogne | Meuse | Belgium |
| 18 | Undershot | Moulin de Moxhe | Mehaigne | Meuse | Belgium |
| 18 | Undershot | Moulin de Bonia | Burdinalle | Meuse | Belgium |
| 18 | Undershot | Moulin des Aulnes | OUrthe | Meuse | Belgium |
| 18 | Undershot | Moulin Lemaire |  | Meuse | Belgium |
| 18 | Undershot | Moulin de Bomal | Aisne | Meuse | Belgium |
| 18 | Undershot | Moulin de Rompre | Ourthe | Meuse | Belgium |
| 18 | Undershot | Moulin de la Hailleule | Vierre | Meuse | Belgium |
| 18 | Undershot | Moulin de Resteigne |  | Meuse | Belgium |
| 18 | Undershot | Moulin de Bauche | Bocq | Meuse | Belgium |
| 18 | Undershot | Moulin de spontin | Bocq | Meuse | Belgium |
| 18 | Undershot | Moulin des Bois | Ry de Rome | Meuse | Belgium |
| 18 | Undershot | Moulin Dony |  | Meuse | Belgium |
| 18 | Undershot | Moulin de Belvaux | Lesse | Meuse | Belgium |
| 18 | Undershot | Mouline de Felenne | Houille | Meuse | Belgium |
| 18 | Undershot | Roue Coleau |  | Meuse | Belgium |
| 18 | Undershot | Moulin de Rixensart, Le logis | Lasne | Meuse | Belgium |
| 18 | Undershot | Moulin des Bichurees |  | Meuse | Belgium |
| 18 | Undershot | Moulin Camille Vos, Moulin des Trieux | les Trieux | Meuse | Belgium |
| 18 | Undershot | la Venelle |  | Meuse | Belgium |
| 18 | Undershot | Moulin d'-Isieres | la Sille | Meuse | Belgium |
| 18 | Breastshot | Wassermuhle Hoping | Frischenhofsbach | Rhine | Germany |
| 18 | Breastshot | Mühle des 4 Hofes |  | Rhine | Germany |
| 18 | Breastshot | Klopf-Plotzsäge |  | Rhine | Germany |
| 18 | Overshot | Papiermuhle Ploger |  | Rhine | Germany |
| 18 | Overshot |  |  | Rhine | Germany |
| 18 | Overshot | Hammermühle | Modau/Hammerbach | Rhine | Germany |
| 18 | Overshot | Lochmühle | Elbbach | Rhine | Germany |
| 18 | Overshot | Wassermuhle Huas Rhade |  | Rhine | Germany |
| 18 | Overshot |  | Berkel | Rhine | Germany |
| 18 | Overshot | Untere Mühle/ Zweite Mühle | Watter | Rhine | Germany |
| 18 | Overshot | Ellerburger Muhle | Muhlbach | Rhine | Germany |
| 18 | Overshot |  |  | Rhine | Germany |
| 18 | Overshot | Straubenhofmühle |  | Rhine | Germany |
| 18 | Overshot | Kramers Wassermühle |  | Rhine | Germany |
| 18 | Overshot | Altenvogtshofmühle |  | Rhine | Germany |
| 18 | Overshot | Wendener Hütte | Bigge | Rhine | Germany |
| 18 | Overshot |  |  | Rhine | Germany |
| 18 | Overshot | Ströbersmühle | Friesenbach | Rhine | Germany |
| 18 | Overshot | Obere Strohner Mühle |  | Rhine | Germany |
| 18 | Overshot | Schäferkämper Mühle |  | Rhine | Germany |
| 18 | Overshot | Obere Rostmühle |  | Rhine | Germany |
| 18 | Overshot | Berger Mühle | Broicher Bach | Rhine | Germany |
| 18 | Overshot | Oberste Mühle/ Heidter Mühle |  | Rhine | Germany |
| 18 | Overshot | Ölers Mühle |  | Rhine | Germany |
| 18 | Overshot | Alter Hammer | Honne | Rhine | Germany |
| 18 | Overshot | Schulten Muhle |  | Rhine | Germany |
| 18 | Overshot | Schönen Mühle | Mühlenteich | Rhine | Germany |
| 18 | Overshot | Wambacher Mühle |  | Rhine | Germany |
| 18 | Overshot | Alte SchloBmuhle |  | Rhine | Germany |
| 18 | Overshot | Bauersmuhle | Neffelbach | Rhine | Germany |
| 18 | Overshot | Lindenberger Mühle | Neuer Ellbach | Rhine | Germany |
| 18 | Overshot | Landwasser Hofmühle |  | Rhine | Germany |
| 18 | Overshot | Mühle im Hübschental |  | Rhine | Germany |
| 18 | Overshot | Alte Mühle/ Pauli Mühle |  | Rhine | Germany |
| 18 | Overshot | Burgmuhle Bergerhausen | Neffelbach | Rhine | Germany |
| 18 | Overshot | Tröndlemühle |  | Rhine | Germany |
| 18 | Overshot | Unterschembachhofmühle |  | Rhine | Germany |
| 18 | Overshot | Fallerhofmühle of Floßmühle |  | Rhine | Germany |
| 18 | Overshot | Corrensmuhle/Mittlere Muhle/Keips Muhle | Gleueler Bach | Rhine | Germany |
| 18 | Overshot | Olmuhle |  | Rhine | Germany |
| 18 | Overshot | Eisenhammer | Haselbach | Rhine | Germany |
| 18 | Overshot | Üdersdorfer Mühle | Lieser | Rhine | Germany |
| 18 | Overshot | Jägertonihofmühle |  | Rhine | Germany |
| 18 | Overshot | Decker's Mühle |  | Rhine | Germany |
| 18 | Overshot | Aichhalder Mühle |  | Rhine | Germany |
| 18 | Overshot | Kornmühle Urishof |  | Rhine | Germany |
| 18 | Overshot | Obere Starkenburger Mühle |  | Rhine | Germany |
| 18 | Overshot | Oelchenshammer |  | Rhine | Germany |
| 18 | Overshot | Hubertus Muhle |  | Rhine | Germany |
| 18 | Overshot | Hugenhofmühle |  | Rhine | Germany |
| 18 | Overshot | Benzmühle |  | Rhine | Germany |
| 18 | Overshot | Kronen Mühle |  | Rhine | Germany |
| 18 | Overshot | Alte Muhle | Bruckhauser Muhlenbach | Rhine | Germany |
| 18 | Undershot | Ettaler Mühle |  | Rhine | Germany |
| 18 | Undershot | Ölmühle |  | Rhine | Germany |
| 18 | Undershot | Hausmanns Muhle | Schwarzbach | Rhine | Germany |
| 18 | Undershot | Gysenberger Muhle |  | Rhine | Germany |
| 18 | Undershot | Pletschmühle | Kranenbach (Schwalm) | Rhine | Germany |
| 18 | Undershot | Ölmühle Sankt Julian |  | Rhine | Germany |
| 18 | Undershot | Rosselmühle |  | Rhine | Germany |
| 18 | Undershot | Leuther Mühle | Nette | Rhine | Germany |
| 18 | Undershot | Hammerschmiede am Blautopf |  | Rhine | Germany |
| 18 | Undershot | Lutke Muhle / Rolinck's |  | Rhine | Germany |
| 18 | Undershot | Luisenhutte Wocklum |  | Rhine | Germany |
| 18 | Undershot | Balkhause Kotten | Wupper | Rhine | Germany |
| 18 | Undershot | Mühle des Hauses Döring |  | Rhine | Germany |
| 18 | Undershot | Wolbers Mühle | Dinkel | Rhine | Germany |
| 18 | Undershot | 's Glatze Mühle |  | Rhine | Germany |
| 18 | Undershot | Bremecker Hammer |  | Rhine | Germany |
| 18 | Undershot | Ölmühle Michelau |  | Rhine | Germany |
| 18 | Undershot | Frauenhofsche Mühle/ Mittelmühle | Itterbach | Rhine | Germany |
| 18 | Undershot | Puhesche Mühle/ Ganzendagsche Mühle | Borkener Aa | Rhine | Germany |
| 18 | Undershot | Kocks Mühle |  | Rhine | Germany |
| 18 | Undershot | Ölmühle |  | Rhine | Germany |
| 18 | Undershot | Cromford Muhle | Angerbach | Rhine | Germany |
| 18 | Undershot | Wassermühle Knollmann | Hörsteler Aa | Rhine | Germany |
| 18 | Undershot | Olmuhle Salzkotten |  | Rhine | Germany |
| 18 | Undershot | frohnsmuhle | Lendesdorfer Muhlenteich | Rhine | Germany |
| 18 | Undershot | Bocketsmühle | Baalbach | Rhine | Germany |
| 18 | Unknown | Kraemer Mühle |  | Rhine | Germany |
| 18 | Unknown | SchloBmuhle |  | Rhine | Germany |
| 18 | Unknown | Ölmühle Jäger |  | Rhine | Germany |
| 18 | Unknown | Hilfinger Mühle |  | Rhine | Germany |
| 18 | Unknown | Lettlmühle |  | Rhine | Germany |
| 18 | Unknown | Rauberweiher Mühle |  | Rhine | Germany |
| 18 | Unknown | Neunkirchener Mühle | Pützbornerbach | Rhine | Germany |
| 18 | Unknown | Bechermuhle | Lochbach | Rhine | Germany |
| 18 | Unknown | Locher Hammer | lochbach | Rhine | Germany |
| 18 | Unknown | Poscheider Muhle | Lochbach | Rhine | Germany |
| 18 | Unknown | Scheider Muhle | Lochbach | Rhine | Germany |
| 18 | Unknown | Kringsmuhle | Schwalm | Rhine | Germany |
| 18 | Unknown | Bleckhausener Mühle | Kleinen Kyll | Rhine | Germany |
| 18 | Unknown | Lamgesmuhle | Murbach | Rhine | Germany |
| 18 | Unknown | Wickrathberger Mühle | Niers | Rhine | Germany |
| 18 | Unknown | Schönheitsmühle | Schwarzbach | Rhine | Germany |
| 18 | Unknown | Fuchsmühle |  | Rhine | Germany |
| 18 | Unknown | Mittelmühle |  | Rhine | Germany |
| 18 | Unknown | Riedmühle | Riedmühlenbach | Rhine | Germany |
| 18 | Unknown | Kügler Mühle |  | Rhine | Germany |
| 18 | Unknown | Würschhauser Mühle |  | Rhine | Germany |
| 18 | Unknown | Konradsmühle |  | Rhine | Germany |
| 18 | Unknown | Bausmuhle |  | Rhine | Germany |
| 18 | Unknown | Scheffenmühle | Schwarzbach | Rhine | Germany |
| 18 | Unknown | Mühle Schlotmann |  | Rhine | Germany |
| 18 | Unknown | Diethelmmühle |  | Rhine | Germany |
| 18 | Unknown | Lohmühle | Niers | Rhine | Germany |
| 18 | Unknown | Mühle Cordes |  | Rhine | Germany |
| 18 | Unknown | Fuchsmühle |  | Rhine | Germany |
| 18 | Unknown | Emsmuhle | Ems | Rhine | Germany |
| 18 | Unknown | Aubenmühle |  | Rhine | Germany |
| 18 | Unknown | Verings Mühle | Werse | Rhine | Germany |
| 18 | Unknown | Ölmühle |  | Rhine | Germany |
| 18 | Unknown | Kepplermuhle | Lennefe | Rhine | Germany |
| 18 | Unknown |  |  | Rhine | Germany |
| 18 | Unknown | Grafenmühle | Rotbach | Rhine | Germany |
| 18 | Unknown | Glattenzainbach Mühle |  | Rhine | Germany |
| 18 | Unknown | Untere Papiermühle | Krauthausener Mühlenteich | Rhine | Germany |
| 18 | Unknown | Adler Mühle | Dreisam | Rhine | Germany |
| 18 | Unknown | Gutmühle | Wahnbach | Rhine | Germany |
| 18 | Unknown | Deymannsmühle | Hohe Ley | Rhine | Germany |
| 18 | Unknown | Wackers Mühle | Kirchberger Mühlenteich | Rhine | Germany |
| 18 | Unknown | Öhlerhofmühle | Siedelbach | Rhine | Germany |
| 18 | Unknown | Kornmuhle | Schleborn-Bach | Rhine | Germany |
| 18 | Unknown | Kugelmühle |  | Rhine | Germany |
| 18 | Unknown | Ernenkotten | Nacker Bach | Rhine | Germany |
| 18 | Unknown | Kimmelmuhle | Hammer Bach | Rhine | Germany |
| 18 | Unknown | Papiermuhle | Angerbach | Rhine | Germany |
| 18 | Unknown | Gronenborner Mühle | Leimbach | Rhine | Germany |
| 18 | Unknown | Altvogtsmühle |  | Rhine | Germany |
| 18 | Unknown | Dustermuhl | Dinkel | Rhine | Germany |
| 18 | Unknown | Grunder Muhle |  | Rhine | Germany |
| 18 | Unknown | Porselener Mühle | Wurm | Rhine | Germany |
| 18 | Unknown | Unterheiligenhovener Muhle |  | Rhine | Germany |
| 18 | Unknown | wassermuhle Schluter |  | Rhine | Germany |
| 18 | Unknown | Ingentaler Muhle | Rodebach | Rhine | Germany |
| 18 | Unknown | Papierfabrik Schleipen & Erkens | Kirchberger Mühlenteich | Rhine | Germany |
| 18 | Unknown | Sprinker Mühle |  | Rhine | Germany |
| 18 | Unknown | Steiner's Mühle |  | Rhine | Germany |
| 18 | Unknown | Michelishof Mühle |  | Rhine | Germany |
| 18 | Breast and Overshot | Bovenste Plasmolen | Molenbeek | Meuse | Netherlands |
| 18 | Breastshot | Neuborgmolen / Molen van Roex | Gulp | Meuse | Netherlands |
| 18 | Breastshot | Ophovenermolen | Geleenbeek | Meuse | Netherlands |
| 18 | Breastshot | Gitstappermolen | Roode Beek | Meuse | Netherlands |
| 18 | Breastshot | Kasteelmolen | Selzerbeek | Meuse | Netherlands |
| 18 | Breastshot | Kathagermolen | Geleenbeek | Meuse | Netherlands |
| 18 | Overshot | Bulkemsmolen | Eyerbeek | Meuse | Netherlands |
| 18 | Overshot | Ronckenstein molen | Schellekensbeek | Meuse | Netherlands |
| 18 | Overshot | De Bovenste molen |  | Meuse | Netherlands |
| 18 | Overshot | Caumermolenweg | Caumerbeek | Meuse | Netherlands |
| 18 | Undershot |  | Vloet | Meuse | Netherlands |
| 18 | Undershot | Hooydonkse Watermolen | Dommel | Meuse | Netherlands |
| 18 | Undershot | Broekmolen | Aabeek | Meuse | Netherlands |
| 18 | Undershot | Opwettense Watermolen | Kleine Dommel | Meuse | Netherlands |
| 18 | Undershot | De watermolen van Wanssum | Oostrumsebeek | Meuse | Netherlands |
| 18 | Undershot | Molen van Wolfsewinkel | Dommel | Meuse | Netherlands |
| 18 | Undershot | Leumolen of St. Ursulamolen | Tungelroyse Beek | Meuse | Netherlands |
| 18 | Undershot | Molen Otten | Geul | Meuse | Netherlands |
| 18 | Undershot | De Vogelmolen | Haelense Beek | Meuse | Netherlands |
| 18 | Undershot | De Collse Watermolen | Kleine Dommel | Meuse | Netherlands |
| 18 | Undershot | Molen op de Swalm |  | Meuse | Netherlands |
| 18 | Undershot | Graanmolen van Eijsden | Voer | Meuse | Netherlands |
| 18 | Undershot | Weverijmuseum | Dommel | Meuse | Netherlands |
| 18 | Undershot | Uffelse Molen | Uffelse Beek | Meuse | Netherlands |
| 18 | Unknown | Zaagwatermolen | Voer | Meuse | Netherlands |
| 18 | Unknown | Hertogsmolen |  | Meuse | Netherlands |
| 18 | Unknown | De molen van Frankenhof | Zieversbeek | Meuse | Netherlands |
| 18 | Unknown | Walkmolen | Niers | Meuse | Netherlands |
| 18 | Unknown | De Koniklijke Walkmolen | Niers | Meuse | Netherlands |
| 18 | Unknown | Volmolen | Rode Beek | Meuse | Netherlands |
| 18 | Unknown | Onderste Molen | Rode Beek | Meuse | Netherlands |
| 18 | Unknown | Watermolen de Ancker/Papiermolen | Jeker | Meuse | Netherlands |
| 18 | Unknown | Breustermolen | Voer | Meuse | Netherlands |
| 18 | Overshot | Molen Quadenoord 1 | Renkumsebeek | Rhine | Netherlands |
| 18 | Overshot | Molen van Bels | Mosbeek | Rhine | Netherlands |
| 18 | Overshot | Molen Van Frans | Mosbeek | Rhine | Netherlands |
| 18 | Overshot | De Kopermolen | Klaarbeek | Rhine | Netherlands |
| 18 | Overshot | De Zuukermolen | Klaarbeek | Rhine | Netherlands |
| 18 | Overshot | Nagedacht |  | Rhine | Netherlands |
| 18 | Overshot | Goedgedacht |  | Rhine | Netherlands |
| 18 | Undershot | Waterradmolen te Vorden | Vordense beek | Rhine | Netherlands |
| 18 | Undershot |  |  | Rhine | Netherlands |
| 18 | Undershot | markvelderwatermolen | Schipbeek | Rhine | Netherlands |
| 18 | Undershot | De Mallumsche Molen | Berkel | Rhine | Netherlands |
| 18 | Undershot | Berenschot | Aaltense Slinge | Rhine | Netherlands |
| 18 | Undershot | De Olliemolle | Zijtak van de Berkel | Rhine | Netherlands |
| 18 | Undershot | De Stenen Tafel | Zijtak van de Berkel | Rhine | Netherlands |
| 18 | Unknown | Het Gellegat | Leuvenumsebeek | Rhine | Netherlands |
| 18 | Unknown | Molen bij de Vispoort |  | Rhine | Netherlands |
| 18 | Unknown | Oostelijke Ottermolen | Leuvenumsebeek | Rhine | Netherlands |
| 18 | Unknown | Westelijke Ottermolen | Leuvenumsebeek | Rhine | Netherlands |
| 18 | Unknown | De Zandmolen 2 | Leuvenumsebeek | Rhine | Netherlands |
| 18 | Unknown | Het Heilige Huis 1 | Leuvenumsebeek | Rhine | Netherlands |
| 18 | Unknown | Het Heilige Huis 2 | Leuvenumsebeek | Rhine | Netherlands |
| 18 | Unknown | Hessenmolen | Leuvenumsebeek | Rhine | Netherlands |
| 19 | Breastshot | Moulin Schins | Geule | Meuse | Belgium |
| 19 | Breastshot | Moulin d-en Bas | Leupont | Meuse | Belgium |
| 19 | Breastshot | Moulin d-Ichebroux, Moulin Fontaine | Le Train | Meuse | Belgium |
| 19 | Breastshot | Moulin de Royseux | Hoyoux | Meuse | Belgium |
| 19 | Overshot | Moulin de la Gouge, Moulin de la Gouche Spixhe | Le Wayai | Meuse | Belgium |
| 19 | Overshot | Moulin de Goyet | Samson | Meuse | Belgium |
| 19 | Overshot | Filature de Wayse, Maison Gaston Wets | Dijle | Meuse | Belgium |
| 19 | Overshot | Moulin Schumacher, Neumuhle | Iterbach en Periolbach | Meuse | Belgium |
| 19 | Overshot | Moulin de Boiron | Houille | Meuse | Belgium |
| 19 | Overshot | Molen van Frisen | Voer | Meuse | Belgium |
| 19 | Overshot | Stayenmolen / Vettersmolen | Molenbeek | Meuse | Belgium |
| 19 | Overshot | Moulin d'Audince | Petite Gette | Meuse | Belgium |
| 19 | Overshot | Moulin Scheid | Ourthe | Meuse | Belgium |
| 19 | Overshot | Moulin d-Halconreux | mayon | Meuse | Belgium |
| 19 | Overshot | Moulin Clotuche | Ourthe | Meuse | Belgium |
| 19 | Overshot | Moulin Magotiaux | Ourthe | Meuse | Belgium |
| 19 | Overshot | Moulin Spoo |  | Meuse | Belgium |
| 19 | Overshot | Moulin de Boeur |  | Meuse | Belgium |
| 19 | Overshot | Moulin de Lambieval | Ri de Tavigny | Meuse | Belgium |
| 19 | Overshot | Moulin Barthel | Cowan | Meuse | Belgium |
| 19 | Overshot | Moulin de lÉrmitage | Cowan | Meuse | Belgium |
| 19 | Overshot | Moulin de Wilogne | Martin Moulin | Meuse | Belgium |
| 19 | Overshot | Moulin de Nadrin | Belle-Meuse | Meuse | Belgium |
| 19 | Overshot | Moulin de l-Estree | la Legere Eau | Meuse | Belgium |
| 19 | Overshot | Moulin de Neufpre | Cowan | Meuse | Belgium |
| 19 | Overshot | Moulin d-en Haut |  | Meuse | Belgium |
| 19 | Overshot | Moulin Bonus, Le Vieux Moulin | Le Train | Meuse | Belgium |
| 19 | Overshot | Moulin de Mousty | Ry Angon | Meuse | Belgium |
| 19 | Overshot | Moulin de La Forge |  | Meuse | Belgium |
| 19 | Overshot | Moulin Valerienne, Moulin Debienne | Ri du Pre Delcourt | Meuse | Belgium |
| 19 | Overshot | Moulin Nys |  | Meuse | Belgium |
| 19 | Overshot | Moulin du Bouhet |  | Meuse | Belgium |
| 19 | Overshot | Moulin Guillaume | Oneu | Meuse | Belgium |
| 19 | Overshot | Moulin de Dison | Taureau | Meuse | Belgium |
| 19 | Overshot | Moulin d-Eveux |  | Meuse | Belgium |
| 19 | Overshot | Moulin d-Ossgne, Moulin Verlaine | Ruisseau d-Ossogne | Meuse | Belgium |
| 19 | Overshot | Vieux Moulin de Solieres |  | Meuse | Belgium |
| 19 | Overshot | Moulin d-Hoboval | Ri Fond d-Oxhe | Meuse | Belgium |
| 19 | Overshot | Ferme-Moulin Neys | Scherbach | Meuse | Belgium |
| 19 | Overshot | Moulin d-Awez, Moulin Seron | Estinale | Meuse | Belgium |
| 19 | Overshot | Moulin de Sart, Moulin Koos | Golnay | Meuse | Belgium |
| 19 | Overshot |  | La Magree | Meuse | Belgium |
| 19 | Overshot | Moulin de la Salette, Moulin Tchout |  | Meuse | Belgium |
| 19 | Overshot | Molen van Sinnich | Gulp | Meuse | Belgium |
| 19 | Overshot | Moulin de Hullscheid | Our | Meuse | Belgium |
| 19 | Overshot | Moulin de Jalhay | Hoegne | Meuse | Belgium |
| 19 | Overshot | Scierie Remy | Tavigny | Meuse | Belgium |
| 19 | Overshot | Moulin de Neufmoulin | Cowan | Meuse | Belgium |
| 19 | Overshot | Moulin dussart | Housiere | Meuse | Belgium |
| 19 | Overshot | Moulin de jamagne, Moulin Henin |  | Meuse | Belgium |
| 19 | Overshot | Moulin Valois | Grand Aaz | Meuse | Belgium |
| 19 | Overshot | Moulin de Cherapont, moulin Boulanger | Ourthe | Meuse | Belgium |
| 19 | Overshot | Moulin de Veve, Moulin du Chateau | Veve | Meuse | Belgium |
| 19 | Overshot | Moulin de Warnon |  | Meuse | Belgium |
| 19 | Overshot | Moulin a Chicoree | Thyria | Meuse | Belgium |
| 19 | Overshot | Moulin de Ciplet |  | Meuse | Belgium |
| 19 | Overshot | Moulin d-Haut | La Thyle (Dijle) | Meuse | Belgium |
| 19 | Overshot | Moulin Caba | Rue du Tordoir 34 | Meuse | Belgium |
| 19 | Overshot | Scierie Andre | Mayon | Meuse | Belgium |
| 19 | Overshot | Centrale electrique | Bocq | Meuse | Belgium |
| 19 | Overshot | Scierie Collard, Scierie Cocole |  | Meuse | Belgium |
| 19 | Overshot | Moulin | Bocq | Meuse | Belgium |
| 19 | Turbine | Moulin Chinz | Geul | Meuse | Belgium |
| 19 | Turbine | Moulin de la Roche, Moulin Ladriere | Dijle | Meuse | Belgium |
| 19 | Turbine | Wewelermuhle | Our | Meuse | Belgium |
| 19 | Turbine | Ulftaler Schenke | Ulf | Meuse | Belgium |
| 19 | Turbine | Moulin de Cibru | Wez | Meuse | Belgium |
| 19 | Turbine | Moulin de Belle-Meuse | Belle-Meuse | Meuse | Belgium |
| 19 | Turbine | Moulin Motte |  | Meuse | Belgium |
| 19 | Turbine | Moulin Del Planque |  | Meuse | Belgium |
| 19 | Turbine | Nouveau Moulin, Moulin de Grosage | Le Domissart | Meuse | Belgium |
| 19 | Turbine | Moulin Conard | grote Gete | Meuse | Belgium |
| 19 | Turbine | Bookmolen | Roosterbeek | Meuse | Belgium |
| 19 | Turbine | Oestegemuhle | Hoegne | Meuse | Belgium |
| 19 | Turbine | Moulin Rouge |  | Meuse | Belgium |
| 19 | Turbine | cafe de la Turbine |  | Meuse | Belgium |
| 19 | Turbine | Petit Moulin d-Arenberg | zenne | Meuse | Belgium |
| 19 | Turbine | Nieuwbeekmolen | Demer | Meuse | Belgium |
| 19 | Turbine | moulin wierinckx | Dijle | Meuse | Belgium |
| 19 | Turbine | Moulin Coqneau | Pont Tordoir | Meuse | Belgium |
| 19 | Turbine | Pompmolentje | Herk | Meuse | Belgium |
| 19 | Turbine | Moulin d-Ombret | Fond d-Oxhe | Meuse | Belgium |
| 19 | Undershot | Moulin d-harre | Harre | Meuse | Belgium |
| 19 | Undershot | Engelingenmolen | Herk | Meuse | Belgium |
| 19 | Undershot | Crombachermuhle | Mittelbach | Meuse | Belgium |
| 19 | Undershot | Moulin Ponsart |  | Meuse | Belgium |
| 19 | Undershot | Moulin Nelhain | Berwinne | Meuse | Belgium |
| 19 | Undershot | Moulin d-Hodbomont | Le Wayot | Meuse | Belgium |
| 19 | Undershot | Moulin Dangoneau | Thisnes | Meuse | Belgium |
| 19 | Undershot | Motmolen | Jeker | Meuse | Belgium |
| 19 | Undershot | Brulmolen | Zwarte Beek | Meuse | Belgium |
| 19 | Undershot | Dorpsmolen, Robbenmolen, Vlasmolen |  | Meuse | Belgium |
| 19 | Undershot | Terlaemenmolen | Laambeek | Meuse | Belgium |
| 19 | Undershot | Moulin du Pave | Train | Meuse | Belgium |
| 19 | Undershot | Moulin de la Biesmelle |  | Meuse | Belgium |
| 19 | Undershot | Moulin d-Amonines |  | Meuse | Belgium |
| 19 | Undershot | Moulin Beaurieu, Moulin Dussart | Orne | Meuse | Belgium |
| 19 | Undershot | Moulin de Limal | Dijle | Meuse | Belgium |
| 19 | Undershot | Moulin de la Caiade, mOUlin du pont des Oyes | Rau Henri Fontaine | Meuse | Belgium |
| 19 | Undershot | Broekkantmolen | Warmbeek | Meuse | Belgium |
| 19 | Undershot | Hammolen | Herk | Meuse | Belgium |
| 19 | Undershot | Moulin Cambier | Semois | Meuse | Belgium |
| 19 | Undershot | Moulin de la Cheneviere |  | Meuse | Belgium |
| 19 | Undershot | Moulin Lhermitte |  | Meuse | Belgium |
| 19 | Undershot | Moulin-scierie Clement | Ton | Meuse | Belgium |
| 19 | Undershot | Moulin de Velupont |  | Meuse | Belgium |
| 19 | Undershot | Rooiermolen | Itterbeek | Meuse | Belgium |
| 19 | Undershot | Atelier Pirson, Moulin Forge |  | Meuse | Belgium |
| 19 | Undershot | Molen Dewerd | Warmbeek | Meuse | Belgium |
| 19 | Undershot | "Machine a elever les eaux de Porcheresse" | Almache | Meuse | Belgium |
| 19 | Undershot | Machine Hydraulique | Aisne | Meuse | Belgium |
| 19 | Breastshot | Voglersche Hammerschmiede |  | Rhine | Germany |
| 19 | Breastshot | Werthes Mühle | Haune | Rhine | Germany |
| 19 | Breastshot | Hammerschmiede |  | Rhine | Germany |
| 19 | Breastshot | Grafenmühle |  | Rhine | Germany |
| 19 | Breastshot |  |  | Rhine | Germany |
| 19 | Overshot | Vollmers Mühle |  | Rhine | Germany |
| 19 | Overshot | Drahtrolle Am Hurk | Springen | Rhine | Germany |
| 19 | Overshot | Zuckernbauerhof Mühle |  | Rhine | Germany |
| 19 | Overshot | Fröhnder Klopfsäge |  | Rhine | Germany |
| 19 | Overshot | Hartings Muhle | Muhlenteich | Rhine | Germany |
| 19 | Overshot | Muhle Vossiek | Linnenbeeke | Rhine | Germany |
| 19 | Overshot | Tischlerei Sturhan / Hermannsmuhle | Linnenbeeke | Rhine | Germany |
| 19 | Overshot | Wassermuhle Ramsbeck |  | Rhine | Germany |
| 19 | Overshot | Gillenfelder Mühle/ Hubertusmühle |  | Rhine | Germany |
| 19 | Overshot | Barweiler Mühle | Wirftbach | Rhine | Germany |
| 19 | Overshot | Muhle Nienhage | Linnenbeeke | Rhine | Germany |
| 19 | Overshot | Domteichmühle/ Drewelsmühle | Linnenbeeke | Rhine | Germany |
| 19 | Overshot | Dummlinghauser |  | Rhine | Germany |
| 19 | Overshot | Kobisen Mühle |  | Rhine | Germany |
| 19 | Overshot | Muhle Starke |  | Rhine | Germany |
| 19 | Overshot | Romesmuhle | Golkrather Bach | Rhine | Germany |
| 19 | Overshot |  |  | Rhine | Germany |
| 19 | Overshot | Kaiserhofmühle |  | Rhine | Germany |
| 19 | Overshot | wassermuhle Westerrode | Stever | Rhine | Germany |
| 19 | Overshot | Hexenloch Mühle |  | Rhine | Germany |
| 19 | Overshot | Hammerschmiede | Linnenbeeke | Rhine | Germany |
| 19 | Overshot | Sagemuhle Haus marck |  | Rhine | Germany |
| 19 | Overshot | Vögele's Mühle |  | Rhine | Germany |
| 19 | Overshot | Strasserhofmühle |  | Rhine | Germany |
| 19 | Overshot | Stampfmuhle Marschallshagen |  | Rhine | Germany |
| 19 | Overshot | Werfer Muhle |  | Rhine | Germany |
| 19 | Overshot | Daniele Mühle |  | Rhine | Germany |
| 19 | Overshot | Klingenhofsäge |  | Rhine | Germany |
| 19 | Overshot | Untere Mühle |  | Rhine | Germany |
| 19 | Overshot | Brenscheider Olmuhle | Nahmerbach | Rhine | Germany |
| 19 | Overshot | Hasenmühle | Frohnbach | Rhine | Germany |
| 19 | Overshot | Gutmannhofmühle |  | Rhine | Germany |
| 19 | Overshot | Muhle Gerker | Rehmerloh-Menninghuffer Muhlenbach | Rhine | Germany |
| 19 | Overshot | Schmiede Balt | Arfebach | Rhine | Germany |
| 19 | Overshot | Knochen Muble | Esselbach | Rhine | Germany |
| 19 | Overshot | Wiegele's Mühle |  | Rhine | Germany |
| 19 | Overshot | Melcherhof Mühle |  | Rhine | Germany |
| 19 | Overshot | Exmühle (Aixmühle) | Adenauerbach | Rhine | Germany |
| 19 | Overshot | Obere Muhle Dahlem |  | Rhine | Germany |
| 19 | Overshot | Benzmühle am Bach |  | Rhine | Germany |
| 19 | Overshot | Finkenberger Muhle | Schwarzenbruch Bach | Rhine | Germany |
| 19 | Overshot | Klostermuhle |  | Rhine | Germany |
| 19 | Overshot | Schwanen Mühle |  | Rhine | Germany |
| 19 | Overshot | Hagmühle |  | Rhine | Germany |
| 19 | Overshot | Höllmühle |  | Rhine | Germany |
| 19 | Overshot | Wennefelder Mühle | Armutsbach | Rhine | Germany |
| 19 | Overshot | Wehrlehofmühle |  | Rhine | Germany |
| 19 | Overshot | Wassermuhle am LaBbach | LaBbach | Rhine | Germany |
| 19 | Overshot | Großjockenmühle |  | Rhine | Germany |
| 19 | Overshot | Monchermuhle |  | Rhine | Germany |
| 19 | Overshot | Öl-, Mahl- und Sägemühle |  | Rhine | Germany |
| 19 | Overshot | Bühler Mühle |  | Rhine | Germany |
| 19 | Overshot |  |  | Rhine | Germany |
| 19 | Overshot | Muhle Hoberg | Maasbeeke | Rhine | Germany |
| 19 | Overshot | Knochenmuhle Fretter |  | Rhine | Germany |
| 19 | Overshot | Heilerhofmühle |  | Rhine | Germany |
| 19 | Overshot | Berbardsmuhle |  | Rhine | Germany |
| 19 | Undershot | Mühle vom Kappelhof |  | Rhine | Germany |
| 19 | Undershot | Flederichsmühle |  | Rhine | Germany |
| 19 | Undershot | Umgrover Muhle | Mussenbach | Rhine | Germany |
| 19 | Undershot | Nuning'sche Wassermuhle |  | Rhine | Germany |
| 19 | Undershot | Gschwender Mühle |  | Rhine | Germany |
| 19 | Undershot | Ölmühle Waltz |  | Rhine | Germany |
| 19 | Undershot | Ölschlagmühle Wiesthal |  | Rhine | Germany |
| 19 | Undershot | Holtmannsmuhle | Gerlingbach | Rhine | Germany |
| 19 | Undershot | Springiersbacher Mühle | Alf | Rhine | Germany |
| 19 | Undershot | Schneidemühle |  | Rhine | Germany |
| 19 | Undershot | Mühle am Rain/ Rainbauernmühle |  | Rhine | Germany |
| 19 | Undershot | Stadtmuhle / Bertramsmuhle | Muhlengraden | Rhine | Germany |
| 19 | Undershot | Hofmuhle Frye | Stever | Rhine | Germany |
| 19 | Undershot | Hochgangsäge |  | Rhine | Germany |
| 19 | Unknown | Mühle Klapprich | Weibernerbach | Rhine | Germany |
| 19 | Unknown | Unterbrucher Muhle | Wurm | Rhine | Germany |
| 19 | Unknown | Kranentalsmühle | Broicher Bach | Rhine | Germany |
| 19 | Unknown | Sägmühle |  | Rhine | Germany |
| 19 | Unknown | Muhle von Schulze Egbeiding |  | Rhine | Germany |
| 19 | Unknown | Wassermuhle Eichenthal | Wurm | Rhine | Germany |
| 19 | Unknown | Molitors Mühle |  | Rhine | Germany |
| 19 | Unknown | Furthmühle |  | Rhine | Germany |
| 19 | Unknown | Breidterstraagsmuhle | Jabach | Rhine | Germany |
| 19 | Unknown | Lennemuhle Gilsbach |  | Rhine | Germany |
| 19 | Unknown | Neuemuhle |  | Rhine | Germany |
| 19 | Unknown | Laubenheimer mühle |  | Rhine | Germany |
| 19 | Unknown | Muhle Sonntag | Linnenbeeke | Rhine | Germany |
| 19 | Unknown | Schneidersmühle | Endert | Rhine | Germany |
| 19 | Unknown | Knochenmuhle |  | Rhine | Germany |
| 19 | Unknown | Muhle Heinemann | Wenne | Rhine | Germany |
| 19 | Unknown | Drahtrolle Kayser |  | Rhine | Germany |
| 19 | Unknown | Neumühle | Woogbach | Rhine | Germany |
| 19 | Unknown | Brücker Mühle |  | Rhine | Germany |
| 19 | Unknown | Millener Muhle | Rodebach | Rhine | Germany |
| 19 | Unknown | Lochter Wassermuhle | Lochterbach | Rhine | Germany |
| 19 | Unknown | Niederdielfener Muhle |  | Rhine | Germany |
| 19 | Unknown | Wassermuhle Worpenberg |  | Rhine | Germany |
| 19 | Unknown | Iddelsfelder Mühle | Strunderbach | Rhine | Germany |
| 19 | Unknown | Köninger Mühle |  | Rhine | Germany |
| 19 | Unknown | Mühle vom Obermaierhof |  | Rhine | Germany |
| 19 | Unknown | Marhördter Sägmühl |  | Rhine | Germany |
| 19 | Unknown | Röschmühle/ Mühle Schulze | Simmersbach | Rhine | Germany |
| 19 | Unknown | Kuhlheimer Muhle |  | Rhine | Germany |
| 19 | Unknown | Alte Ölmühle |  | Rhine | Germany |
| 19 | Unknown | Untermühle |  | Rhine | Germany |
| 19 | Unknown | Rengser Muhle |  | Rhine | Germany |
| 19 | Unknown | Untere Mühle/ Quirinsmühle |  | Rhine | Germany |
| 19 | Unknown | Züscher Mühle |  | Rhine | Germany |
| 19 | Unknown | Bruningsmuhle | Vechte | Rhine | Germany |
| 19 | Unknown | Brucker Muhle | Angerbach | Rhine | Germany |
| 19 | Unknown | Rubberts Muhle |  | Rhine | Germany |
| 19 | Unknown |  | Aa | Rhine | Germany |
| 19 | Unknown |  |  | Rhine | Germany |
| 19 | Unknown | Neue Senfmuhle |  | Rhine | Germany |
| 19 | Unknown | Danielsmuhle | Veebach | Rhine | Germany |
| 19 | Unknown | Kemenas Muhle | Mittelbach | Rhine | Germany |
| 19 | Unknown | Brockmuhle | Heubach | Rhine | Germany |
| 19 | Unknown | Gemener Stadtmühle | Aa | Rhine | Germany |
| 19 | Unknown | Schmälze Mühle |  | Rhine | Germany |
| 19 | Unknown | Schmuttermühle | Schmutter | Rhine | Germany |
| 19 | Breastshot | Kruitmolen | Geul | Meuse | Netherlands |
| 19 | Breastshot | Bovenste Molen | Geul | Meuse | Netherlands |
| 19 | Breastshot | Wittemermolen | Selzerbeek | Meuse | Netherlands |
| 19 | Breastshot | Nieuwe molen / Ijzeren Molen | Geul | Meuse | Netherlands |
| 19 | Breastshot | Oliemolen | Geulke | Meuse | Netherlands |
| 19 | Breastshot | Volmolen | Geul | Meuse | Netherlands |
| 19 | Breastshot | Muggemolen | Voer | Meuse | Netherlands |
| 19 | Overshot | Bovenste Molen van Hulsen | Molenbeek | Meuse | Netherlands |
| 19 | Overshot | Schandelermolen | Caumerbeek | Meuse | Netherlands |
| 19 | Overshot | Puttersmolen | Voerendaalse molenbeek | Meuse | Netherlands |
| 19 | Undershot | Franse Molen | Geul | Meuse | Netherlands |
| 19 | Undershot | Oliemolen | Grote Molenbeek | Meuse | Netherlands |
| 19 | Undershot | Danikermolen | Geleenbeek | Meuse | Netherlands |
| 19 | Undershot | Molen van Gijsel | Reuzel | Meuse | Netherlands |
| 19 | Undershot | Spoordonkse Watermolen | Beerze | Meuse | Netherlands |
| 19 | Undershot | Grathemermolen | Uffelse Beek | Meuse | Netherlands |
| 19 | Undershot | De Kraekermolen | Thornerbeek | Meuse | Netherlands |
| 19 | Undershot | De Gennepermolen | Niers | Meuse | Netherlands |
| 19 | Undershot | Hammermolen | Neerbeek | Meuse | Netherlands |
| 19 | Unknown | Dieterdermolen | Rode Beek | Meuse | Netherlands |
| 19 | Unknown | Maasmolen op de Maas |  | Meuse | Netherlands |
| 19 | Unknown | Hochstenbach | Molenbeek | Meuse | Netherlands |
| 19 | Unknown | Eper / Wingbergermolen | Geul | Meuse | Netherlands |
| 19 | Unknown | Millenermolen | Rode Beek | Meuse | Netherlands |
| 19 | Unknown | Vlodroppermolen | Rode Beek | Meuse | Netherlands |
| 19 | Unknown | De Armenmolen / De Nieuwestadter Molen | Geleenbeek | Meuse | Netherlands |
| 19 | Overshot | Papiermolen Klarenbeek |  | Rhine | Netherlands |
| 19 | Overshot | Molen van Hazelbekke | Oude Beek / Hazelbekke | Rhine | Netherlands |
| 19 | Overshot | Van Lennepsmolen | Velpse of Rozendaalse Beek | Rhine | Netherlands |
| 19 | Overshot |  | Stuwvijver met naamloze sprengen | Rhine | Netherlands |
| 19 | Overshot | Peeske |  | Rhine | Netherlands |
| 19 | Overshot | De Middelste Molen | Voorsterbeek | Rhine | Netherlands |
| 19 | Undershot |  | Vm. Oude Ijssel | Rhine | Netherlands |
| 19 | Undershot | Watermolen van Singraven | Dinkel | Rhine | Netherlands |
| 19 | Unknown | Watermolen van Wijnen |  | Rhine | Netherlands |
| 19 | Unknown | Matebroeker molen |  | Rhine | Netherlands |
| 19 | Unknown | Watermolen aan de Lutters Ziel | Lutters Ziel | Rhine | Netherlands |
| 19 | Unknown | Molen aan de Rioekelseweg |  | Rhine | Netherlands |
| 20 | Overshot | Zangerheidemolen / Kasteelmolen | Kriekelbeek | Meuse | Belgium |
| 20 | Overshot | Scierie de Grande Hoursinne |  | Meuse | Belgium |
| 20 | Overshot | Moulin de Rettigny |  | Meuse | Belgium |
| 20 | Turbine | Moulin de Montignies-sur-Roc |  | Meuse | Belgium |
| 20 | Turbine | Moulin du Bois, Moulin a Tourettes | Rabion en Spambou | Meuse | Belgium |
| 20 | Breastshot | Hofmuhle Sierkertal | Osterbach | Rhine | Germany |
| 20 | Overshot | Hardmuhle / Holtmuhle |  | Rhine | Germany |
| 20 | Overshot | Mühlhauser Mühle |  | Rhine | Germany |
| 20 | Overshot | Wassermühle Dreimüllerhof |  | Rhine | Germany |
| 20 | Overshot | Getreidemühle |  | Rhine | Germany |
| 20 | Overshot | Muhle Zumbulte |  | Rhine | Germany |
| 20 | Overshot | Schloßmühle |  | Rhine | Germany |
| 20 | Overshot | Wassermühle Döhren | Mühlenteich | Rhine | Germany |
| 20 | Overshot | Sagermuhle | Kluckbach | Rhine | Germany |
| 20 | Overshot | Hasenhofmühle |  | Rhine | Germany |
| 20 | Overshot | Kirnachmühle |  | Rhine | Germany |
| 20 | Shipmill | Schliffsmuhle Minden | Weser | Rhine | Germany |
| 20 | Undershot | Hammerschmiede |  | Rhine | Germany |
| 20 | Unknown | Erkensmühle | Broicher Bach | Rhine | Germany |
| 20 | Unknown | Ruping's Muhle | Muhlenbeek | Rhine | Germany |
| 20 | Unknown | Niederstadtfelder Mühle | Kleinen Kyll | Rhine | Germany |
| 20 | Unknown | Auemuhle |  | Rhine | Germany |
| 20 | Breastshot | Klooster Redemptoristen | Sinzelbeek | Meuse | Netherlands |
| 20 | Overshot | Watervaldermolen / Pletsmolen / Graanmolen | Watervalderbeek | Meuse | Netherlands |
| 20 | Overshot | Groenendalsmolen | Gulp | Meuse | Netherlands |
| 20 | Undershot | Volmolen | Dommel | Meuse | Netherlands |
| 20 | Unknown | Geelmolen | Geleenmolensbeek | Meuse | Netherlands |
| 20 | Unknown | ECI-centrale |  | Meuse | Netherlands |
| 20 | Unknown | Groote, Oude of Banmolen | Geul | Meuse | Netherlands |
| 20 | Overshot | De Hamermolen | Koppelsprengen | Rhine | Netherlands |
| 20 | Overshot | De Hoop | Griftse Beek | Rhine | Netherlands |
| 20 | Overshot | Voorheen Achterste Molen Loenen | kunstmatig | Rhine | Netherlands |
| 20 | Overshot |  | Staverdense Beek | Rhine | Netherlands |
| 20 | Unknown | Cannenburgermolen | Hartensche Beek | Rhine | Netherlands |
| 21 | Overshot | Bouwhofmolen | Ughelse beek | Rhine | Netherlands |
